# Access to health insurance amongst people with disabilities and its association with healthcare use, health status and financial protection in low- and middle-income countries: a systematic review

**DOI:** 10.1186/s12939-024-02339-5

**Published:** 2024-12-18

**Authors:** Luthfi Azizatunnisa’, Hannah Kuper, Lena Morgon Banks

**Affiliations:** 1 International Centre for Evidence in Disability, London School of Hygiene and Tropical Medicine, London, UK; 2 Department of Health Behaviour, Environment and Social Medicine, Faculty of Medicine, Public Health and Nursing, Universitas Gadjah Mada, Yogyakarta, Indonesia

**Keywords:** Health equity, Universal Health Coverage, Disabled person, Inclusive health system

## Abstract

**Background:**

People with disabilities often incur higher costs for healthcare, due to higher needs, greater indirect costs, and the need for services not offered by the public system. Yet, people with disabilities are more likely to experience poverty and so have reduced capacity to pay. Health insurance is an important social protection strategy to meet healthcare needs and avoid catastrophic expenditures for this group. This systematic review synthesized evidence on health insurance coverage and potential effects among people with disabilities in low- and middle-income countries (LMICs).

**Methods:**

This systematic review followed PRISMA Guidelines. We searched English peer-reviewed articles from nine databases (Medline, Embase, CINAHL, Web of Science, Scopus, Cochrane Library, PsyInfo, Global Health, and Econlit) from January 2000 to 24 January 2023. Two independent reviewers conducted the article selection, data extraction, and risk of bias assessment using NIH Guidelines. Studies were eligible for inclusion if they quantitatively assessed at least one of four key outcomes amongst people with disabilities: health insurance coverage/access, the association between health insurance and health care utilization, financial protection, or health status/outcome. Narrative synthesis was deployed due to high variety of outcome measurements.

**Results:**

Out of 8,545 records retrieved and three from hand search, 38 studies covering data from 51 countries met the eligibility criteria. Over two-thirds (68.4%) focused on access/coverage, which was generally limited amongst people with disabilities. Seventeen studies (44.7%) examined healthcare utilization, with a positive association (9/12) found between health insurance and the use of disability-related services. However, its association with general healthcare utilization (5 studies) remained inconclusive. Financial protection, explored by six studies (15.8%), similarly yielded inconclusive results. Only four studies (10.5%) reported on health status, and the findings suggest a favourable association of health insurance with self-reported health among people with disabilities (2/4), despite the limited number of high-quality studies.

**Conclusions:**

There is considerable variability and limited evidence regarding health insurance coverage and its potential impact among individuals with disabilities in LMICs. This gap highlights the pressing need for further evaluations of health insurance, with a specific focus on people with disabilities, aligning with the broader goal of achieving Universal Health Coverage (UHC).

**Trial registration:**

PROSPERO CRD42023389533.

**Supplementary Information:**

The online version contains supplementary material available at 10.1186/s12939-024-02339-5.

## Background

Persons with disabilities are “those who have long-term physical, mental, intellectual or sensory impairments which in interaction with various barriers may hinder their full and effective participation in society on an equal basis with others” [1]. Worldwide, approximately 1.3 billion people, constituting 16% of the global population, are living with disabilities [2, 3]. Of this demographic, 80% reside in low- and middle-income countries (LMICs), and a significant proportion live in poverty [4, 5].

People with disabilities have triple health needs: general healthcare needs (e.g., promotive, preventive, curative care), similar to the rest of the population; health needs related to their impairments (e.g., specialistic healthcare, rehabilitation, assistive technology); and health needs for secondary health conditions tied to their impairment (e.g., pressure sore, urinary tract infection) [6–8]. Consequently, people with disabilities often require more healthcare services and higher health costs compared to those without disabilities [6, 9–13]. Nonetheless, people with disabilities typically encounter more challenges when seeking healthcare, including financial barriers to access [14–20]. They incur higher healthcare costs than the general population because of their higher healthcare needs [21], indirect expenses such as personal assistance and accessible transportation [8, 22, 23], and services beyond the scope of the public healthcare system. However, they are often less able to meet these costs, as they have a heightened risk of poverty and lack of health insurance or health insurance not covering their essential needs [10, 11, 24]. Indeed, evidence shows that people with disabilities are at a higher risk of catastrophic health expenditure (CHE) [7], which may push them further into poverty [25–32].

Health insurance can serve as a protective mechanism shielding individuals or households from the financial strain that could lead to impoverishment when seeking needed healthcare. Among the general population, health insurance has been associated with a decrease in out-of-pocket payment (OOP) [33–42], reduced reliance on negative financial coping mechanisms for health-related issues (e.g., borrowing money, selling assets, forgoing other needs) [33, 43, 44], and lower risk of incurring CHE [36, 45]. Additionally, evidence from the general population shows that health insurance is associated with enhanced access to healthcare [46, 47], increased healthcare utilization [34, 36, 38, 41, 46, 48, 49], and improved health outcomes [50–52]. Studies from high-income countries underscore the pivotal role of health insurance in facilitating access to healthcare services and assistive technology amongst individuals with disabilities [53–56]. However, the evidence regarding the coverage and potential impacts of health insurance for people with disabilities in LMICs remains limited and may differ for this group compared to the general population. Health insurance, for instance, might fall short in addressing their needs for impairment-related healthcare or adequately covering indirect costs of seeking care [15, 57]. Even with health insurance coverage, a significant gap may persist in healthcare access between individuals with disabilities and those without disabilities [56], as they face a range of additional barriers to seeking care.

Previous reviews have highlighted the scarcity of high-quality evidence on health insurance among individuals with disabilities in LMICs [58, 59]. However, newer data may have emerged in the 7–10 years since these reviews were conducted, particularly given the increased roll-out of health insurance programmes in LMICs. Therefore, this systematic review aims to comprehensively review existing evidence on health insurance coverage and its association with healthcare utilization, financial protection, and health status/outcomes among people with disabilities in LMICs.

## Methods

### Search

This systematic review followed PRISMA guidelines and had been registered in the Prospective Register of Systematic Reviews (PROSPERO; registration number: CRD42023389533). We searched nine databases (i.e., Medline, Embase, CINAHL, Web of Science, Scopus, Cochrane Library, PsyInfo, Global Health, and Econlit) using three broad terms—“people with disabilities” AND “health insurance” AND “low- and middle-income countries” on 24 January 2023. The search strategies used can be found in Additional file 1.

### Inclusion and exclusion criteria

The inclusion criteria were (1) peer-reviewed publications, (2) quantitative studies or quantitative components of mixed-methods studies including experimental, quasi-experimental, before-after, randomized control trial, cohort, case–control, or cross-sectional studies, (3) conducted in LMICs as defined by the World Bank in 2022, (4) written in English (based on the language ability of the reviewers), (5) evaluating at least one of the core outcomes (coverage, healthcare utilization, financial protection, or health status/outcomes), (6) published between January 2000 and January 2023, (7) the sample/population of people with disabilities or results disaggregated by disability, and (8) health insurance defined by the World Health Organization as including a contributory or non-contributory financing method that covers costs incurred from essential health services, including promotion, prevention, treatment, rehabilitation, and palliative care [60].

For coverage, we included both studies with and without comparisons. For outcomes related to healthcare utilization, financial protection, and health status/outcomes, we included only comparative studies.

We excluded articles where full-text access was unavailable, even after contacting the authors. Studies were also excluded if they did not disaggregate data by disability status or if they focused on alternative social protection strategies for partially covering health costs, such as fixed cash transfers. Additionally, we excluded articles that were not available in English.

### Study selection and data extraction

Two authors (LA and either LMB or HK) independently reviewed all records first by titles and abstracts and then by full text to determine the articles’ eligibility. A third reviewer assisted in resolving any disagreement.

Data extraction was conducted by LA using a matrix in an Excel spreadsheet and then checked by either LMB or HK. Information extracted included publication characteristics (i.e., author, published year, country/setting), study characteristics (i.e., study designs, sample size, sampling technique, and means of analysis), study population characteristics (age, sex, type of disability, disability measurement, comparison group), health insurance characteristics (type of insurance, whether targeting people with disabilities or mainstream), description of outcomes and their measurement (i.e., coverage, health utilization, financial protection, health status/outcomes) and measures of effect (e.g., prevalence ratios, odds ratios, or risk ratios for comparative studies or descriptive statistics for non-comparison studies).

### Risk of bias assessment

LA assessed the risk of bias in each included study following Study Quality Assessment Tools from National Institute of Health - National Heart, Lung, and Blood Institute (NIH-NHLBI) which consisted of 12–14 criteria depending on the study design [61], and then this determination was reviewed by either HK or LMB. Any disagreement was resolved within the team. The quality assessment primarily focused on the risk of bias stemming from study design, sampling methods, participant recruitment, sample size, data collection, disability measurement, and data analysis including adjustment for confounding.

Studies were categorized based on their risk of bias: (1) low risk of bias if all or almost all criteria were met and unmet criteria were considered to be unlikely to alter the study results and conclusions; (2) medium risk if some of the criteria were met, and unmet criteria were considered to be unlikely to alter the study results and conclusions; (3) high risk if only a few or no criteria were met, and unmet criteria were considered potentially altering the results and conclusions. Any reviewer disagreements were discussed and resolved to reach a consensus.

### Data analysis

A meta-analysis was not feasible, due to significant heterogeneity in the measurement approaches and results across studies. Consequently, we employed a narrative synthesis approach, comparing access to health insurance among people with disabilities, and in comparison, to people without disabilities. We also explored the association of health insurance with healthcare utilization, financial protection, and health status/outcomes amongst (1) insured people with disabilities compared to insured people without disability, and (2) insured compared to uninsured people with disabilities.

Healthcare utilization was differentiated into general healthcare (e.g., vaccination, sexual and reproductive health) and disability-related healthcare (e.g., specialist care related to impairments, rehabilitation, assistive technology services). Financial protection indicators included out-of-pocket payment (OOP), catastrophic health expenditure (CHE), and poverty. Health status/outcome was measured by self-rated health, quality of life, and health-related quality of life.

Countries were categorized based on the World Health Organization (WHO) region i.e., Africa, Southeast Asia, West Pacific, Eastern Mediterranean, and Pan America. The classification of country's income followed the World Bank Classification (2022), including low-income, lower-middle-income, and upper-middle-income. Health insurance was grouped into (1) public, any, or unclear (e.g., national health insurance, social health insurance, other government-managed health insurance, any health insurance, any health insurance or no information), and (2) private (e.g., private health insurance and other non-government-managed health insurance).

### Reflexivity

The review was conducted by LA, a person with disabilities from Indonesia with a background in medicine and public health, alongside HK and LMB, both of whom have extensive experience in disability research and are based in the UK. HK, a professor of epidemiology, has significant expertise in global health and disability research, while LMB, an associate professor, specializes in disability and social protection.

## Results

### Search results

**Fig. 1:**
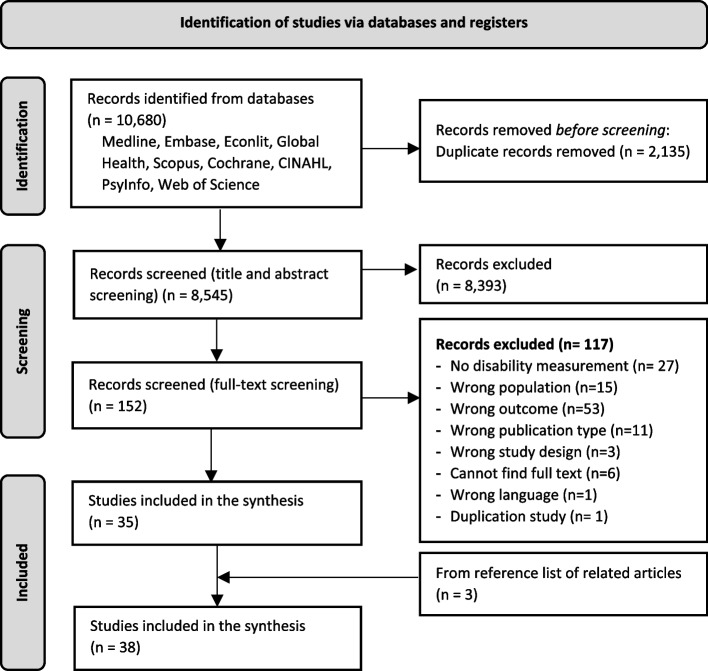
PRISMA flowchart of study selection process

We identified 10,680 records across the search of nine databases, of which 2,135 were removed due to duplication. A further 8,393 records were excluded after title and abstract screening, and 152 records went to the full-text screening phase. We excluded 117 records that did not meet the inclusion criteria, of which 6 were due to the unavailability of the full text even after reaching out to the authors. Three eligible articles were identified from the references of related articles and were included in the review. Finally, thirty-eight records were included in the synthesis (Fig. 1).

### Description of the studies

All included studies were published from the year 2000 onwards. This review synthesized findings of studies from 14 specific countries (35/38) and 3 multi-countries studies [61–63], and all of the studies covered data from 51 countries. Around one-third of the studies were from China (31.6%), five from Vietnam (13.2%), four from Ghana (10.5%), smaller proportions from other countries, and three covered multiple countries (7.9%).

When categorized by the World Bank Income Classification (2022), half of the studies were from upper-middle-income countries (50.0%), one-third were from lower-middle-income countries (34.2%), and few were from low-income countries (7.9%), and multiple countries (7.9%) (Table 1). In terms of study design, the majority were cross-sectional (89.5%). Half of studies (50.0%) focused on any type of disability, while others looked at specific impairments such as mental (31.6%), physical (18.4), and vision (7.9%). Regarding health insurance types, the majority were in the category of public, or any health insurance (92.1%). Only four (10.5%) studies looked at schemes that explicitly target people with disabilities. Study outcomes varied, with more than two-third presenting health insurance coverage (68.4%), followed by the assessment of healthcare utilization (44.7%). A smaller proportion examined financial protection (15.8%) and health status (10.5%) (Table 1).

**Table 1:** Characteristics of the Included Studies (N: 38)

Characteristics	n (%)
Countries	
Burkina Faso	1 (2.6)
Ghana	4 (10.5)
Rwanda	1 (2.6)
Argentina	1 (2.6)
Brazil	2 (5.3)
Mexico	1 (2.6)
Peru	2 (5.3)
Colombia	1 (2.6)
India	1 (2.6)
Nepal	1 (2.6)
Iran	2 (5.3)
Sudan	1 (2.6)
China	12 (31.6)
Vietnam	5 (13.2)
Multi countries	3 (7.9)
WHO Region	
Africa	6 (15.8)
Pan America	8 (21.1)
South-East Asia	2 (5.3)
Eastern Mediterranean	3 (7.9)
Western Pacific	17 (44.7)
Multi region	2 (5.3)
World Bank Classification by income countries (2022)	
Low income	3 (7.9)
Lower-middle income	13 (34.2)
Upper-middle income	19 (50.0)
Multi-countries	3 (7.9)
Type of disabilities^#^	
Physical	7 (18.4)
Vision (sensory)	3 (7.9)
Hearing (sensory)	0 (0.0)
Mental	12 (31.6)
Intellectual	0 (0.0)
Any type	19 (50.0)
Type of health insurance	
Public, any or unclear	35 (92.1)
Private	3 (7.9)
Study outcome*	
Coverage	26 (68.4)
Healthcare utilization	17 (44.7)
Financial protection	6 (15.8)
Health status	4 (10.5)
Risk of bias assessment	
Low	15 (39.5)
Medium	12 (31.5)
High	11 (29.0)

^#^A study may have more than one type of disability measure

*A study may have more than one outcome

In terms of quality assessment, more than one-third of the studies (39.5%) had a low risk of bias, while twelve studies (31.5%) were categorized as having a medium risk of bias, and the rest (29.0%) were at high risk of bias (Table 1). The primary contributors to potential bias stemmed from the sampling methods, particularly the reliance on convenience sampling, and the recruitment strategy, including facility-based, registry-based, and claim-based approaches. These aspects constrained the generalizability of the findings since the estimate was not reflective of the broader population. Additionally, in the analysis, controlling for socioeconomic status, income, and wealth, raised concerns of over-adjustment, as these factors might lie within the causal pathway between disability, health insurance, and outcomes of interest.

### Subgroup analysis

Five studies assessed coverage amongst people with disabilities by disaggregating findings based on various types of impairments or disabilities, though their categories and measurements differed. Atchesi (2014) reported coverage by vision and mobility impairments, while Gomez (2021) focused on mobility impairments, ADL (Activities of Daily Living), and IADL (Instrumental Activities of Daily Living) status. Lund (2019) examined coverage across mental, neurological, and substance-use disorders, Huang (2013) categorized coverage by severity level, and Doubova (2015) reported coverage across different types of difficulties. Three studies disaggregated coverage by gender—Huang (2013), Flores-Flores (2018), and Wiredu (2021)—while El Sayed (2015) assessed healthcare utilization by gender. For gender 1/3 found women had higher coverage [62], 1/3 was not a comparison study [63], 1/3 found lower coverage [64] (Additional file 2).

### Health insurance coverage

Among the twenty-six studies examining health insurance coverage, sixteen (61.5%) compared coverage between people with and without disability [27, 64–78], while ten studies (38.5%) presented coverage among people with disabilities without a comparison [62, 63, 79–86]. Two studies focused on private health insurance, and two studies reported coverage of health insurance that explicitly targeted people with disabilities [27, 73] (Additional file 2).

Among comparison studies (*n* = 16), five studies from Vietnam (*n* = 2), China, Burkina Faso, and Colombia showed a positive association, meaning that the coverage was higher in people with disabilities or people with disabilities were more likely to have health insurance than those without disabilities [66, 27, 70, 73, 77]. Meanwhile, a negative association was observed in another five studies conducted in Ghana, Brazil, Peru, and India, and in a pooled estimate of multiple countries (Ethiopia, India, Nepal, Nigeria, South Africa, and Uganda) [64, 65, 67, 72, 75]. Four studies from Peru, China, Rwanda and Ghana found a null association, meaning there is no significant difference in coverage between people with and without disability [68, 71, 76]. Two studies from Mexico and Vietnam reported mixed findings (positive and negative associations from several comparisons within a study) [69, 74] (Additional file 2).

Among studies reporting health insurance coverage, the mean of health insurance coverage among people with disabilities across 16 population-based studies were 41.4% [27, 63, 64, 67–69, 73–75, 77, 79–84], with the highest coverage found in Vietnam (96%—any health insurance) [27] and the lowest in China (5.1%—medical insurance) [80]. Even though the relative coverage and probability of having health insurance among people with disabilities was inconclusive, overall, health insurance coverage among people with disabilities remained low.

### Health insurance and healthcare utilization

Seventeen studies investigated the relationship between health insurance and healthcare utilization among people with disabilities. Five studies evaluated the utilization of general healthcare (5/17) [74, 82, 87–89], and twelve assessed disability-related healthcare (12/17) (Table 2, Additional file 3) [79–81, 83, 85, 86, 90–95]. All studies compared insured and uninsured people with disabilities, among them two studies reported a comparison of more to less generous health insurance schemes [86, 95].

**Table 2:** Association between health insurance and healthcare utilization amongst people with disabilities in LMICs

	**Studies**	**Country**	**Health Insurance**	**Disability (measurement)**	**Healthcare utilization (recall period)**	**Results (insured vs uninsured)**	**Association (insured vs uninsured)**	**Risk of bias**
General healthcare utilization	Chen & Ning (2022) [96]	China	Long-term care insurance, Public	All types (Barthel Index – ADL function)	1. Outpatient visit (last month) 2. Number of hospitalizations (last year) 3. Inpatient length of stay (last year)	1. Reduced by 0.322 times (*P* < 0.01) 2. Reduced by 0.158 times (*P* < 0.01) 3. Reduced by 1.441 days (*P* < 0.01)	Negative	Low
	Mai (2022) [82]	China	Any health insurance	All types (ADL)	Access to healthcare services (unclear)	OR 1.2 (0.73–1.97)	Null	Low
	Palmer (2012) [74]	Vietnam	Compulsory health insurance, Public	All types (self-reported yes/no)	1. Public inpatient services (12 months) 2. Public outpatient services (1 month)	Regression coefficient 1. -0.002 SE 0.011 *P* > 0.1 2. 0.045 SE 0.014 *P* < 0.01	Positive	Medium
	Palmer (2014) [88]	Vietnam	Social health insurance, Public	All types (Washington Group Short Set)	1. Inpatient (last 12 months) 2. Outpatient (last 1 month) 3. Self-treatment (last 1 month)	Results from PSM 1. 0.093 (*P* < 0.01) 2. 0.034 (*P* > 0.1) 3. 0.033 (*P* > 0.1) Covariate matching 1. 0.111 (*P* < 0.01) 2. 0.107 (*P* < 0.01) 3. -0.042 (*P* < 0.01)	Mixed	Low
	Shiwakoti et al. (2021) [89]	Ilam District, Nepal	Any health insurance, no informatio	All types (Washington Group Short Set)	Utilization of sexual and reproductive health services (unclear)	Crude OR: 1.2 (0.60 – 2.31)	Null	High
	**Studies**	**Country**	**Health Insurance**	**Disability (measurement)**	**Healthcare utilization (recall period)**	**Results (insured vs uninsured)**	**Association (insured vs uninsured)**	
Disability-related healthcare utilization	Contentti (2019)	12 Latin America countries	Any health insurance	Physical -Multiple sclerosis (clinical diagnosis)	1. Disease-Modifying Therapy (DMT) past 12 months 2. MRI for diagnosis (lifetime) 3. Evoked Potential (lifetime) 4. Lumbar puncture (lifetime) 5. Rehabilitation (unclear)	Uninsured vs insured 1. 90.9% vs 85.3%, P: 0.90 2. 98.7% vs 98.7%, P:1 3. 73.7% vs 76.6%, P: 0.42 4. 106 (67.9) vs 898 (68.3), P: 0.92 5. 35 (22.4%) vs 239 (18.2%), P: 0.19	Null	Low
	El Sayed (2015)	48 LMICs	Any health insurance	Mental (self-reported diagnosis)	Treatment uptake of persons with schizophrenia or depression (lifetime)	Uninsured vs insured Schizophrenia 1. Male: 0.75 (0.51–1.11) 2. Female: 0.57 (0.47–0.69) Depression 1. Male: 0.59 (0.37–0.92) 2. Female: 0.93 (0.80–1.08)	Positive	Low
	Fan (2022)	China	Long-term care insurance, Public	All types (ADL, disability defined > 1 limitation)	**Unmet** LTCI needs (unclear)	PSM- Difference-in-Difference (DiD) coefficient: -0.107, SE: 0.05, *P* < 0.05	Positive	Low
	Guo (2015) [80]	China	Any health insurance	All types (physician diagnosis using ICD-10, ICF, WHO-DAS)	Healthcare utilization: curative care including surgeries and pharmaceutical treatments, auxiliary aids including assistive devices and services, and rehabilitation (unclear)	Uninsured vs insured OR 0.80 (0.74–0.87)	Positive	Medium
	Guo (2017) [81]	China	Medical insurance, Any health insurance	Mental (clinical diagnosis based on ICF, and ICD-10)	Mental health service utilization (lifetime)	AOR: 1.45 (1.21–1.72), *P* < 0.001	Positive	Medium
	Li (2013) [92]	China	Medical insurance, Any health insurance	Mental (clinical diagnosis using ICD-10 and WHO-DAS 2)	1. Any mental health services 2. Medical-pharmaceutical (unclear) 3. Rehabilitation (unclear) 4. Medical and rehabilitation (unclear)	AOR 1. 1.39 (1.24–1.55) 2. 1.39 (1.24–1.56) 3. 1.10 (0.67–1.81) 4. 1.38 (1.12–1.70)	Positive	Medium
	Machnicki (2011) [93]	Argentina	Private health insurance, Private	Mental – depression (clinical diagnosis, DSM IV and ICD-10)	Not receiving depression treatment – antidepressant (unclear)	Uninsured vs insured AOR: 7.12 (1.88 – 26.86)	Positive	High
	Medeiros (2021) [83]	Brazil	Any health insurance	All types (Self-reported yes/no)	Experience of attending at least one rehabilitation service (lifetime)	APR: 1.31 (1.15–1.49) *P* < 0.001	Positive	Medium
	Nartey (2018)	Ghana	Social Health Insurance, Public	Mental (clinical diagnosis)	The use of mental health services as initial point of care – biomedical vs faith-based (unclear)	AOR 2.47 (0.60 – 10.11) P: 0.20	Null	High
	Nattaj (2017) [94]	Iran	Public	Mental (clinical diagnosis)	Length of hospitalization – days staying at hospital (lifetime)	RR: 0.71 (0.59 – 0.84) *P* < 0.001	Negative	High
	Shi (2019) [86]	China	Urban social insurance for workers, Public	Mental—(clinical diagnosis based on ICD-10)	Mental health service utilization (2013–2016) Self-pay vs urban social insurance for workers 1. Community health centre vs specialty health centre 2. Secondary vs specialty hospital 3. Tertiary vs specialty hospital	uninsured vs insured AOR (95% CI) 1. 29.49 (16.16 – 53.81) *P* < 0.0001 2. 3.49 (2.46 – 4.95) *P* < 0.0001 3. 9.82 (7.04 – 13.71) *P* < 0.0001	Positive	Medium
	Shi (2019) [86]	China	Urban social insurance for citizens, Public	Mental—(clinical diagnosis based on ICD-10)	Mental health service utilization (2013–2016) 1. Community health centre vs **specialty health centre** 2. Secondary vs **specialty hospital** 3. Tertiary vs **specialty hospital**	Urban social insurance for citizens vs urban social insurance for workers (more generous) 1. 4.01 (1.99 – 8.07) *P* < 0.0001 2. 0.99 (0.59 – 1.68) P: 0.992 3. 3.14 (1.83 – 5.37) *P* < 0.0001	Positive	Medium
	Zhang (2018) [95]	China	Urban Employee Basic Medical Insurance (UEBMI), Public	Mental – schizophrenia (clinical diagnosis based on ICD-10)	Mental health service use based on claim 2010–2014 1. Total cost of service use 2. Inpatient 3. Outpatient Measured at baseline, first, second and third year follow up	UEBMI (more generous) vs URBMI: Baseline 1. 42,543.1 vs 41,143.0 P: 0.021 2. 42,375.1 vs 40,917.3 P: 0.018 3. 168.0 vs 225.7 P: 0.031 Third year 1. 60,163.7 vs 51,875.6 *P* < 0.001 2. 60,145.2 vs 51,804.5 P < 0.001 3. 18.4 vs 71.1 P: 0.069	Positive	Low

*Abbreviations: APR* Adjusted Prevalence Ratio, *AOR* Adjusted Odds Ratio, *RR* Risk Ratio, *DiD* Difference-in-difference, *PSM* Propensity Score Matching

Positive: Among people with disabilities, the insured have higher healthcare utilization than those uninsured. Mixed results of positive and null are categorized as positive

Negative: Among people with disabilities, the insured have lower healthcare utilization than those uninsured. Mixed results of negative and null are categorized as negative

Null: There is no difference in healthcare utilization between the insured and uninsured people with disabilities

Mixed: There is more than one measure showing positive and negative associations

Five studies reported the association between health insurance and general healthcare use. Of these studies, three were classified as having a low risk of bias [82, 88, 96], while the remaining two had a high [89] and medium risk of bias [74], respectively. Three studies assessed inpatient and outpatient utilization as the indicator [74, 88, 96], a study examined access to healthcare [82] and a study evaluated sexual and reproductive services use among women with disabilities [89]. One study from Vietnam reported a positive association [74], meaning that health insurance was associated with increased healthcare use among people with disabilities. A study from China showed a negative association [96]. Two studies from China and Nepal reported a null association [82, 89], and a study from Vietnam demonstrated mixed associations (positive and negative associations from different comparisons and analysis) [88] (Table 2, Additional file 3). Overall, the findings on general healthcare use varied and were inconclusive.

Twelve studies evaluated association between health insurance and disability-related healthcare utilization. Of these twelve studies, four were categorised as low risk of bias [79, 90, 91, 95], five with medium [80, 81, 83, 86, 92] and three with high risk of bias [85, 93, 94]. Associations were positive for nine studies (China (*n* = 6), Argentina, Brazil, and 48 LMICs) [80, 81, 83, 86, 90–93, 95], negative for one study from Iran [94], and null for two studies from Ghana and 12 Latin America [79, 85] (Table 2). There were no clear differences in pattern by type of disability-related care (e.g., rehabilitation, assistive technology, treatment) (Additional File 3).

Two studies from China that compared less to more generous health insurance (Urban Social Insurance for Citizens vs Urban Social Insurance for Workers, Urban Employee Basic Medical Insurance vs Urban Resident Basic Medical Insurance) found that more generous health insurance was associated with higher use of more advanced healthcare services (e.g., community health centre vs specialty health centre) [86], and higher utilization of specialist care [95].

Overall, health insurance appeared to be associated with the increased uptake of disability-related services by people with disabilities, while the findings on general healthcare utilization remained inconclusive.

### Health insurance and financial protection

Six studies examined the association between health insurance and financial protection with the indicators of OOP (4/6) [74, 88, 95, 96], CHE (3/6) [84, 88, 97], and poverty (1/6) [88] (Table 3, Additional file 4). Of these studies, four were categorised as good quality with low risk of bias [84, 88, 95, 96], one medium [74], and one with high risk of bias [97]. Five studies compared insured and uninsured people with disabilities and one study compared more and less generous health insurance [95].

**Table 3:** Association between health insurance and out-of-pocket payment, and catastrophic health expenditure

Studies	Country	Health insurance	Disability (measurement)	Outcome measure	Results (insured versus uninsured)	Association (insured vs uninsured)	Risk of bias
Chen & Ning (2022) [96]	China	Long-term care insurance, Public	All types (Barthel Index – ADL function)	1. OOP on outpatient 2. OOP on hospitalization 3. Total of OOP on outpatient and hospitalization	Coefficient (DiD & PSM) 1. 49.589 (*P* > 0.1) 2. -533.465 (*P* < 0.05) 3. -512.562 (*P* < 0.05)	Negative	Low
Guan (2019) [97]	China	Any insurance, public	Vision impairment (clinical diagnosis, moderate VI or worse in both eyes; VA < 6/18)	Catastrophic health expenditure percentage among (30% threshold)	No insurance: 50% New Cooperative Medical Scheme: 47.9% Urban Resident Basic Medical Insurance (URBMI): 25% Urban Employee Basic Medical Insurance (UEBMI): 30.9% Government Medical Insurance: 16.7% Commercial Medical Insurance: 21.4% P: 0.008	Positive	High
Moradi (2021) [84]	Iran	Any health insurance	Physical, mental (Registry of the Rehabilitation Department of the Welfare Organization)	Catastrophic health expenditure (40% threshold)	Uninsured vs insured AOR 6.51 (95% CI: 3.69 – 8.24)	Negative	Low
Palmer (2012) [74]	Vietnam	Compulsory Health Insurance, Public	All types (self-reported yes/no: mobility, hearing, speaking, learning, mental, vision – only severe included)	Expenditure on: 1. Public inpatient (12 months) 2. Public outpatient (1 month) **Insured people with disabilities vs uninsured people with disabilities**	1. Coefficient -0.067 P:0.1 2. Coefficient 0.013 *P* > 0.1	Null	Medium
Palmer (2012) [74]	Vietnam	Compulsory Health Insurance, Public	All types (self-reported yes/no: mobility, hearing, speaking, learning, mental, vision)	Expenditure on: 1. Public inpatient (12 months) 2. Public outpatient (1 month) **Insured people with disabilities vs insured people without disability**	1. 1297.919 vs 783.881 (*P* < 0.05) 2. 23.725 vs 17.230 (*P* > 0.05)	Positive	Medium
Palmer (2014) [88]	Vietnam	Social health insurance, Public	All types (Washington Group Short Set)	1. Inpatient expenditure per visit 2. Outpatient expenditure per visit 3. Self-treatment per visit 4. CHE 10% 5. CHE 20% 6. CHE 40% 7. Poverty 8. Poverty net of health payment 9. Poverty differential	PSM 1. 103.062 (*P* > 0.1) 2. -20.351 (*P* > 0.1) 3. 2.088 (*P* > 0.1) 4. -0.052 (*P* > 0.1) 5. -0.090 (*P* < 0.1) 6. -0.017 (*P* > 0.1) 7. 0.009 (*P* > 0.1) 8. 0.021 (*P* > 0.1) 9. 0.012 (*P* > 0.1) Covariate matching 1. 213.435 (*P* < 0.05) 2. 3.904 (*P* > 0.1) 3. 1.111 (*P* > 0.1) 4. -0.073 (*P* < 0.01) 5. -0.066 (*P* < 0.01) 6. 0.015 (*P* > 0.1) 7. 0.042 (*P* < 0.05) 8. 0.081 (*P* < 0.05) 9. 0.039 (*P* < 0.01)	OOP (1–3): Positive CHE 10%: Negative CHE 20%: Negative CHE 40%: null Poverty (7–9): Positive	Low
Zhang (2018) [95]	China	Urban Employee Basic Medical Insurance (UEBMI), Public	Mental – schizophrenia (clinical diagnosis based on ICD-10, F20)	OOP payment at: 1. baseline 2. 1 year follow up 3. 2 years follow up 4. 3 years follow up	UEBMI (more generous) vs URBMI 1. 12.7% vs 13.5% P: 0.021 2. 10.7% vs 14.5% *P* < 0.001 3. 10.7% vs 5.9% *P* < 0.001 4. 9.5% vs 6.1% *P* < 0.001	Mixed 1. Negative 2. Negative 3. Positive 4. Positive	Low

*Abbreviation*: *PSM* Propensity Score Matching, *DiD* Difference-in-Difference

Positive: Among people with disabilities, the insured have higher OOP/CHE than those uninsured. Or OOP/CHE is higher in insured people with disabilities than insured people without disability. Mixed results of positive and null are categorized as positive

Negative: Among people with disabilities, the insured have lower OOP/CHE than those uninsured. Or OOP/CHE is lower in insured people with disabilities than insured people without disability. Mixed results of negative and null are categorized as negative

Null: There is no difference in OOP/CHE between the insured and uninsured people with disabilities

Mixed: More than one measure showing positive and negative associations

Among four studies assessing OOP, a study from China reported a negative association meaning less OOP spending in the insured group [96] and two studies from Vietnam found a positive [88] and null association respectively [74]. One study from China comparing more and less generous health insurance reported mixed findings, positive and negative associations depending on the follow-up time in which the first year showed a negative association, while the 2-year and 3-year follow-ups showed a positive association [95]. Among these studies, Palmer & Nguyen (2012) had an additional outcome comparing OOP between insured people with and without disabilities [74]. The results showed that among insured, people with disabilities incurred higher OOP than those without disability [74].

Among three studies reporting CHE [84, 88, 97], one from China with a 30% CHE threshold showed a positive association [97], one from Iran with a 40% cut-off found a negative association [84], and one from Vietnam demonstrated a negative association at 10% and 20% threshold, and null at 40% level [88].

Only one study, from Vietnam, reported the impact of health insurance on poverty and showed a positive association from covariate matching analysis, meaning that health insurance was associated with an increase in poverty [88].

In summary, the relationship between health insurance and financial protection among people with disabilities was inconclusive, largely due to the lack of studies.

### Health insurance and health status/outcome

Four studies evaluated health status/outcome including self-rated health [91] and quality of life (QoL) [97–99] (Table 4). Of these, one was categorised as good quality with low risk of bias [91], and the rest possessed high risk of bias [97–99].

**Table 4:** Association between Health insurance and health status/outcomes amongst people with disabilities in LMICs

Studies	Country	Health insurance	Type of disability (measurement)	Outcome measure	Results	Association (insured vs uninsured)	Risk of bias
Fan (2023) [91]	China	Long term care insurance, Public	All types (ADL—> 1 limitation)	1. Self-rated life satisfaction 2. Self-reported health 3. Unmet LTC needs	Coefficient (PSM DiD) 1. 0.2121 (0.0671) *P* < 0.01 2. 0.1389 (0.0707) *P* < 0.05 3. -0.1071 (0.0502) *P* < 0.05	1. Positive 2. Positive 3. Positive	Low
Guan (2019) [97]	China	Any health insurance, public	Vision impairment (clinical diagnosis, moderate VI or worse in both eyes; VA < 6/18)	Quality of life (The Time Trade-Off [TTO] evaluation technique)	Quality of Life (mean) No insurance: 0.64 New Cooperative Medical Scheme: 0.63 Urban Resident Basic Medical Insurance: 0.81 Urban Employee Basic Medical Insurance: 0.63 Government Medical Insurance: 0.66 Commercial Medical Insurance: 0.49 P: 0.003	NA	High
Mohammed (2019) [98]	Sudan	Any health insurance	Physical – Cerebral Palsy (clinical diagnosis – medical record)	Quality of Life (4 domains: physical, schooling, social, emotional; low response 0, high response 1)	Mean difference of QoL (insured vs uninsured) B: 7.910; *P* = 0.018	Positive	High
Tien (2021) [99]	Vietnam	Any health insurance	Physical – complete cervical spinal cord injury (clinical diagnosis – medical record)	1. Health-related Quality of Life (HRQoL) (EuroQoL-5D-5L) 2. ADL (Katz Index) 3. IADL	Insured vs uninsured aCoef (95%CI) 1. -0.11 (-0.21—-0.006) *P* < 0.05 2. -1.63 (-2.83—-0.43) *P* < 0.01 3. 0.91 (-0.07 – 1.90)	1. Negative 2. Negative 3. Null	High

*Abbreviation*: *PSM* Propensity Score Matching, *DiD* Difference-in-Difference, *QoL* Quality of Life

Positive: Among people with disabilities, the insured had better health status than the uninsured. Mixed results of positive and null are categorized as positive

Negative: Among people with disabilities, the insured had worse health status than the uninsured. Mixed results of negative and null are categorized as negative

Null: There is no difference in health status between the insured and uninsured people with disabilities

Mixed: When a study has more than one result showing positive and negative associations

Two studies from China and Sudan showed a positive association [91, 98], meaning that insured people with disabilities had better self-rated or QoL than those uninsured, and one study from Vietnam reported a negative association [99]. A study from China only presented a Quality of Life (QoL) score in each health insurance scheme [97] in which the results showed an association between health insurance and QoL scores (P: 0.003) (Table 4). However, there was no further analysis comparing between insured and uninsured. Overall, despite the limited number of studies, health insurance appeared to be positively associated with improved health status/outcomes. However, majority of these studies raised concerns due to a high risk of bias.

## Discussion

This analysis identified 38 studies covering data from 51 countries and exploring access to health insurance and its association with healthcare use, financial protection, and health status amongst people with disabilities in LMICs. The key findings of this review highlight several notable points. Firstly, health insurance coverage amongst people with disabilities on average is low, and the findings on comparison between people with and without disabilities remain inconclusive. Secondly, health insurance exhibits a positive association with access to disability-related healthcare services, including specialist services, treatment, rehabilitation, and assistive technology. However, the relationship with general healthcare is inconclusive, primarily due to the limited number of studies available. Thirdly, the association of health insurance on financial protection also remains inconclusive, again due to limited evidence. Fourthly, the finding shows a favorable association between health insurance and health status, despite a concern on the quality of studies.

The studies included in our review predominantly focus on coverage/access. Due to the high variability of health insurance types, there exists a broad spectrum of coverage, and inconclusive findings regarding the access and coverage of health insurance for individuals with disabilities compared to those without disabilities. This aligns with a previous review highlighting the highly heterogeneous nature of insurance coverage across countries in Asia and Africa within the general population [100]. Another review covering studies from LMICs underscored that the differences in coverage could be linked to variations in health systems and/or health insurance programs, including distinct payment systems [101].

In this review, the small proportion of studies evaluating general healthcare utilization and the range of measures used yield inconclusive results. Among the general population, however, the majority of existing systematic reviews show that health insurance improved healthcare access [101, 102] and utilization [101, 103–105]. A systematic review on the role of health insurance for children with special care needs also reported the positive effects of insurance on access and healthcare utilization [53]. Our findings underscore the potential oversight in recognizing that individuals with disabilities, like their counterparts without disabilities, experience general healthcare needs. The apparent lack of emphasis on studying these needs within the context of healthcare utilization in association with health insurance among people with disabilities is noteworthy.

In the context of disability-related healthcare utilization, three-quarters of the studies reported an increased utilization associated with health insurance including rehabilitation and impairment-related services. This finding is consistent with a previous review indicating a positive association between health insurance and heightened utilization of mental health care services among individuals with mental, neurological, and substance-use (MNS) disorders in LMICs [106], and that health insurance increased the likelihood of accessing neurologists among individuals with multiple sclerosis from a survey conducted in the United States [107]. Moreover, a study conducted in the United States identified health insurance as a protective factor against the reported unmet need for therapy services and assistive devices [55]. However, a distinct result was observed in a review focusing on Long-Term Care Insurance in China, which indicated a reduction in outpatient visits and hospitalizations. This discrepancy may be attributed to the programme's broader coverage, which includes not only medical care services but also basic daily living, home care, and nursing care, potentially reinforcing the preventive and promotive aspects of healthcare [108].

Evidence on the association between health insurance and financial protection (OOP and CHE) among people with disabilities was limited and inconclusive. Literature from the general population, however, consistently demonstrates the association of health insurance with reductions in OOP [53, 101, 104, 105, 109, 110], CHE [101, 104, 110–113], and poverty [110]. In addition to that, a previous review on the role of health insurance on children with special healthcare needs shows lower OOP expenditure among those insured than the uninsured [53]. Similarly, a study from the US demonstrated that health insurance protected people with disabilities from CHE [114]. The observed discordance can be attributed to the limited number of eligible studies in this review. Furthermore, health insurance in LMICs often offers no or limited coverage of disability-related healthcare [115, 116].

One intriguing finding from a study in Vietnam is that health insurance is associated with increased poverty among people with disabilities [88]. The same study reported elevated healthcare utilization and OOP expenses among insured people with disabilities compared to those without disability. This phenomenon may be attributed to the insufficient coverage of healthcare needs by health insurance (e.g., not fully covering or not covering certain services).

Additionally, despite healthcare services being covered, individuals with disabilities often lack other forms of social protection to address indirect costs, including heightened transportation expenses, caregiver-related expenditures, and other opportunity costs and forgone needs [56, 117] which are rarely captured in a study. This may indicate that health insurance alone cannot overcome the broad range of barriers people with disabilities face in accessing healthcare.

Only four studies explore the association of health insurance with health status, with the predominant findings indicating a favourable association between health insurance and self-reported health and health-related quality of life. These observations resonate with prior reviews among the general population suggesting an association between health insurance and enhanced self-reported health [101]. Another review, focusing on individuals receiving Long-Term Care Insurance in China, similarly demonstrates an improvement in the quality of life associated with health insurance [108]. Furthermore, an additional review contributed to the literature by highlighting an association between health insurance and a reduction in mortality among the enrolled population [103]. Despite the concordance of these findings, further studies are needed to address the current gap in evidence.

This review demonstrates the evidence gap in health insurance among people with disabilities, the limited representation of studies from low-income countries, and the overwhelming concentration of the literature in China (12/38). Furthermore, most studies focused on access to health insurance, with very few exploring the association between health insurance and general healthcare utilization, financial protection, and health status/outcomes. These areas should be priorities for future research.

Our review boasts several strengths. First, it covers multiple databases, uses comprehensive search terms, and has a dual review. Secondly, this review builds upon the findings of previous reviews that highlighted the lack of strong evidence on health insurance for people with disabilities in LMICs [58, 59]. While the earlier reviews identified three studies [59] and nine studies [58] on this topic, the current review includes 38 studies. Twenty four of these 38 studies were published since 2016 (when searching for the most recent review was finished), indicating a growing interest in this area. Finally, we offer a comprehensive review by synthesizing evidence across four key outcomes: access/coverage, healthcare utilization, financial protection, and health status.

Our study has several limitations that warrant consideration. Importantly, we were only able to review English-language articles, introducing the potential for publication and language bias. Additionally, this review may have excluded relevant studies written in other languages, such as those in Latin America, where social protection systems have been rapidly evolving in recent years.

The majority of the included studies employed a cross-sectional design, making it challenging to establish causal relationships. In addition, the wide range of indicators and measurements used across studies poses challenges in conducting meaningful comparisons and drawing aggregate conclusions. Finally, we were unable to perform subgroup analyses based on gender, type of disability, type of health insurance, and specific healthcare utilization categories (e.g., inpatient and outpatient services) as few studies disaggregated data amongst people with disabilities. Further research is needed to understand if coverage and health outcomes tied to insurance vary by these factors.

## Conclusion

Our study highlights the complex relationship between health insurance and the well-being of individuals with disabilities. The geographical skew, with most studies from China and upper-middle-income countries, emphasizes the need for research in other contexts. While our findings indicate limited health insurance coverage among people with disabilities, they consistently show a positive association between health insurance and disability-related healthcare utilization. However, evidence on the relationship between health insurance and general healthcare utilization, as well as financial protection, remains limited and inconclusive, necessitating further targeted investigations. Despite these gaps, our study suggests that health insurance may improve health status/outcomes for individuals with disabilities, reinforcing the need for diverse research to inform effective policies aimed at achieving Universal Health Coverage. Future studies should incorporate subgroup analyses that consider gender, types of disabilities, and other intersecting characteristics.

## Supplementary Information

Additional file 1. Search strategies.

Additional file 2. Health insurance coverage/access among people with disabilities in LMICs.

Additional file 3. Association between health insurance and healthcare utilization amongst people with disabilities in LMICs.

Additional file 4. Association between health insurance and Out-of-pocket Payment (OOP), Catastrophic Health Expenditure (CHE) amongst people with disabilities in LMICs.

## Data Availability

No datasets were generated or analysed during the current study.

## Abbreviations

ADL: Activities of Daily Living
CHE: Catastrophic Health Expenditure
IADL: Instrumental Activities of Daily Living
LMICs: Low- and Middle-Income Countries
MNS: Mental, Neurological, and Substance-use disorders
OOP: Out-of-Pocket Payment
QoL: Quality of Life
UHC: Universal Health Coverage
WHO: World Health Organization

## References

[1] United Nations Convention on the Rights of Persons with Disabilities. 2006. Available from: http://www.un.org/esa/socdev/enable/rights/convtexte.htm. 10.1515/9783110208856.203 18348362

[2] World Health Organization. Disability. 7 March 2023. 2023. Available from: https://www.who.int/health-topics/disability#tab=tab_1. Cited 2023 Apr 25.

[3] World Health Organization. Global report on health equity for persons with disabilities. Geneva: World Health Organization; 2022. Licence: CC BY-NC-SA 3.0 IGO.

[4] Banks LM, Kuper H, Polack S. Poverty and disability in low-and middle income countries: A systematic review. PLoS One: Public Library of Science. 2017;12(12):e0189996. 10.1371/journal.pone.0189996. Erratum in: PLoS One. 2018;13(9):e0204881. 10.1371/journal.pone.0204881. PMID: 29267388; PMCID: PMC5739437. 10.1371/journal.pone.0189996 PMC5739437 29267388

[5] World Health Organization. World Bank. World report on disability. Geneva: World Health Organization; 2011. Available from: https://www.ncbi.nlm.nih.gov/books/NBK304079/.

[6] Bright T, Kuper H. A systematic review of access to general healthcare services for people with disabilities in low and middle income countries. MDPI AG: Int J Environ Res Public Health; 2018. 10.3390/ijerph15091879 PMC6164773 30200250

[7] Kuper H, Heydt P. The Missing Billion: Access to Health Services for 1 Billion People with Disabilities. PLoS One: Public Library of Science; 2019.

[8] Banks LM, Hameed S, Abu Alghaib O, Nyariki E, Olenja J, Kulsum U, et al. “It is too much for us”: direct and indirect costs of disability amongst working-aged people with disabilities in Dhaka, Bangladesh and Nairobi Kenya. J Human Dev Capabil. 2022;23:228–51.

[9] Gudlavalleti VSM. Challenges in Accessing Health Care for People with Disability in the South Asian Context: A Review. International Journal of Environmental Research and Public Health 2018, Vol 15, Page 2366. 2018;15:2366. Available from: https://www.mdpi.com/1660-4601/15/11/2366/htm. Cited 2023 Feb 22. 10.3390/ijerph15112366 PMC6265903 30373102

[10] Mutwali R, Ross E. Disparities in physical access and healthcare utilization among adults with and without disabilities in South Africa. Disabil Health J. 2019;12:35–42. Available from: https://pubmed.ncbi.nlm.nih.gov/30082200/. Cited 2023 Feb 28. 10.1016/j.dhjo.2018.07.009 30082200

[11] Moodley J, Ross E. Inequities in health outcomes and access to health care in South Africa: a comparison between persons with and without disabilities. 2015;30:630–44. Available from: 10.1080/09687599.2015.1034846. https://www.tandfonline.com/doi/abs/10.1080/09687599.2015.1034846. Cited 2023 Feb 28.

[12] Crewe JM, Spilsbury K, Morlet N, Morgan WH, Mukhtar A, Clark A, et al. Health Service Use and Mortality of the Elderly Blind. Ophthalmology. 2015;122:2344–50. Available from: http://www.aaojournal.org/article/S0161642015006673/fulltext. Cited 2022 Dec 15. 10.1016/j.ophtha.2015.07.001 26394754

[13] Hanson KW, Neuman P, Dutwin D, Kasper JD. Uncovering the health challenges facing people with disabilities: the role of health insurance. Health Aff (Millwood). 2003;Suppl Web Exclusives. Available from: https://pubmed.ncbi.nlm.nih.gov/15506159/. Cited 2023 Mar 10. 10.1377/hlthaff.w3.552 15506159

[14] Adugna MB, Nabbouh F, Shehata S, Ghahari S. Barriers and facilitators to healthcare access for children with disabilities in low and middle income sub-Saharan African countries: a scoping review. BMC Health Serv Res. 2020;20:15. 31906927 10.1186/s12913-019-4822-6 PMC6945633

[15] Soltani S, Takian A, Akbari Sari A, Majdzadeh R, Kamali M. Financial Barriers to Access to Health Services for Adult People with Disability in Iran: The Challenges for Universal Health Coverage. Iran J Public Health. 2019. Available from: http://ijph.tums.ac.ir. PMC6570792 31223579

[16] Matin BK, Williamson HJ, Karyani AK, Rezaei S, Soofi M, Soltani S. Barriers in access to healthcare for women with disabilities: a systematic review in qualitative studies. BMC Womens Health. 2021;21:44. 33516225 10.1186/s12905-021-01189-5 PMC7847569

[17] Matin BK, Kamali M, Williamson HJ, Moradi F, Solatni S. The predictors of access to health services for people with disabilities: A cross sectional study in Iranian context. Med J Islam Repub Iran. 2019;33:1–7. Available from: https://pubmed.ncbi.nlm.nih.gov/32280631/. Cited 2023 Feb 28. 10.34171/mjiri.33.125 PMC7137830 32280631

[18] Iseselo MK, Ambikile JS. Medication challenges for patients with severe mental illness: experience and views of patients, caregivers and mental health care workers in Dar es Salaam, Tanzania. Int J Ment Health Syst. 2017;11. Available from: https://pubmed.ncbi.nlm.nih.gov/28184242/. Cited 2023 Feb 28. 10.1186/s13033-017-0126-6 PMC5294727 28184242

[19] Reichard A, Stransky M, Phillips K, McClain M, Drum C. Prevalence and reasons for delaying and foregoing necessary care by the presence and type of disability among working-age adults. Disabil Health J. 2017;10:39–47. Available from: https://pubmed.ncbi.nlm.nih.gov/27771217/. Cited 2023 Mar 10. 10.1016/j.dhjo.2016.08.001 27771217

[20] Asa GA, Fauk NK, Ward PR, Mwanri L. The psychosocial and economic impacts on female caregivers and families caring for children with a disability in Belu District Indonesia. PLoS One. 2020;15:1–16. 10.1371/journal.pone.0240921. 10.1371/journal.pone.0240921 PMC7641436 33147246

[21] Pinilla-Roncancio M. Disability and poverty: two related conditions. A review of the literature. Revista Facultad de Medicina. 2015;63:113–23.

[22] Bales S. Impact of health shocks on household welfare in Vietnam-Estimates using fixed effects estimation. Rotterdam: Erasmus University Rotterdam, Institute of Health Policy & Management; 2013.

[23] Simeu N, Mitra S. Disability and household economic wellbeing: evidence from Indonesian longitudinal data. Oxf Dev Stud. 2019;47:275–88.

[24] Flores-Flores O, Bell R, Reynolds R, Bernabé-Ortiz A. Older adults with disability in extreme poverty in Peru: How is their access to health care? PLoS One. 2018;13. Available from: https://pubmed.ncbi.nlm.nih.gov/30586426/. Cited 2023 Feb 28. 10.1371/journal.pone.0208441 PMC6306199 30586426

[25] Banks LM, Kuper H, Polack S. Poverty and disability in low-And middleincome countries: a systematic review. PLoS ONE. 2017;12:1–19. 10.1371/journal.pone.0189996 PMC5739437 29267388

[26] Banks LM, Hameed S, Usman SK, Kuper H. No one left behind? Comparing poverty and deprivation between people with and without disabilities in the maldives. Sustainability (Switzerland). 2020;12:2026.

[27] Banks LM, Walsham M, van Minh H, Duong DTT, Ngan TT, Mai VQ, et al. Access to social protection among people with disabilities: evidence from Viet Nam. Int Soc Secur Rev. 2019;72:59–82.

[28] Banks LM, Pinilla-Roncancio M, Walsham M, Van Minh H, Neupane S, Mai VQ, et al. Does disability increase the risk of poverty ‘in all its forms’? Comparing monetary and multidimensional poverty in Vietnam and Nepal. Oxf Dev Stud. 2021;49:386–400.

[29] Mitra S, Posarac A, Vick B. Disability and poverty in developing countries: a multidimensional study. World Dev. 2013;41:1–18.

[30] Guets W, Behera DK. Does disability increase households’ health financial risk: evidence from the Uganda demographic and health survey. Glob Health Res Policy. 2022;7:2. 34983699 10.1186/s41256-021-00235-x PMC8728967

[31] Palmer M, Groce N, Mont D, Nguyen OH, Mitra S. The economic lives of people with disabilities in Vietnam. PLoS One. 2015;10:e0133623. 26197034 10.1371/journal.pone.0133623 PMC4510056

[32] Hailemichael Y, Hailemariam D, Tirfessa K, Docrat S, Alem A, Medhin G, et al. Catastrophic out-of-pocket payments for households of people with severe mental disorder: a comparative study in rural Ethiopia. Int J Ment Health Syst. 2019;13:39. 31164919 10.1186/s13033-019-0294-7 PMC6544918

[33] Barnes K, Mukherji A, Mullen P, Sood N. Financial risk protection from social health insurance. J Health Econ. 2017;55:14–29. 28619488 10.1016/j.jhealeco.2017.06.002

[34] Thuong NTT, Huy TQ, Tai DA, Kien TN. Impact of health insurance on health care utilisation and out-of-pocket health expenditure in Vietnam. Biomed Res Int. 2020;2020:9065287. 32908923 10.1155/2020/9065287 PMC7471796

[35] Jung J, Streeter JL. Does health insurance decrease health expenditure risk in developing countries? The case of China. South Econ J. 2015;82:361–84. Available from: https://onlinelibrary.wiley.com/doi/full/10.1002/soej.12101. Cited 2023 Mar 13.

[36] Alkenbrack S, Lindelow M. The Impact of Community-Based Health Insurance on Utilization and Out-of-Pocket Expenditures in Lao People’s Democratic Republic. Health Econ. 2015;24:379–99. Available from: https://onlinelibrary.wiley.com/doi/full/10.1002/hec.3023. Cited 2023 Mar 13. 10.1002/hec.3023 25784572

[37] Aji B, De Allegri M, Souares A, Sauerborn R. The Impact of Health Insurance Programs on Out-of-Pocket Expenditures in Indonesia: An Increase or a Decrease? International Journal of Environmental Research and Public Health 2013, Vol 10, Pages 2995–3013. 2013;10:2995–3013. Available from: https://www.mdpi.com/1660-4601/10/7/2995/htm. Cited 2023 Mar 11. 10.3390/ijerph10072995 PMC3734472 23873263

[38] Sarkodie AO. Effect of the National Health Insurance Scheme on Healthcare Utilization and Out-of-Pocket Payment: Evidence from GLSS 7. Humanities and Social Sciences Communications 2021 8:1. 2021;8:1–10. Available from: https://www.nature.com/articles/s41599-021-00984-7. Cited 2023 Mar 11.

[39] Okoroh J, Essoun S, Seddoh A, Harris H, Weissman JS, Dsane-Selby L, et al. Evaluating the impact of the national health insurance scheme of Ghana on out of pocket expenditures: A systematic review. BMC Health Serv Res. 2018;18:1–14. Available from: https://link.springer.com/articles/10.1186/s12913-018-3249-9. Cited 2023 Mar 11. 10.1186/s12913-018-3249-9 PMC5992790 29879978

[40] Sekyi S, Domanban PB. The Effects of Health Insurance on Outpatient Utilization and Healthcare Expenditure in Ghana. Int J Humanit Soc Sci. 2012. Available from: www.ijhssnet.com.

[41] Jütting JP. Do community-based health insurance schemes improve poor people’s access to health care? Evidence from rural Senegal. World Dev. 2004;32:273–88.

[42] Mebratie AD, Sparrow R, Yilma Z, Abebaw D, Alemu G, Bedi AS. The impact of Ethiopia’s pilot community based health insurance scheme on healthcare utilization and cost of care. Soc Sci Med. 2019;220:112–9. 30419495 10.1016/j.socscimed.2018.11.003

[43] Yilma Z, Mebratie A, Sparrow R, Dekker M, Alemu G, Bedi AS. Impact of Ethiopia’s Community Based Health Insurance on Household Economic Welfare. World Bank Econ Rev. 2015;29:S164–73. Available from: https://academic.oup.com/wber/article/29/suppl_1/S164/1686924. Cited 2023 Mar 11.

[44] Parmar D, Reinhold S, Souares A, Savadogo G, Sauerborn R. Does Community-Based Health Insurance Protect Household Assets? Evidence from Rural Africa. Health Serv Res. 2012;47:819–39. Available from: https://onlinelibrary.wiley.com/doi/full/10.1111/j.1475-6773.2011.01321.x. Cited 2023 Mar 15. 10.1111/j.1475-6773.2011.01321.x PMC3419891 22091950

[45] Habib SS, Perveen S, Khuwaja HMA. The role of micro health insurance in providing financial risk protection in developing countries- a systematic review. BMC Public Health. 2016;16:1–24. Available from: https://link.springer.com/articles/10.1186/s12889-016-2937-9. Cited 2023 Mar 11. 10.1186/s12889-016-2937-9 PMC4802630 27004824

[46] Mao W, Zhang Y, Xu L, Miao Z, Dong D, Tang S. Does health insurance impact health service utilization among older adults in urban China? A nationwide cross-sectional study. BMC Health Serv Res. 2020;20:1–9. Available from: https://link.springer.com/articles/10.1186/s12913-020-05489-8. Cited 2023 Mar 13. 10.1186/s12913-020-05489-8 PMC7346393 32646423

[47] Cheng L, Liu H, Zhang Y, Shen K, Zeng Y. The Impact of Health Insurance on Health Outcomes and Spending of the Elderly: Evidence from China’s New Cooperative Medical Scheme. Health Econ. 2015;24:672–91. Available from: https://onlinelibrary.wiley.com/doi/full/10.1002/hec.3053. Cited 2023 Mar 11. 10.1002/hec.3053 PMC4790431 24777657

[48] Erlangga D, Suhrcke M, Ali S, Bloor K. The impact of public health insurance on health care utilisation, financial protection and health status in low- And middle-income countries: A systematic review. PLoS One: Public Library of Science; 2019. 10.1371/journal.pone.0219731 PMC6713352 31461458

[49] Sparrow R, Suryahadi A, Widyanti W. Social health insurance for the poor: Targeting and impact of Indonesia’s Askeskin programme. Soc Sci Med. 2013;96:264–71. 23121857 10.1016/j.socscimed.2012.09.043

[50] Nshakira-Rukundo E, Mussa EC, Gerber N, von Braun J. Impact of voluntary community-based health insurance on child stunting: evidence from rural Uganda. Soc Sci Med. 2020;245:12738. 10.1016/j.socscimed.2019.112738 31855728

[51] Lee YC, Huang YT, Tsai YW, Huang SM, Kuo KN, McKee M, et al. The impact of universal National Health Insurance on population health: The experience of Taiwan. BMC Health Serv Res. 2010;10:1–8. Available from: https://bmchealthservres.biomedcentral.com/articles/10.1186/1472-6963-10-225. Cited 2023 Mar 11. 10.1186/1472-6963-10-225 PMC2924329 20682077

[52] Chou SY, Grossman M, Liu JT. The impact of National Health Insurance on birth outcomes: A natural experiment in Taiwan. J Dev Econ. 2014;111:75–91.

[53] Jeffrey AE, Newacheck PW. Role of Insurance for Children With Special Health Care Needs: A Synthesis of the Evidence. Pediatrics. 2006;118:e1027–38. Available from: https://www.pediatrics/article/118/4/e1027/69048/Role-of-Insurance-for-Children-With-Special-Health. Cited 2024 Jan 8. 10.1542/peds.2005-2527 16966391

[54] Miller NA, Kirk A, Kaiser MJ, Glos L. The relation between health insurance and health care disparities among adults with disabilities. Am J Public Health. 2014;104. Available from: https://pubmed.ncbi.nlm.nih.gov/24328621/. Cited 2023 Mar 9. 10.2105/AJPH.2013.301478 PMC3953783 24328621

[55] Dusing SC, Skinner AC, Mayer ML. Unmet need for therapy services, assistive devices, and related services: data from the national survey of children with special health care needs. Ambul Pediatr. 2004;4:448. 15369415 10.1367/A03-202R1.1

[56] Shin J, Moon S. Quality of Care and Role of Health Insurance Among Non-Elderly Women with Disabilities. Women’s Health Issues. 2008;18:238–48. 18590882 10.1016/j.whi.2008.02.005

[57] Szilagyi PG. Health Insurance and Children with Disabilities on JSTOR. JSTOR. 2012;22:123–48. Available from: https://www.jstor.org/stable/41475649. Cited 2024 Jan 23. 10.1353/foc.2012.0000 22550688

[58] Van Hees SGM, O’Fallon T, Hofker M, Dekker M, Polack S, Banks LM, et al. Leaving no one behind? Social inclusion of health insurance in low- and middle-income countries: a systematic review. Int J Equity Health. 2019;18:134 BioMed Central Ltd. 31462303 10.1186/s12939-019-1040-0 PMC6714392

[59] Banks LM, Mearkle R, Mactaggart I, Walsham M, Kuper H, Blanchet K. Disability and social protection programmes in low- and middle-income countries: a systematic review. Oxf Dev Stud. 2017;45:223–39.

[60] World Health Organization. Universal Health Coverage. Available from: https://www.who.int/health-topics/universal-health-coverage#tab=tab_1. Cited 2024 Oct 3.

[61] National Institute of Health. Study Quality Assessment Tools | NHLBI, NIH. July. 2021. Available from: https://www.nhlbi.nih.gov/health-topics/study-quality-assessment-tools. Cited 2024 Jan 5.

[62] Wiredu DNA, Peprah C, Agyemang-Duah W. Prevalence of health insurance enrolment and associated factors among persons with disabilities in Ghana. Cogent Med. 2021;8:117094.

[63] Huang J, Pan XL, Li A. Multi-level modelling of the factors that influence the participation of disabled rural individuals in social medical insurance in China. BMC Health Serv Res. 2013;13:58. 23402275 10.1186/1472-6963-13-58 PMC3598385

[64] Flores-Flores O, Bell R, Reynolds R, Bernabé-Ortiz A. Older adults with disability in extreme poverty in Peru: How is their access to health care? PLoS One. 2018;13:e0208441. 30586426 10.1371/journal.pone.0208441 PMC6306199

[65] Agbadi P, Okyere J, Lomotey A, Duah HO, Seidu AA, Ahinkorah BO. Socioeconomic and demographic correlates of nonenrolment onto the national health insurance scheme among children in Ghana: Insight from the 2017/18 multiple indicator cluster survey. Prev Med Rep. 2021;22:101385. 33996397 10.1016/j.pmedr.2021.101385 PMC8102991

[66] Atchessi N, Ridde V, Zunzunégui M-V. Is the process for selecting indigents to receive free care in Burkina Faso equitable?. 2014. Available from: http://www.biomedcentral.com/1471-2458/14/1158. 10.1186/1471-2458-14-1158 PMC4242543 25377858

[67] Barreto MCA, Araújo LF, de Castro SS. Relationship between personal and environmental factors and prevalence of acquired physical impairment in Brazil-a population-based study. Ciencia e Saude Coletiva. 2022;27:1435–42. 35475824 10.1590/1413-81232022274.06472021

[68] Bernabe-Ortiz A, Diez-Canseco F, Vasquez A, Kuper H, Walsham M, Blanchet K. Inclusion of persons with disabilities in systems of social protection: a population-based survey and case-control study in Peru. BMJ Open. 2016;6:e011300. 27566630 10.1136/bmjopen-2016-011300 PMC5013477

[69] Doubova S V, Pérez-Cuevas R, Canning D, Reich MR. Access to healthcare and financial risk protection for older adults in Mexico: secondary data analysis of a national survey. 2015;5:7877. Available from: http://bmjopen.bmj.com/. 10.1136/bmjopen-2015-007877 PMC4513520 26198427

[70] Gómez F, Osorio-García D, Panesso L, Curcio CL. Healthy aging determinants and disability among older adults: SABE Colombia. Revista Panamericana de Salud Publica/Pan Am J Public Health. 2021;45:e98. 10.26633/RPSP.2021.98 PMC8369129 34475887

[71] Hao F, Wang B, Tan W, Husain SF, McIntyre RS, Tang X, et al. Attitudes toward COVID-19 vaccination and willingness to pay: comparison of people with and without mental disorders in China. BJPsych Open. 2021;7:e146. 34422295 10.1192/bjo.2021.979 PMC8365102

[72] Lund C, Docrat S, Abdulmalik J, Alem A, Fekadu A, Gureje O, et al. Household economic costs associated with mental, neurological and substance use disorders: a cross-sectional survey in six low- and middle-income countries. BJPsych Open. 2019;5:e34. 31530317 10.1192/bjo.2019.20 PMC6469228

[73] Palmer M, Nguyen T, Neeman T, Berry H, Hull T, Harley D. Health care utilization, cost burden and coping strategies by disability status: an analysis of the Viet Nam National Health Survey. Int J Health Plan Manage. 2011;26:e151–68. 10.1002/hpm.1052 20583316

[74] Palmer MG, Nguyen TMT. Mainstreaming health insurance for people with disabilities. J Asian Econ. 2012;23:600–13.

[75] Pengpid S, Peltzer K. Prevalence and correlates of functional disability among community-dwelling older adults in India: results of a national survey in 2017–2019. Elderly Health J. 2021;7:18.

[76] Van Der Wielen N, Falkingham J, Channon AA. Determinants of National Health Insurance enrolment in Ghana across the life course: are the results consistent between surveys? Int J Equity Health. 2018;17:49. 29685137 10.1186/s12939-018-0760-x PMC5913914

[77] Yu W, Singh SS, Calhoun S, Zhang H, Zhao X, Yang F. Generalized anxiety disorder in urban China: prevalence, awareness, and disease burden. J Affect Disord. 2018;234:89–96. 29524751 10.1016/j.jad.2018.02.012

[78] Finnoff K. Gender disparity in access to the Rwandan mutual health insurance scheme. Fem Econ. 2016;22:26–50.

[79] Carnero Contentti E, Giachello S, Correale J. Barriers to access and utilization of multiple sclerosis care services in a large cohort of Latin American patients. Mult Scler J. 2021;27:117–29. 10.1177/1352458519898590 31961260

[80] Guo C, Du W, Hu C, Zheng X. Prevalence and factors associated with healthcare service use among Chinese elderly with disabilities. J Public Health (United Kingdom). 2015;38:e345–51. 10.1093/pubmed/fdv120 26408823

[81] Guo C, Li N, Chen G, Zheng X. Mental health service utilization and its associated social factors among elderly people with a mental disability in China: a national population-based survey. Scand J Public Health. 2017;47:215–20. 28784028 10.1177/1403494817722705

[82] Mai S, Cai J, Li L. Factors associated with access to healthcare services for older adults with limited activities of daily living. 2022. Available from: http://opendata.pku.edu.cn/dataverse/CHADS. 10.3389/fpubh.2022.921980 PMC9583939 36276353

[83] de Medeiros AA, Galvão MHR, Barbosa IR, da Oliveira AGRC. Use of rehabilitation services by persons with disabilities in Brazil: a multivariate analysis from Andersen’s behavioral model. PLoS One. 2021;16:e0250615. 33914791 10.1371/journal.pone.0250615 PMC8084141

[84] Moradi G, Bolbanabad AM, Abdullah FZ, Safari H, Rezaei S, Aghaei A, et al. Catastrophic health expenditures for children with disabilities in Iran: a national survey. Int J Health Plan Manage. 2021;36:1861–73. 10.1002/hpm.3273 34185916

[85] Nartey AK, Badu E, Agyei-Baffour P, Gyamfi N, Opoku MP, O’Brien AP, et al. The predictors of treatment pathways to mental health services among consumers in Ghana. Perspect Psychiatr Care. 2019;55:300–10. 30648278 10.1111/ppc.12350

[86] Shi J, Tang L, Jing L, Geng J, Liu R, Luo L, et al. Disparities in mental health care utilization among inpatients in various types of health institutions: A cross-sectional study based on EHR data in Shanghai China. BMC Public Health. 2019;19:1023. 31366334 10.1186/s12889-019-7343-7 PMC6668074

[87] Chen H, Ning J. Public long-term care insurance scheme and informal care use among community-dwelling older adults in China. Health Soc Care Community. 2022;30:e6386–95. 36254815 10.1111/hsc.14080

[88] Palmer MG. Inequalities in universal health coverage: evidence from Vietnam. World Dev. 2014;64:384–94.

[89] Shiwakoti R, Gurung YB, Poudel RC, Neupane S, Thapa RK, Deuja S, et al. Factors affecting utilization of sexual and reproductive health services among women with disabilities- a mixed-method cross-sectional study from Ilam district Nepal. BMC Health Serv Res. 2021;21:1361. 34949185 10.1186/s12913-021-07382-4 PMC8705122

[90] El-Sayed AM, Palma A, Freedman LP, Kruk ME. Does health insurance mitigate inequities in non-communicable disease treatment? Evidence from 48 low- and middle-income countries. Health Policy (New York). 2015;119:1164–75. 10.1016/j.healthpol.2015.07.006 PMC5927367 26271138

[91] Fan H, Wang Y, Gao J, Peng Z, Coyte PC. The effect of a long-term care insurance program on subjective well-being of older adults with a disability: quasi-experimental evidence from China. J Appl Gerontol. 2023;42:438–46. 36366866 10.1177/07334648221138282

[92] Li N, Du W, Chen G, Song X, Zheng X. Mental health service use among chinese adults with mental disabilities: a national survey. Psychiatr Serv. 2013;64:638–44. 23411995 10.1176/appi.ps.001232012

[93] MacHnicki G, Dillon C, Allegri RF. Insurance status and demographic and clinical factors associated with pharmacologic treatment of depression: Associations in a cohort in Buenos Aires. Value in Health. 2011;14:S13. 21839885 10.1016/j.jval.2011.05.014

[94] Nattaj AH, Zarghami M, Yazdani-Charati J, Vahedi M, Sheikholeslami A, Faghani Z. Factors associated with the length of first time hospitalization in a referral psychiatric Hospital in north of Iran. Iran J Psychiatry Behav Sci. 2017;11..

[95] Zhang H, Sun Y, Zhang D, Zhang C, Chen G. Direct medical costs for patients with schizophrenia: A 4-year cohort study from health insurance claims data in Guangzhou city, Southern China. Int J Ment Health Syst. 2018;12:1–14. 10.1186/s13033-018-0251-x PMC6251138 30479658

[96] Chen H, Ning J. The impacts of long-term care insurance on health care utilization and expenditure: evidence from China. Health Pol Plan. 2022;37:717–27. 10.1093/heapol/czac003 35032390

[97] Guan X, Fu M, Lin F, Zhu D, Vuillermin D, Shi L. Burden of visual impairment associated with eye diseases: exploratory survey of 298 Chinese patients. BMJ Open. 2019;9:e030561. 31515429 10.1136/bmjopen-2019-030561 PMC6747637

[98] Mohammed FMS, Ali SM, Mustafa MAA. Quality of life of cerebral palsy patients and their caregivers: a cross sectional study in a rehabilitation center Khartoum-Sudan (2014–2015). J Neurosci Rural Pract. 2019;7:355–61. 10.4103/0976-3147.182778 PMC4898102 27365951

[99] Tien NLB, Van Thanh V, Hanh KTH, Anh PG, Huyen LTM, Tu NT, et al. Quality of life and activities of daily living among patients with complete cervical spinal cord injury and surgical treatment in Vietnam. Int J Environ Res Public Health. 2021;18:9703. 34574629 10.3390/ijerph18189703 PMC8465366

[100] Odipo E, Jarhyan P, Nzinga J, Prabhakaran D, Aryal A, Clarke-Deelder E, et al. The People’s Voice Survey on Health System Performance 3 The path to universal health coverage in five African and Asian countries: examining the association between insurance status and health-care use. www.thelancet.com/lancetgh. 2024;12:123. Available from: www.thelancet.com/lancetgh. Cited 2024 Jan 8. 10.1016/S2214-109X(23)00510-7 PMC10716621 38096884

[101] Erlangga D, Suhrcke M, Ali S, Bloor K. The impact of public health insurance on health care utilisation, financial protection and health status in low- And middle-income countries: a systematic review. PLoS One. 2019;14:e0225237. 31461458 10.1371/journal.pone.0219731 PMC6713352

[102] Skinner AC, Mayer ML. Effects of insurance status on children’s access to specialty care: a systematic review of the literature. BMC Health Serv Res. 2007;7. Available from: https://pubmed.ncbi.nlm.nih.gov/18045482/. Cited 2024 Jan 8. 10.1186/1472-6963-7-194 PMC2222624 18045482

[103] Prinja S, Chauhan AS, Karan A, Kaur G, Kumar R. Impact of Publicly Financed Health Insurance Schemes on Healthcare Utilization and Financial Risk Protection in India: A Systematic Review. PLoS One. 2017;12. Available from: https://pubmed.ncbi.nlm.nih.gov/28151946/. Cited 2024 Jan 8. 10.1371/journal.pone.0170996 PMC5289511 28151946

[104] Eze P, Ilechukwu S, Lawani LO. Impact of community-based health insurance in low- and middle-income countries: A systematic review and meta-analysis. PLoS One. 2023;18. Available from: https://pubmed.ncbi.nlm.nih.gov/37368882/. Cited 2024 Jan 8. 10.1371/journal.pone.0287600 PMC10298805 37368882

[105] Spaan E, Mathijssen J, Tromp N, McBain F, ten Have A, Baltussen R. The impact of health insurance in Africa and Asia: a systematic review. Bull World Health Organ. 2012;90:685–92. Available from: https://pubmed.ncbi.nlm.nih.gov/22984313/. Cited 2024 Jan 8. 10.2471/BLT.12.102301 PMC3442382 22984313

[106] Docrat S, Besada D, Cleary S, Lund C. The impact of social, national and community-based health insurance on health care utilization for mental, neurological and substance-use disorders in low-and middle-income countries: a systematic review. Health Econ Rev. 2020;10. Available from: 10.1186/s13561-020-00268-x. Cited 2024 Jan 8. 10.1186/s13561-020-00268-x PMC7181535 32333114

[107] Minden SL, Hoaglin DC, Hadden L, Frankel D, Robbins T, Perloff J. Access to and utilization of neurologists by people with multiple sclerosis. Neurology. 2008;70:1141–9. Available from: https://pubmed.ncbi.nlm.nih.gov/18362274/. Cited 2024 Jan 9. 10.1212/01.wnl.0000306411.46934.ef 18362274

[108] Chen S, Li L, Yang J, Jiao L, Golden T, Wang Z, et al. The impact of long-term care insurance in China on beneficiaries and caregivers: A systematic review. Journal of global health economics and policy. 2021;1. Available from: https://pubmed.ncbi.nlm.nih.gov/35083471/. Cited 2024 Jan 8. 10.52872/001c.29559 PMC8788994 35083471

[109] Okoroh J, Essoun S, Seddoh A, Harris H, Weissman JS, Dsane-Selby L, et al. Evaluating the impact of the national health insurance scheme of Ghana on out of pocket expenditures: a systematic review. BMC Health Serv Res. 2018;18. Available from: https://pubmed.ncbi.nlm.nih.gov/29879978/. Cited 2024 Jan 8. 10.1186/s12913-018-3249-9 PMC5992790 29879978

[110] Habib SS, Perveen S, Khuwaja HMA. The role of micro health insurance in providing financial risk protection in developing countries--a systematic review. BMC Public Health. 2016;16. Available from: https://pubmed.ncbi.nlm.nih.gov/27004824/. Cited 2024 Jan 8. 10.1186/s12889-016-2937-9 PMC4802630 27004824

[111] Li F, Wu Y, Yuan Q, Zou K, Yang M, Chen D. Do health insurances reduce catastrophic health expenditure in China? A systematic evidence synthesis. PLoS One. 2020;15. Available from: https://pubmed.ncbi.nlm.nih.gov/32970740/. Cited 2024 Jan 8. 10.1371/journal.pone.0239461 PMC7514005 32970740

[112] Odonkor SNNT, Koranteng F, Appiah-Danquah M, Dini L. Do national health insurance schemes guarantee financial risk protection in the drive towards Universal Health Coverage in West Africa? A systematic review of observational studies. PLOS Global Public Health. 2023;3:e0001286. 37556426 10.1371/journal.pgph.0001286 PMC10411819

[113] Bayked EM, Toleha HN, Kebede SZ, Workneh BD, Kahissay MH. The impact of community-based health insurance on universal health coverage in Ethiopia: a systematic review and meta-analysis. Glob Health Action. Taylor and Francis Ltd.; 2023. 10.1080/16549716.2023.2189764 PMC10035959 36947450

[114] Choi JW, Shin JY, Cho KH, Nam JY, Kim JY, Lee SG. Medical security and catastrophic health expenditures among households containing persons with disabilities in Korea: A longitudinal population-based study. Int J Equity Health. 2016;15:1–8. 10.1186/s12939-016-0406-9. 10.1186/s12939-016-0406-9 PMC4962408 27459992

[115] Abodey E, Vanderpuye I, Mensah I, Badu E. In search of universal health coverage - highlighting the accessibility of health care to students with disabilities in Ghana: a qualitative study. BMC Health Serv Res. 2020;20. Available from: https://pubmed.ncbi.nlm.nih.gov/32234049/. Cited 2023 Mar 8. 10.1186/s12913-020-05138-0 PMC7106671 32234049

[116] Dassah E, Aldersey H, McColl MA, Davison C. Factors affecting access to primary health care services for persons with disabilities in rural areas: a “best-fit” framework synthesis. Glob Health Res Policy. 2018;3:1–13. Available from: https://ghrp.biomedcentral.com/articles/10.1186/s41256-018-0091-x. Cited 2024 Mar 4. 10.1186/s41256-018-0091-x PMC6305566 30603678

[117] Wang JT, Xu G, Ren RJ, Wang Y, Tang R, Huang Q, et al. The impacts of health insurance and resource on the burden of Alzheimer’s disease and related dementias in the world population. Alzheimer’s & Dementia. 2023;19:967–79. Available from: https://onlinelibrary.wiley.com/doi/full/10.1002/alz.12730. Cited 2024 Jan 29. 10.1002/alz.12730 35820032

