# Supplementary material for: Access to health insurance amongst people with disabilities and its association with healthcare use, health status and financial protection in low- and middle-income countries: a systematic review
Source: Int J Equity Health. 2024 Dec 18;23:264. doi: 10.1186/s12939-024-02339-5 (PMC11658242; doi:10.1186/s12939-024-02339-5)
Supplement: Supplementary file 1 — Additional file 1. Search strategies. [file 12939_2024_2339_MOESM1_ESM.zip › Additional file 1. Search Strategies 9 databases V7.docx]

Additional file 1. Search Strategies

1. Medline Ovid

Search date: January 24, 2023

| **No** | **Search Terms** | **Result** |
| --- | --- | --- |
| 1 | exp Insurance, health/ or exp Insurance, disability/ | 166596 |
| 2 | (Health insurance* or medical aid or medical insurance* or universal health coverage or health fund* or health financ* or social health protection* or social health insurance* or community health fund* or community-based insurance* or health financing or (health adj3 insuranc*) or disability insuranc*).ti,ab. | 64168 |
| 3 | 1 or 2 | 212720 |
| 4 | exp disabled persons/ or exp disabled children/ | 72575 |
| 5 | ((disabilit* or disable* or handicap* or function* limitation* or function* diversit* or dependen* or special need* or rare disease* or incapacity* or impairment*) adj5 (person* or people or individ* or patient* or subject* or adult* or elderly or child* or boy* or girl* or kid* or m?n or wom?n or teenager* or juvenile or adolescent*)).ti,ab. | 263467 |
| 6 | exp cerebral palsy/ or exp spinal dysraphism/ or exp osteogenesis imperfecta/ or exp limb deformities, congenital/ or exp amputation/ or exp amputation, traumatic/ or exp arthrogryposis/ or exp clubfoot/ or exp poliomyelitis/ or exp paralysis/ or exp spastic paraplegia, hereditary/ or exp paraplegia/ or exp quadriplegia/ or exp spinal cord injuries/ | 245515 |
| 7 | (Physical* adj3 (impair* or deficienc* or disable* or disabilit* or handicap* or incapacity*)).ti,ab. | 20922 |
| 8 | (Cerebral pals* or spina bifida or muscular distroph* or osteogenesis imperfecta or polio or poliomyelitis or paralyz* or paralys* or tetraplegi* or quadriplegi* or paraplegi* or hemiplegi* or wheelchair user* or wheel chair user* or amput*).ti,ab. | 201505 |
| 9 | exp hearing loss/ or exp persons with hearing impairments/ | 78014 |
| 10 | ((deaf* or deaf-blind disorder* or ((hearing or acoustic) adj3 (loss* or impair* or deficienc* or disable* or disabilit* or handicap*))) not (blinding or double blind or triple blind)).ti,ab. | 96589 |
| 11 | exp vision disorders/ | 77959 |
| 12 | ((Blind* or ((visual* or vision) adj3 (loss* disabilit* or disorder* or impairment* or disable* or deficienc* or handicap*))) not (blinding or double blind or triple blind)).ti,ab. | 195297 |
| 13 | exp speech disorders/ or exp communication disorders/ | 68334 |
| 14 | ((speech* or communication*) adj3 (disabilit* or disorder* or impair* or deficienc* or disable* or handicap*)).ti,ab. | 13370 |
| 15 | exp intellectual disability/ or exp cognition disorders/ or exp developmental disabilities/ or exp learning disabilities/ | 253718 |
| 16 | (((intellectual or cognition or cognitive or learning or developmental) adj3 (disabilit* or disable* or impair* or disorder* or incapacit* or handicap*)) or autism* or ADHD or attention deficit hyperactivity disorder* or attention-deficit hyperactivity disorder* or down syndrome or dementia or Alzheimer).ti,ab. | 384694 |
| 17 | exp mental disorders/ or exp mentally ill persons/ | 1412269 |
| 18 | (((Mental* or psychological* or behavior*) adj3 (disabilit* or disable* or impair* or handicap* or incapacit* or disorder* or illness* or ill* or dysfunction* or retard* or deficienc* or disease* or diagnos*)) or psychosis or psychoses or schizoaffective or schizophreniform or schizophrenia or bipolar or mental health condition*).ti,ab. | 401242 |
| 19 | exp self-help devices/ or exp sensory aids/ or exp orthopedic equipment/ | 147099 |
| 20 | (Magnifier* or medical device* or ((assistive or mobilit*) adj3 (device* or technolog* or product* or equipment* or tool*)) or hearing aid* or wheelchair* or wheel chair* or orthotic* or prosthetic*).ti,ab. | 109707 |
| 21 | exp rehabilitation/ | 347475 |
| 22 | ((physical* or mental* or cognitive* or occupation* or speech* or voice* or vocation*) adj3 (rehabilitat* or therap*)).ti,ab. | 95119 |
| 23 | 4 or 5 or 6 or 7 or 8 or 9 or 10 or 11 or 12 or 13 or 14 or 15 or 16 or 17 or 18 or 19 or 20 or 21 or 22 | 3031919 |
| 24 | developing country/ or low income country/ or middle income country/ | 80307 |
| 25 | ((developing or less* developed or under developed or underdeveloped or middle income or low* income) adj (economy or economies)).ti,ab. | 906 |
| 26 | ((developing or less* developed or under developed or underdeveloped or middle income or low* income or underserved or under served or deprived or poor*) adj (countr* or nation? or population? or world)).ti,ab. | 129229 |
| 27 | (low* adj (gdp or gnp or gross domestic or gross national)).ti,ab. | 338 |
| 28 | (low adj3 middle adj3 countr*).ti,ab. | 28037 |
| 29 | (lmic or lmics or third world or lami countr*).ti,ab. | 12006 |
| 30 | transitional countr*.ti,ab. | 176 |
| 31 | global south.ti,ab. | 920 |
| 32 | "Africa south of the Sahara"/ | 13050 |
| 33 | ("Africa South of the Sahara" or sub-Saharan Africa or subSaharan Africa).ti,ab. | 27675 |
| 34 | Central Africa.ti,ab. | 3672 |
| 35 | Eastern Africa.ti,ab. | 1229 |
| 36 | Southern Africa.ti,ab. | 5061 |
| 37 | Western Africa.ti,ab. | 1014 |
| 38 | North Korea/ | 289 |
| 39 | (North Korea or (Democratic People* Republic adj2 Korea)).ti,ab. | 507 |
| 40 | Haiti/ | 3544 |
| 41 | (Haiti or Hayti).ti,ab. | 3441 |
| 42 | Afghanistan/ | 3733 |
| 43 | Afghanistan.ti,ab. | 6876 |
| 44 | Nepal/ | 10445 |
| 45 | Nepal.ti,ab. | 12744 |
| 46 | Syrian Arab Republic/ | 0 |
| 47 | (Syria or Syrian Arab Republic).ti,ab. | 2618 |
| 48 | Yemen/ | 1555 |
| 49 | Yemen.ti,ab. | 2168 |
| 50 | Tajikistan/ | 802 |
| 51 | Tajikistan.ti,ab. | 738 |
| 52 | Benin/ | 1881 |
| 53 | (Benin or Dahomey).ti,ab. | 4045 |
| 54 | Burkina Faso/ | 3872 |
| 55 | (Burkina Faso or Burkina Fasso or Upper Volta).ti,ab. | 5105 |
| 56 | Burundi/ | 722 |
| 57 | (Burundi or Ruanda-Urundi).ti,ab. | 1092 |
| 58 | Central African Republic/ | 836 |
| 59 | (Central African Republic or Ubangi-Shari).ti,ab. | 1136 |
| 60 | Chad/ | 812 |
| 61 | Chad.ti,ab. | 1445 |
| 62 | Democratic Republic Congo/ | 0 |
| 63 | (((Democratic Republic or DR) adj2 Congo) or Congo-Kinshasa or Belgian Congo or Zaire or Congo Free State).ti,ab. | 5291 |
| 64 | Eritrea/ | 408 |
| 65 | Eritrea.ti,ab. | 667 |
| 66 | Ethiopia/ | 18406 |
| 67 | (Ethiopia or Abyssinia).ti,ab. | 25168 |
| 68 | Gambia/ | 2650 |
| 69 | Gambia.ti,ab. | 2628 |
| 70 | Guinea/ | 1281 |
| 71 | (Guinea not (New Guinea or Guinea Pig* or Guinea Fowl or Guinea-Bissau or Portuguese Guinea or Equatorial Guinea)).ti,ab. | 3170 |
| 72 | Guinea-Bissau/ | 1020 |
| 73 | (Guinea-Bissau or Portuguese Guinea).ti,ab. | 1154 |
| 74 | Liberia/ | 1365 |
| 75 | Liberia.ti,ab. | 1838 |
| 76 | Madagascar/ | 3872 |
| 77 | (Madagascar or Malagasy Republic).ti,ab. | 5524 |
| 78 | Malawi/ | 6571 |
| 79 | (Malawi or Nyasaland).ti,ab. | 8581 |
| 80 | Mali/ | 2658 |
| 81 | Mali.ti,ab. | 4155 |
| 82 | Mozambique/ | 2965 |
| 83 | (Mozambique or Mocambique or Portuguese East Africa).ti,ab. | 4432 |
| 84 | Niger/ | 1384 |
| 85 | (Niger not (Aspergillus or Peptococcus or Schizothorax or Cruciferae or Gobius or Lasius or Agelastes or Melanosuchus or radish or Parastromateus or Orius or Apergillus or Parastromateus or Stomoxys)).ti,ab. | 3991 |
| 86 | Rwanda/ | 3066 |
| 87 | (Rwanda or Ruanda).ti,ab. | 3943 |
| 88 | Sierra Leone/ | 1890 |
| 89 | (Sierra Leone or Salone).ti,ab. | 2765 |
| 90 | Somalia/ | 1842 |
| 91 | (Somalia or Somaliland).ti,ab. | 1879 |
| 92 | south sudan/ | 264 |
| 93 | South Sudan.ti,ab. | 746 |
| 94 | Tanzania/ | 13526 |
| 95 | (Tanzania or Tanganyika or Zanzibar).ti,ab. | 16462 |
| 96 | Togo/ | 1267 |
| 97 | (Togo or Togolese Republic or Togoland).ti,ab. | 1751 |
| 98 | Uganda/ | 14896 |
| 99 | Uganda.ti,ab. | 17908 |
| 100 | Cambodia/ | 3796 |
| 101 | Cambodia.ti,ab. | 4650 |
| 102 | exp Indonesia/ | 13237 |
| 103 | (Indonesia or Dutch East Indies).ti,ab. | 17222 |
| 104 | kiribati/ | 1263 |
| 105 | (Kiribati or Gilbert Islands or Phoenix Islands or Line Islands).ti,ab. | 289 |
| 106 | Laos/ | 2229 |
| 107 | (Laos or (Lao adj1 Democratic Republic)).ti,ab. | 2432 |
| 108 | exp "Federated States of Micronesia"/ | 0 |
| 109 | Micronesia.ti,ab. | 736 |
| 110 | Mongolia/ | 2068 |
| 111 | Mongolia.ti,ab. | 5343 |
| 112 | Myanmar/ | 3129 |
| 113 | (Myanmar or Burma).ti,ab. | 5349 |
| 114 | Papua New Guinea/ | 3714 |
| 115 | (Papua New Guinea or German New Guinea or British New Guinea or Territory of Papua).ti,ab. | 4906 |
| 116 | Philippines/ | 9446 |
| 117 | (Philippines or Philippine Islands).ti,ab. | 10300 |
| 118 | solomon islands/ | 1147 |
| 119 | Solomon Islands.ti,ab. | 931 |
| 120 | Timor-Leste/ | 263 |
| 121 | (Timor-Leste or East Timor or Portuguese Timor).ti,ab. | 632 |
| 122 | Vanuatu/ | 418 |
| 123 | (Vanuatu or New Hebrides).ti,ab. | 812 |
| 124 | Viet Nam/ | 14474 |
| 125 | (Viet Nam or Vietnam or French Indochina).ti,ab. | 19017 |
| 126 | Kyrgyzstan/ | 1388 |
| 127 | (Kyrgyzstan or Kyrgyz Republic or Kirghizia or Kirghiz).ti,ab. | 1197 |
| 128 | Moldova/ | 743 |
| 129 | Moldova.ti,ab. | 653 |
| 130 | exp Ukraine/ | 16966 |
| 131 | Ukraine.ti,ab. | 6274 |
| 132 | exp Uzbekistan/ | 1987 |
| 133 | Uzbekistan.ti,ab. | 1320 |
| 134 | Bolivia/ | 2821 |
| 135 | Bolivia.ti,ab. | 3723 |
| 136 | El Salvador/ | 947 |
| 137 | El Salvador.ti,ab. | 1462 |
| 138 | Honduras/ | 1244 |
| 139 | Honduras.ti,ab. | 2036 |
| 140 | Nicaragua/ | 1611 |
| 141 | Nicaragua.ti,ab. | 2109 |
| 142 | Djibouti/ | 250 |
| 143 | (Djibouti or French Somaliland).ti,ab. | 437 |
| 144 | Egypt/ | 17218 |
| 145 | Egypt.ti,ab. | 17278 |
| 146 | Morocco/ | 6471 |
| 147 | Morocco.ti,ab. | 6923 |
| 148 | Tunisia/ | 9181 |
| 149 | Tunisia.mp. | 11848 |
| 150 | palestine/ | 0 |
| 151 | (Gaza or West Bank or Palestine).ti,ab. | 3133 |
| 152 | Bangladesh/ | 14023 |
| 153 | Bangladesh.ti,ab. | 18207 |
| 154 | Bhutan/ | 663 |
| 155 | Bhutan.ti,ab. | 1047 |
| 156 | exp India/ | 117327 |
| 157 | India.ti,ab. | 124618 |
| 158 | exp Pakistan/ | 22042 |
| 159 | Pakistan.ti,ab. | 25331 |
| 160 | Angola/ | 1126 |
| 161 | Angola.ti,ab. | 1672 |
| 162 | Cameroon/ | 6412 |
| 163 | (Cameroon or Kamerun or Cameroun).ti,ab. | 8310 |
| 164 | Cape Verde/ | 248 |
| 165 | (Cape Verde or Cabo Verde).ti,ab. | 741 |
| 166 | Comoros/ | 373 |
| 167 | (Comoros or Glorioso Islands or Mayotte).ti,ab. | 703 |
| 168 | Congo/ | 2001 |
| 169 | (Congo not ((Democratic Republic adj3 Congo) or congo red or crimean-congo)).ti,ab. | 2918 |
| 170 | Cote d'Ivoire/ | 3505 |
| 171 | (Cote d'Ivoire or Cote dIvoire or Ivory Coast).ti,ab. | 4372 |
| 172 | eswatini/ | 745 |
| 173 | (eSwatini or Swaziland).ti,ab. | 1137 |
| 174 | Ghana/ | 10818 |
| 175 | (Ghana or Gold Coast).ti,ab. | 14309 |
| 176 | Kenya/ | 19049 |
| 177 | (Kenya or East Africa Protectorate).ti,ab. | 21771 |
| 178 | Lesotho/ | 509 |
| 179 | (Lesotho or Basutoland).ti,ab. | 890 |
| 180 | Mauritania/ | 495 |
| 181 | Mauritania.ti,ab. | 726 |
| 182 | Nigeria/ | 33358 |
| 183 | Nigeria.ti,ab. | 34789 |
| 184 | "sao tome and principe"/ | 31 |
| 185 | (Sao Tome adj2 Principe).ti,ab. | 182 |
| 186 | Senegal/ | 6176 |
| 187 | Senegal.ti,ab. | 6413 |
| 188 | Sudan/ | 5112 |
| 189 | (Sudan not South Sudan).ti,ab. | 8433 |
| 190 | Zambia/ | 5385 |
| 191 | (Zambia or Northern Rhodesia).ti,ab. | 6433 |
| 192 | Zimbabwe/ | 6496 |
| 193 | (Zimbabwe or Southern Rhodesia).ti,ab. | 6683 |
| 194 | American Samoa/ | 208 |
| 195 | American Samoa.ti,ab. | 405 |
| 196 | china/ or guangxi/ or inner mongolia/ or macao/ or ningxia/ or tibet/ or xinjiang/ | 245400 |
| 197 | China.ti,ab. | 272247 |
| 198 | Fiji/ | 1114 |
| 199 | Fiji.ti,ab. | 2219 |
| 200 | exp Malaysia/ | 17790 |
| 201 | (Malaysia or Malayan Union or Malaya).ti,ab. | 20933 |
| 202 | marshall islands/ | 1263 |
| 203 | Marshall Islands.ti,ab. | 341 |
| 204 | nauru/ | 1263 |
| 205 | Nauru.ti,ab. | 169 |
| 206 | Samoa/ | 392 |
| 207 | ((Samoa not American Samoa) or Western Samoa or Navigator Islands or Samoan Islands).ti,ab. | 667 |
| 208 | Thailand/ | 30308 |
| 209 | (Thailand or Siam).ti,ab. | 32314 |
| 210 | Tonga/ | 292 |
| 211 | Tonga.ti,ab. | 527 |
| 212 | tuvalu/ | 1263 |
| 213 | (Tuvalu or Ellice Islands).ti,ab. | 86 |
| 214 | Albania/ | 955 |
| 215 | Albania.ti,ab. | 1258 |
| 216 | Armenia/ | 1557 |
| 217 | Armenia.ti,ab. | 1339 |
| 218 | exp Azerbaijan/ | 1288 |
| 219 | Azerbaijan.ti,ab. | 1643 |
| 220 | Belarus/ | 2151 |
| 221 | (Belarus or Byelarus or Byelorussia or Belorussia).ti,ab. | 1756 |
| 222 | exp "Bosnia and Herzegovina"/ | 2336 |
| 223 | (Bosnia or Herzegovina).ti,ab. | 2710 |
| 224 | Bulgaria/ | 6638 |
| 225 | Bulgaria.ti,ab. | 4775 |
| 226 | exp "Georgia (republic)"/ | 1968 |
| 227 | Georgia.ti,ab. not "georgia (u.s.)"/ | 11659 |
| 228 | Kazakhstan/ | 3030 |
| 229 | (Kazakhstan or Kazakh).ti,ab. | 3615 |
| 230 | Kosovo/ | 298 |
| 231 | Kosovo.ti,ab. | 1091 |
| 232 | "Montenegro (republic)"/ | 0 |
| 233 | Montenegro.ti,ab. | 1007 |
| 234 | "republic of north macedonia"/ | 630 |
| 235 | North Macedonia.ti,ab. | 199 |
| 236 | Romania/ | 10885 |
| 237 | Romania.ti,ab. | 7307 |
| 238 | exp Russian Federation/ | 0 |
| 239 | ussr/ | 42848 |
| 240 | (Russia or Russian Federation or USSR or Union of Soviet Socialist Republics or Soviet Union).ti,ab. | 32524 |
| 241 | exp Serbia/ | 3694 |
| 242 | Serbia.ti,ab. | 5381 |
| 243 | "Turkey (republic)"/ | 0 |
| 244 | (Turkey.ti,ab. not "Turkey (bird)"/) or (Anatolia or Asia Minor).ti,ab. | 43319 |
| 245 | Turkmenistan/ | 590 |
| 246 | Turkmenistan.ti,ab. | 414 |
| 247 | Argentina/ | 17714 |
| 248 | (Argentina or Argentine Republic).ti,ab. | 19763 |
| 249 | Belize/ | 630 |
| 250 | (Belize or British Honduras).ti,ab. | 972 |
| 251 | exp Brazil/ | 114015 |
| 252 | Brazil.ti,ab. | 102294 |
| 253 | Colombia/ | 12873 |
| 254 | Colombia.ti,ab. | 15734 |
| 255 | Costa Rica/ | 3987 |
| 256 | Costa Rica.ti,ab. | 5566 |
| 257 | Cuba/ | 5342 |
| 258 | Cuba.ti,ab. | 5006 |
| 259 | Dominica/ | 114 |
| 260 | Dominica.ti,ab. | 556 |
| 261 | Dominican Republic/ | 1737 |
| 262 | Dominican Republic.ti,ab. | 2243 |
| 263 | Ecuador/ | 4550 |
| 264 | Ecuador.ti,ab. | 5837 |
| 265 | Grenada/ | 166 |
| 266 | Grenada.ti,ab. | 363 |
| 267 | Guatemala/ | 3300 |
| 268 | Guatemala.ti,ab. | 4091 |
| 269 | Guyana/ | 745 |
| 270 | (Guyana or British Guiana).ti,ab. | 1221 |
| 271 | Jamaica/ | 3592 |
| 272 | Jamaica.ti,ab. | 3490 |
| 273 | exp Mexico/ | 43481 |
| 274 | (Mexico or United Mexican States).ti,ab. | 49768 |
| 275 | Paraguay/ | 901 |
| 276 | Paraguay.mp. | 2048 |
| 277 | Peru/ | 10564 |
| 278 | Peru.ti,ab. | 12963 |
| 279 | Saint Lucia/ | 75 |
| 280 | (St Lucia or Saint Lucia or Iyonala or Hewanorra).ti,ab. | 372 |
| 281 | "Saint Vincent and the Grenadines"/ | 58 |
| 282 | (Saint Vincent or St Vincent or Grenadines).ti,ab. | 657 |
| 283 | Suriname/ | 1003 |
| 284 | (Suriname or Dutch Guiana).ti,ab. | 705 |
| 285 | Venezuela/ | 5122 |
| 286 | Venezuela.ti,ab. | 5735 |
| 287 | Algeria/ | 3506 |
| 288 | Algeria.ti,ab. | 3995 |
| 289 | Iran/ | 36100 |
| 290 | (Iran or Persia).ti,ab. | 49396 |
| 291 | exp Iraq/ | 5368 |
| 292 | (Iraq or Mesopotamia).ti,ab. | 8418 |
| 293 | Jordan/ | 5263 |
| 294 | Jordan.ti,ab. | 8058 |
| 295 | Lebanon/ | 5131 |
| 296 | (Lebanon or Lebanese Republic).ti,ab. | 5791 |
| 297 | Libyan Arab Jamahiriya/ | 0 |
| 298 | libya.ti,ab. | 1462 |
| 299 | maldives/ | 839 |
| 300 | Maldives.ti,ab. | 462 |
| 301 | Sri Lanka/ | 6986 |
| 302 | (Sri Lanka or Ceylon).ti,ab. | 8413 |
| 303 | Botswana/ | 2151 |
| 304 | (Botswana or Bechuanaland or Kalahari).ti,ab. | 3106 |
| 305 | Equatorial Guinea/ | 309 |
| 306 | (Equatorial Guinea or Spanish Guinea).ti,ab. | 485 |
| 307 | Gabon/ | 1618 |
| 308 | (Gabon or Gabonese Republic).ti,ab. | 1981 |
| 309 | Mauritius/ | 621 |
| 310 | (Mauritius or Agalega Islands).ti,ab. | 1148 |
| 311 | Namibia/ | 1288 |
| 312 | (Namibia or German South West Africa).ti,ab. | 1932 |
| 313 | South Africa/ | 48581 |
| 314 | (South Africa or Cape Colony or British Bechuanaland or Boer Republics or Zululand or Transvaal or Natalia Republic or Orange Free State).ti,ab. | 42394 |
| 315 | or/24-314 [ALL LOW AND MIDDLE-INCOME COUNTRIES] | 1757962 |
| 316 | developing country/ or low income country/ | 80307 |
| 317 | ((developing or less* developed or under developed or underdeveloped or low* income) adj (economy or economies)).ti,ab. | 806 |
| 318 | ((developing or less* developed or under developed or underdeveloped or low* income or underserved or under served or deprived or poor*) adj (countr* or nation? or population? or world)).ti,ab. | 101624 |
| 319 | (low* adj (gdp or gnp or gross domestic or gross national)).ti,ab. | 338 |
| 320 | transitional countr*.ti,ab. | 176 |
| 321 | (third world or global south).ti,ab. | 4063 |
| 322 | North Korea/ | 289 |
| 323 | (North Korea or (Democratic People* Republic adj2 Korea)).ti,ab. | 507 |
| 324 | Haiti/ | 3544 |
| 325 | (Haiti or Hayti).ti,ab. | 3441 |
| 326 | Afghanistan/ | 3733 |
| 327 | Afghanistan.ti,ab. | 6876 |
| 328 | Nepal/ | 10445 |
| 329 | Nepal.ti,ab. | 12744 |
| 330 | Syrian Arab Republic/ | 0 |
| 331 | (Syria or Syrian Arab Republic).ti,ab. | 2618 |
| 332 | Yemen/ | 1555 |
| 333 | Yemen.ti,ab. | 2168 |
| 334 | Tajikistan/ | 802 |
| 335 | Tajikistan.ti,ab. | 738 |
| 336 | Benin/ | 1881 |
| 337 | (Benin or Dahomey).ti,ab. | 4045 |
| 338 | Burkina Faso/ | 3872 |
| 339 | (Burkina Faso or Burkina Fasso or Upper Volta).ti,ab. | 5105 |
| 340 | Burundi/ | 722 |
| 341 | (Burundi or Ruanda-Urundi).ti,ab. | 1092 |
| 342 | Central African Republic/ | 836 |
| 343 | (Central African Republic or Ubangi-Shari).ti,ab. | 1136 |
| 344 | Chad/ | 812 |
| 345 | Chad.ti,ab. | 1445 |
| 346 | Democratic Republic Congo/ | 0 |
| 347 | (((Democratic Republic or DR) adj2 Congo) or Congo-Kinshasa or Belgian Congo or Zaire or Congo Free State).ti,ab. | 5291 |
| 348 | Eritrea/ | 408 |
| 349 | Eritrea.ti,ab. | 667 |
| 350 | Ethiopia/ | 18406 |
| 351 | (Ethiopia or Abyssinia).ti,ab. | 25168 |
| 352 | Gambia/ | 2650 |
| 353 | Gambia.ti,ab. | 2628 |
| 354 | Guinea/ | 1281 |
| 355 | (Guinea not (New Guinea or Guinea Pig* or Guinea Fowl or Guinea-Bissau or Portuguese Guinea or Equatorial Guinea)).ti,ab. | 3170 |
| 356 | Guinea-Bissau/ | 1020 |
| 357 | (Guinea-Bissau or Portuguese Guinea).ti,ab. | 1154 |
| 358 | Liberia/ | 1365 |
| 359 | Liberia.ti,ab. | 1838 |
| 360 | Madagascar/ | 3872 |
| 361 | (Madagascar or Malagasy Republic).ti,ab. | 5524 |
| 362 | Malawi/ | 6571 |
| 363 | (Malawi or Nyasaland).ti,ab. | 8581 |
| 364 | Mali/ | 2658 |
| 365 | Mali.ti,ab. | 4155 |
| 366 | Mozambique/ | 2965 |
| 367 | (Mozambique or Mocambique or Portuguese East Africa).ti,ab. | 4432 |
| 368 | Niger/ | 1384 |
| 369 | (Niger not (Aspergillus or Peptococcus or Schizothorax or Cruciferae or Gobius or Lasius or Agelastes or Melanosuchus or radish or Parastromateus or Orius or Apergillus or Parastromateus or Stomoxys)).ti,ab. | 3991 |
| 370 | Rwanda/ | 3066 |
| 371 | (Rwanda or Ruanda).ti,ab. | 3943 |
| 372 | Sierra Leone/ | 1890 |
| 373 | (Sierra Leone or Salone).ti,ab. | 2765 |
| 374 | Somalia/ | 1842 |
| 375 | (Somalia or Somaliland).ti,ab. | 1879 |
| 376 | south sudan/ | 264 |
| 377 | South Sudan.ti,ab. | 746 |
| 378 | Tanzania/ | 13526 |
| 379 | (Tanzania or Tanganyika or Zanzibar).ti,ab. | 16462 |
| 380 | Togo/ | 1267 |
| 381 | (Togo or Togolese Republic or Togoland).ti,ab. | 1751 |
| 382 | Uganda/ | 14896 |
| 383 | Uganda.ti,ab. | 17908 |
| 384 | or/316-383 [ALL LOW INCOME COUNTRIES] | 301938 |
| 385 | 315 or 384 | 1757962 |
| 386 | 3 and 23 and 385 | 972 |
| 387 | limit 386 to (yr="2000 - 2023" and english) | 840 |

1. **Ovid Embase**

Search date: January 24, 2023

| No | Search strategies | Results |
| --- | --- | --- |
| 1 | exp health insurance/ or exp disability insurance/ | 318501 |
| 2 | (Health insurance* or medical aid or medical insurance* or universal health coverage or health fund* or health financ* or social health protection* or social health insurance* or community health fund* or community-based insurance* or health financing or (health adj3 insuranc*) or disability insuranc*).ti,ab. | 88894 |
| 3 | exp disabled person/ or exp disability/ | 256185 |
| 4 | ((disabilit* or disable* or handicap* or function* limitation* or function* diversit* or dependen* or special need* or rare disease* or incapacity* or impairment*) adj5 (person* or people or individ* or patient* or subject* or adult* or elderly or child* or boy* or girl* or kid* or m#n or wom#n or teenager* or juvenile or adolescent*)).ti,ab. | 383057 |
| 5 | exp physical disability/ or exp cerebral palsy/ or exp spinal dysraphism/ or exp muscular dystrophies/ or exp osteogenesis imperfecta/ or exp limb malformation/ or exp amputation/ or exp arm amputation/ or exp leg amputation/ or exp limb amputation/ or exp above knee amputation/ or exp below knee amputation/ or exp finger amputation/ or traumatic amputation/ or exp congenital amputation/ or exp foot amputation/ or hand amputation/ or exp knee amputation/ or exp thumb amputation/ or exp arthrogryposis/ or exp clubfoot/ or exp poliomyelitis/ or exp paralysis/ or exp hereditary motor sensory neuropathy/ or exp paraplegia/ or exp quadriplegia/ or exp spinal cord injury/ | 728180 |
| 6 | (Physical* adj3 (impair* or deficienc* or disable* or disabilit* or handicap* or incapacit*)).ti,ab. | 31524 |
| 7 | (Cerebral pals* or spina bifida or muscular distroph* or osteogenesis imperfecta or polio or poliomyelitis or paralyz* or paralys* or tetraplegi* or quadriplegi* or paraplegi* or hemiplegi* or wheelchair user* or wheel chair user* or amputation* or amputee*).ti,ab. | 287468 |
| 8 | exp hearing impairment/ | 129017 |
| 9 | ((deaf* or deaf-blind disorder* or ((hearing or acoustic) adj3 (loss* or impair* or deficienc* or disable* or disabilit* or handicap*))) not (blinding or double blind* or triple blind*)).ti,ab. | 126763 |
| 10 | exp visual disorder/ or exp blindness/ or exp visual impairment/ or visual deprivation/ or exp visual deficiency/ | 297755 |
| 11 | ((Blind* or ((visual* or vision) adj3 (loss* or disabilit* or disorder* or impairment* or disable* or deficienc* or handicap* or deprivation*))) not (blinding or double blind* or triple blind*)).ti,ab. | 315670 |
| 12 | exp speech disorder/ or exp communication disorder/ or exp language disability/ | 157717 |
| 13 | ((speech* or communication* or language) adj3 (disabilit* or disorder* or impair* or deficienc* or disable* or handicap*)).ti,ab. | 31167 |
| 14 | exp intellectual impairment/ or exp cognitive defect/ or exp developmental disorder/ or exp learning disorder/ or exp dementia/ or exp alzheimer disease/ | 855937 |
| 15 | (((intellectual or cognition or cognitive or learning or developmental) adj3 (disabilit* or disable* or impair* or disorder* or incapacit* or handicap* or dysfunction* or defect* or problem* or deficit* or difficult*)) or autism* or ADHD or attention deficit hyperactivity disorder* or attention-deficit hyperactivity disorder* or down syndrome or dementia or Alzheimer).ti,ab. | 628509 |
| 16 | exp mental deficiency/ or exp mental disease/ or exp mental patient/ | 2711459 |
| 17 | (((Mental* or psychological* or behavior*) adj3 (disabilit* or disable* or impair* or handicap* or incapacit* or disorder* or illness* or ill* or dysfunction* or retard* or deficienc* or disease* or diagnos*)) or psychosis or psychoses or schizoaffective or schizophreniform or schizophrenia or bipolar or mental health condition*).ti,ab. | 564375 |
| 18 | exp self help devices/ or exp sensory aid/ or exp orthopedic equipment/ or exp walking aid/ or exp rehabilitation equipment/ | 338326 |
| 19 | (Magnifier* or medical device* or ((assistive or mobilit*) adj3 (device* or technolog* or product* or equipment* or tool*)) or hearing aid* or wheelchair* or wheel chair* or orthotic* or prosthetic* or crutch* or walker* or walking aid or cane*).ti,ab. | 176745 |
| 20 | exp rehabilitation/ | 491197 |
| 21 | ((physical* or mental* or cognitive* or occupation* or speech* or voice* or vocation*) adj3 (rehabilitat* or therap*)).ti,ab. | 148487 |
| 22 | 1 or 2 | 350057 |
| 23 | 3 or 4 or 5 or 6 or 7 or 8 or 9 or 10 or 11 or 12 or 13 or 14 or 15 or 16 or 17 or 18 or 19 or 20 or 21 | 5264962 |
| 24 | developing country/ or low income country/ or middle income country/ | 124176 |
| 25 | ((developing or less* developed or under developed or underdeveloped or middle income or low* income) adj (economy or economies)).ti,ab. | 1046 |
| 26 | ((developing or less* developed or under developed or underdeveloped or middle income or low* income or underserved or under served or deprived or poor*) adj (countr* or nation? or population? or world)).ti,ab. | 164260 |
| 27 | (low* adj (gdp or gnp or gross domestic or gross national)).ti,ab. | 469 |
| 28 | (low adj3 middle adj3 countr*).ti,ab. | 32785 |
| 29 | (lmic or lmics or third world or lami countr*).ti,ab. | 14939 |
| 30 | transitional countr*.ti,ab. | 247 |
| 31 | global south.ti,ab. | 792 |
| 32 | "Africa south of the Sahara"/ | 17490 |
| 33 | ("Africa South of the Sahara" or sub-Saharan Africa or subSaharan Africa).ti,ab. | 32910 |
| 34 | Central Africa.ti,ab. | 4317 |
| 35 | Eastern Africa.ti,ab. | 1407 |
| 36 | Southern Africa.ti,ab. | 5647 |
| 37 | Western Africa.ti,ab. | 1071 |
| 38 | North Korea/ | 671 |
| 39 | (North Korea or (Democratic People* Republic adj2 Korea)).ti,ab. | 561 |
| 40 | Haiti/ | 4812 |
| 41 | (Haiti or Hayti).ti,ab. | 4432 |
| 42 | Afghanistan/ | 7107 |
| 43 | Afghanistan.ti,ab. | 8285 |
| 44 | Nepal/ | 15575 |
| 45 | Nepal.ti,ab. | 15412 |
| 46 | Syrian Arab Republic/ | 3421 |
| 47 | (Syria or Syrian Arab Republic).ti,ab. | 3205 |
| 48 | Yemen/ | 2442 |
| 49 | Yemen.ti,ab. | 2535 |
| 50 | Tajikistan/ | 1143 |
| 51 | Tajikistan.ti,ab. | 888 |
| 52 | Benin/ | 3156 |
| 53 | (Benin or Dahomey).ti,ab. | 5548 |
| 54 | Burkina Faso/ | 5352 |
| 55 | (Burkina Faso or Burkina Fasso or Upper Volta).ti,ab. | 6108 |
| 56 | Burundi/ | 1087 |
| 57 | (Burundi or Ruanda-Urundi).ti,ab. | 1220 |
| 58 | Central African Republic/ | 1064 |
| 59 | (Central African Republic or Ubangi-Shari).ti,ab. | 1271 |
| 60 | Chad/ | 1127 |
| 61 | Chad.ti,ab. | 1850 |
| 62 | Democratic Republic Congo/ | 5344 |
| 63 | (((Democratic Republic or DR) adj2 Congo) or Congo-Kinshasa or Belgian Congo or Zaire or Congo Free State).ti,ab. | 6310 |
| 64 | Eritrea/ | 713 |
| 65 | Eritrea.ti,ab. | 821 |
| 66 | Ethiopia/ | 25953 |
| 67 | (Ethiopia or Abyssinia).ti,ab. | 26080 |
| 68 | Gambia/ | 3230 |
| 69 | Gambia.ti,ab. | 2998 |
| 70 | Guinea/ | 3231 |
| 71 | (Guinea not (New Guinea or Guinea Pig* or Guinea Fowl or Guinea-Bissau or Portuguese Guinea or Equatorial Guinea)).ti,ab. | 3798 |
| 72 | Guinea-Bissau/ | 1247 |
| 73 | (Guinea-Bissau or Portuguese Guinea).ti,ab. | 1292 |
| 74 | Liberia/ | 2138 |
| 75 | Liberia.ti,ab. | 2170 |
| 76 | Madagascar/ | 5356 |
| 77 | (Madagascar or Malagasy Republic).ti,ab. | 6206 |
| 78 | Malawi/ | 9443 |
| 79 | (Malawi or Nyasaland).ti,ab. | 10048 |
| 80 | Mali/ | 4168 |
| 81 | Mali.ti,ab. | 5212 |
| 82 | Mozambique/ | 4766 |
| 83 | (Mozambique or Mocambique or Portuguese East Africa).ti,ab. | 5210 |
| 84 | Niger/ | 2933 |
| 85 | (Niger not (Aspergillus or Peptococcus or Schizothorax or Cruciferae or Gobius or Lasius or Agelastes or Melanosuchus or radish or Parastromateus or Orius or Apergillus or Parastromateus or Stomoxys)).ti,ab. | 4999 |
| 86 | Rwanda/ | 4754 |
| 87 | (Rwanda or Ruanda).ti,ab. | 4794 |
| 88 | Sierra Leone/ | 3029 |
| 89 | (Sierra Leone or Salone).ti,ab. | 3225 |
| 90 | Somalia/ | 2390 |
| 91 | (Somalia or Somaliland).ti,ab. | 2061 |
| 92 | south sudan/ | 501 |
| 93 | South Sudan.ti,ab. | 879 |
| 94 | Tanzania/ | 19092 |
| 95 | (Tanzania or Tanganyika or Zanzibar).ti,ab. | 19640 |
| 96 | Togo/ | 1768 |
| 97 | (Togo or Togolese Republic or Togoland).ti,ab. | 2020 |
| 98 | Uganda/ | 22860 |
| 99 | Uganda.ti,ab. | 22241 |
| 100 | Cambodia/ | 5953 |
| 101 | Cambodia.ti,ab. | 5749 |
| 102 | exp Indonesia/ | 25621 |
| 103 | (Indonesia or Dutch East Indies).ti,ab. | 26929 |
| 104 | kiribati/ | 163 |
| 105 | (Kiribati or Gilbert Islands or Phoenix Islands or Line Islands).ti,ab. | 297 |
| 106 | Laos/ | 2568 |
| 107 | (Laos or (Lao adj1 Democratic Republic)).ti,ab. | 2668 |
| 108 | exp "Federated States of Micronesia"/ | 1081 |
| 109 | Micronesia.ti,ab. | 785 |
| 110 | Mongolia/ | 3850 |
| 111 | Mongolia.ti,ab. | 6327 |
| 112 | Myanmar/ | 5257 |
| 113 | (Myanmar or Burma).ti,ab. | 6185 |
| 114 | Papua New Guinea/ | 7284 |
| 115 | (Papua New Guinea or German New Guinea or British New Guinea or Territory of Papua).ti,ab. | 5425 |
| 116 | Philippines/ | 14556 |
| 117 | (Philippines or Philippine Islands).ti,ab. | 13122 |
| 118 | solomon islands/ | 627 |
| 119 | Solomon Islands.ti,ab. | 1020 |
| 120 | Timor-Leste/ | 733 |
| 121 | (Timor-Leste or East Timor or Portuguese Timor).ti,ab. | 740 |
| 122 | Vanuatu/ | 553 |
| 123 | (Vanuatu or New Hebrides).ti,ab. | 866 |
| 124 | Viet Nam/ | 21026 |
| 125 | (Viet Nam or Vietnam or French Indochina).ti,ab. | 22778 |
| 126 | Kyrgyzstan/ | 1988 |
| 127 | (Kyrgyzstan or Kyrgyz Republic or Kirghizia or Kirghiz).ti,ab. | 1620 |
| 128 | Moldova/ | 1446 |
| 129 | Moldova.ti,ab. | 1186 |
| 130 | exp Ukraine/ | 19049 |
| 131 | Ukraine.ti,ab. | 9244 |
| 132 | exp Uzbekistan/ | 2864 |
| 133 | Uzbekistan.ti,ab. | 2152 |
| 134 | Bolivia/ | 4188 |
| 135 | Bolivia.ti,ab. | 4419 |
| 136 | El Salvador/ | 2479 |
| 137 | El Salvador.ti,ab. | 1891 |
| 138 | Honduras/ | 2401 |
| 139 | Honduras.ti,ab. | 2544 |
| 140 | Nicaragua/ | 2629 |
| 141 | Nicaragua.ti,ab. | 2626 |
| 142 | Djibouti/ | 423 |
| 143 | (Djibouti or French Somaliland).ti,ab. | 516 |
| 144 | Egypt/ | 25454 |
| 145 | Egypt.ti,ab. | 24035 |
| 146 | Morocco/ | 9678 |
| 147 | Morocco.ti,ab. | 9431 |
| 148 | Tunisia/ | 12159 |
| 149 | Tunisia.mp. | 14889 |
| 150 | palestine/ | 2144 |
| 151 | (Gaza or West Bank or Palestine).ti,ab. | 3856 |
| 152 | Bangladesh/ | 20965 |
| 153 | Bangladesh.ti,ab. | 21580 |
| 154 | Bhutan/ | 1116 |
| 155 | Bhutan.ti,ab. | 1140 |
| 156 | exp India/ | 188878 |
| 157 | India.ti,ab. | 177349 |
| 158 | exp Pakistan/ | 36292 |
| 159 | Pakistan.ti,ab. | 34380 |
| 160 | Angola/ | 1828 |
| 161 | Angola.ti,ab. | 1971 |
| 162 | Cameroon/ | 8788 |
| 163 | (Cameroon or Kamerun or Cameroun).ti,ab. | 9845 |
| 164 | Cape Verde/ | 499 |
| 165 | (Cape Verde or Cabo Verde).ti,ab. | 767 |
| 166 | Comoros/ | 427 |
| 167 | (Comoros or Glorioso Islands or Mayotte).ti,ab. | 738 |
| 168 | Congo/ | 4616 |
| 169 | (Congo not ((Democratic Republic adj3 Congo) or congo red or crimean-congo)).ti,ab. | 3888 |
| 170 | Cote d'Ivoire/ | 3772 |
| 171 | (Cote d'Ivoire or Cote dIvoire or Ivory Coast).ti,ab. | 5149 |
| 172 | eswatini/ | 300 |
| 173 | (eSwatini or Swaziland).ti,ab. | 1335 |
| 174 | Ghana/ | 15438 |
| 175 | (Ghana or Gold Coast).ti,ab. | 16701 |
| 176 | Kenya/ | 26279 |
| 177 | (Kenya or East Africa Protectorate).ti,ab. | 26051 |
| 178 | Lesotho/ | 945 |
| 179 | (Lesotho or Basutoland).ti,ab. | 1023 |
| 180 | Mauritania/ | 773 |
| 181 | Mauritania.ti,ab. | 803 |
| 182 | Nigeria/ | 46219 |
| 183 | Nigeria.ti,ab. | 44353 |
| 184 | "sao tome and principe"/ | 111 |
| 185 | (Sao Tome adj2 Principe).ti,ab. | 206 |
| 186 | Senegal/ | 7955 |
| 187 | Senegal.ti,ab. | 7917 |
| 188 | Sudan/ | 8463 |
| 189 | (Sudan not South Sudan).ti,ab. | 11373 |
| 190 | Zambia/ | 7928 |
| 191 | (Zambia or Northern Rhodesia).ti,ab. | 7750 |
| 192 | Zimbabwe/ | 8211 |
| 193 | (Zimbabwe or Southern Rhodesia).ti,ab. | 7220 |
| 194 | American Samoa/ | 335 |
| 195 | American Samoa.ti,ab. | 475 |
| 196 | china/ or guangxi/ or inner mongolia/ or macao/ or ningxia/ or tibet/ or xinjiang/ | 291651 |
| 197 | China.ti,ab. | 310884 |
| 198 | Fiji/ | 2092 |
| 199 | Fiji.ti,ab. | 2954 |
| 200 | exp Malaysia/ | 29439 |
| 201 | (Malaysia or Malayan Union or Malaya).ti,ab. | 29401 |
| 202 | marshall islands/ | 199 |
| 203 | Marshall Islands.ti,ab. | 393 |
| 204 | nauru/ | 92 |
| 205 | Nauru.ti,ab. | 188 |
| 206 | Samoa/ | 764 |
| 207 | ((Samoa not American Samoa) or Western Samoa or Navigator Islands or Samoan Islands).ti,ab. | 763 |
| 208 | Thailand/ | 40960 |
| 209 | (Thailand or Siam).ti,ab. | 39918 |
| 210 | Tonga/ | 448 |
| 211 | Tonga.ti,ab. | 560 |
| 212 | tuvalu/ | 66 |
| 213 | (Tuvalu or Ellice Islands).ti,ab. | 86 |
| 214 | Albania/ | 2191 |
| 215 | Albania.ti,ab. | 2043 |
| 216 | Armenia/ | 2427 |
| 217 | Armenia.ti,ab. | 2046 |
| 218 | exp Azerbaijan/ | 2168 |
| 219 | Azerbaijan.ti,ab. | 2401 |
| 220 | Belarus/ | 3219 |
| 221 | (Belarus or Byelarus or Byelorussia or Belorussia).ti,ab. | 2855 |
| 222 | exp "Bosnia and Herzegovina"/ | 3191 |
| 223 | (Bosnia or Herzegovina).ti,ab. | 3436 |
| 224 | Bulgaria/ | 10896 |
| 225 | Bulgaria.ti,ab. | 7956 |
| 226 | exp "Georgia (republic)"/ | 2300 |
| 227 | Georgia.ti,ab. not "georgia (u.s.)"/ | 13521 |
| 228 | Kazakhstan/ | 4967 |
| 229 | (Kazakhstan or Kazakh).ti,ab. | 5335 |
| 230 | Kosovo/ | 733 |
| 231 | Kosovo.ti,ab. | 1485 |
| 232 | "Montenegro (republic)"/ | 907 |
| 233 | Montenegro.ti,ab. | 1368 |
| 234 | "republic of north macedonia"/ | 176 |
| 235 | North Macedonia.ti,ab. | 282 |
| 236 | Romania/ | 16653 |
| 237 | Romania.ti,ab. | 11679 |
| 238 | exp Russian Federation/ | 71392 |
| 239 | ussr/ | 48332 |
| 240 | (Russia or Russian Federation or USSR or Union of Soviet Socialist Republics or Soviet Union).ti,ab. | 45052 |
| 241 | exp Serbia/ | 7141 |
| 242 | Serbia.ti,ab. | 8280 |
| 243 | "Turkey (republic)"/ | 43522 |
| 244 | (Turkey.ti,ab. not "Turkey (bird)"/) or (Anatolia or Asia Minor).ti,ab. | 52453 |
| 245 | Turkmenistan/ | 692 |
| 246 | Turkmenistan.ti,ab. | 458 |
| 247 | Argentina/ | 27039 |
| 248 | (Argentina or Argentine Republic).ti,ab. | 27160 |
| 249 | Belize/ | 902 |
| 250 | (Belize or British Honduras).ti,ab. | 1099 |
| 251 | exp Brazil/ | 142875 |
| 252 | Brazil.ti,ab. | 128013 |
| 253 | Colombia/ | 25560 |
| 254 | Colombia.ti,ab. | 22497 |
| 255 | Costa Rica/ | 5649 |
| 256 | Costa Rica.ti,ab. | 6050 |
| 257 | Cuba/ | 7749 |
| 258 | Cuba.ti,ab. | 6882 |
| 259 | Dominica/ | 240 |
| 260 | Dominica.ti,ab. | 618 |
| 261 | Dominican Republic/ | 3099 |
| 262 | Dominican Republic.ti,ab. | 2916 |
| 263 | Ecuador/ | 7207 |
| 264 | Ecuador.ti,ab. | 7432 |
| 265 | Grenada/ | 347 |
| 266 | Grenada.ti,ab. | 455 |
| 267 | Guatemala/ | 5390 |
| 268 | Guatemala.ti,ab. | 5163 |
| 269 | Guyana/ | 1229 |
| 270 | (Guyana or British Guiana).ti,ab. | 1588 |
| 271 | Jamaica/ | 4769 |
| 272 | Jamaica.ti,ab. | 4441 |
| 273 | exp Mexico/ | 57487 |
| 274 | (Mexico or United Mexican States).ti,ab. | 63040 |
| 275 | Paraguay/ | 1878 |
| 276 | Paraguay.mp. | 2767 |
| 277 | Peru/ | 16012 |
| 278 | Peru.ti,ab. | 17131 |
| 279 | Saint Lucia/ | 153 |
| 280 | (St Lucia or Saint Lucia or Iyonala or Hewanorra).ti,ab. | 433 |
| 281 | "Saint Vincent and the Grenadines"/ | 92 |
| 282 | (Saint Vincent or St Vincent or Grenadines).ti,ab. | 991 |
| 283 | Suriname/ | 1418 |
| 284 | (Suriname or Dutch Guiana).ti,ab. | 889 |
| 285 | Venezuela/ | 7408 |
| 286 | Venezuela.ti,ab. | 7280 |
| 287 | Algeria/ | 6108 |
| 288 | Algeria.ti,ab. | 6256 |
| 289 | Iran/ | 63143 |
| 290 | (Iran or Persia).ti,ab. | 67584 |
| 291 | exp Iraq/ | 12255 |
| 292 | (Iraq or Mesopotamia).ti,ab. | 14390 |
| 293 | Jordan/ | 8849 |
| 294 | Jordan.ti,ab. | 10618 |
| 295 | Lebanon/ | 7511 |
| 296 | (Lebanon or Lebanese Republic).ti,ab. | 7297 |
| 297 | Libyan Arab Jamahiriya/ | 2009 |
| 298 | libya.ti,ab. | 1838 |
| 299 | maldives/ | 437 |
| 300 | Maldives.ti,ab. | 512 |
| 301 | Sri Lanka/ | 10642 |
| 302 | (Sri Lanka or Ceylon).ti,ab. | 10707 |
| 303 | Botswana/ | 3321 |
| 304 | (Botswana or Bechuanaland or Kalahari).ti,ab. | 3684 |
| 305 | Equatorial Guinea/ | 557 |
| 306 | (Equatorial Guinea or Spanish Guinea).ti,ab. | 650 |
| 307 | Gabon/ | 2049 |
| 308 | (Gabon or Gabonese Republic).ti,ab. | 2280 |
| 309 | Mauritius/ | 1073 |
| 310 | (Mauritius or Agalega Islands).ti,ab. | 1244 |
| 311 | Namibia/ | 2016 |
| 312 | (Namibia or German South West Africa).ti,ab. | 2123 |
| 313 | South Africa/ | 61518 |
| 314 | (South Africa or Cape Colony or British Bechuanaland or Boer Republics or Zululand or Transvaal or Natalia Republic or Orange Free State).ti,ab. | 51144 |
| 315 | or/24-314 [ALL LOW AND MIDDLE-INCOME COUNTRIES] | 2175258 |
| 316 | developing country/ or low income country/ | 114364 |
| 317 | ((developing or less* developed or under developed or underdeveloped or low* income) adj (economy or economies)).ti,ab. | 915 |
| 318 | ((developing or less* developed or under developed or underdeveloped or low* income or underserved or under served or deprived or poor*) adj (countr* or nation? or population? or world)).ti,ab. | 131841 |
| 319 | (low* adj (gdp or gnp or gross domestic or gross national)).ti,ab. | 469 |
| 320 | transitional countr*.ti,ab. | 247 |
| 321 | (third world or global south).ti,ab. | 4563 |
| 322 | North Korea/ | 671 |
| 323 | (North Korea or (Democratic People* Republic adj2 Korea)).ti,ab. | 561 |
| 324 | Haiti/ | 4812 |
| 325 | (Haiti or Hayti).ti,ab. | 4432 |
| 326 | Afghanistan/ | 7107 |
| 327 | Afghanistan.ti,ab. | 8285 |
| 328 | Nepal/ | 15575 |
| 329 | Nepal.ti,ab. | 15412 |
| 330 | Syrian Arab Republic/ | 3421 |
| 331 | (Syria or Syrian Arab Republic).ti,ab. | 3205 |
| 332 | Yemen/ | 2442 |
| 333 | Yemen.ti,ab. | 2535 |
| 334 | Tajikistan/ | 1143 |
| 335 | Tajikistan.ti,ab. | 888 |
| 336 | Benin/ | 3156 |
| 337 | (Benin or Dahomey).ti,ab. | 5548 |
| 338 | Burkina Faso/ | 5352 |
| 339 | (Burkina Faso or Burkina Fasso or Upper Volta).ti,ab. | 6108 |
| 340 | Burundi/ | 1087 |
| 341 | (Burundi or Ruanda-Urundi).ti,ab. | 1220 |
| 342 | Central African Republic/ | 1064 |
| 343 | (Central African Republic or Ubangi-Shari).ti,ab. | 1271 |
| 344 | Chad/ | 1127 |
| 345 | Chad.ti,ab. | 1850 |
| 346 | Democratic Republic Congo/ | 5344 |
| 347 | (((Democratic Republic or DR) adj2 Congo) or Congo-Kinshasa or Belgian Congo or Zaire or Congo Free State).ti,ab. | 6310 |
| 348 | Eritrea/ | 713 |
| 349 | Eritrea.ti,ab. | 821 |
| 350 | Ethiopia/ | 25953 |
| 351 | (Ethiopia or Abyssinia).ti,ab. | 26080 |
| 352 | Gambia/ | 3230 |
| 353 | Gambia.ti,ab. | 2998 |
| 354 | Guinea/ | 3231 |
| 355 | (Guinea not (New Guinea or Guinea Pig* or Guinea Fowl or Guinea-Bissau or Portuguese Guinea or Equatorial Guinea)).ti,ab. | 3798 |
| 356 | Guinea-Bissau/ | 1247 |
| 357 | (Guinea-Bissau or Portuguese Guinea).ti,ab. | 1292 |
| 358 | Liberia/ | 2138 |
| 359 | Liberia.ti,ab. | 2170 |
| 360 | Madagascar/ | 5356 |
| 361 | (Madagascar or Malagasy Republic).ti,ab. | 6206 |
| 362 | Malawi/ | 9443 |
| 363 | (Malawi or Nyasaland).ti,ab. | 10048 |
| 364 | Mali/ | 4168 |
| 365 | Mali.ti,ab. | 5212 |
| 366 | Mozambique/ | 4766 |
| 367 | (Mozambique or Mocambique or Portuguese East Africa).ti,ab. | 5210 |
| 368 | Niger/ | 2933 |
| 369 | (Niger not (Aspergillus or Peptococcus or Schizothorax or Cruciferae or Gobius or Lasius or Agelastes or Melanosuchus or radish or Parastromateus or Orius or Apergillus or Parastromateus or Stomoxys)).ti,ab. | 4999 |
| 370 | Rwanda/ | 4754 |
| 371 | (Rwanda or Ruanda).ti,ab. | 4794 |
| 372 | Sierra Leone/ | 3029 |
| 373 | (Sierra Leone or Salone).ti,ab. | 3225 |
| 374 | Somalia/ | 2390 |
| 375 | (Somalia or Somaliland).ti,ab. | 2061 |
| 376 | south sudan/ | 501 |
| 377 | South Sudan.ti,ab. | 879 |
| 378 | Tanzania/ | 19092 |
| 379 | (Tanzania or Tanganyika or Zanzibar).ti,ab. | 19640 |
| 380 | Togo/ | 1768 |
| 381 | (Togo or Togolese Republic or Togoland).ti,ab. | 2020 |
| 382 | Uganda/ | 22860 |
| 383 | Uganda.ti,ab. | 22241 |
| 384 | or/316-383 [ALL LOW INCOME COUNTRIES] | 373283 |
| 385 | 315 or 384 | 2175258 |
| 386 | 22 and 23 and 385 | 3218 |
| 387 | limit 386 to (english language and yr="2000 - 2023") | 3003 |

1. **Ovid CINAHL**

Search date: January 24, 2023

| No | Search strategies | Results |
| --- | --- | --- |
|  | **Health Insurance** |  |
| 1 | Insurance, Health OR Health Insurance Exchanges OR Insurance, Health, Reimbursement OR community-based health insurance OR | 56,045 |
| 2 | “Health insurance*” OR “disability insurance*” OR “medical aid” OR “medical insurance*” OR “universal health coverage” OR “health fund*” OR “health financ*” OR social health protection* OR “social health insurance*” OR community health fund* OR “community-based insurance*” OR “community based insurance” OR health financing OR (health N3 insurance*) OR (disabilit* N3 insurance*) | 83,975 |
|  | **People with disabilities** |  |
| 3 | Health services for persons with disabilities OR assistive technology devices OR intellectual disability OR students with disabilities OR children with disabilities OR communication aids for persons with disabilities OR persons with mental disabilities OR persons with disabilities OR deaf-blind disorders OR hearing loss, functional OR hearing loss, partial OR hearing loss, sensorineural OR hearing loss, noise-induced OR hearing loss, high-frequency OR hearing loss, conductive OR hearing loss, central OR deafness OR | 124,703 |
| 4 | ((person* OR people OR individ* OR patient* OR subject* OR adult* OR elderly OR child* OR boy* OR girl* OR kid* OR m?n OR wom?n OR teenager* OR juvenile OR adolescent* OR body OR bodies) N5 (disabilit* OR disable* OR disabl* OR handicap* OR “function* limitation*” OR “function* diversit*” OR dependen* OR “special need*” OR “rare disease*” OR incapacit* OR impairment*)) | 181,211 |
|  | **Physical disabilities** |  |
| 5 | “physical disabilit*” OR “cerebral pals*” OR “spinal dysraphism” OR “muscular dystroph*” OR “rheumatoid arthritis” OR “osteogenesis imperfecta” OR “limb malformation*”OR amputat* OR amputee* OR “arm amputation*” OR “leg amputation*” OR “limb amputation*” OR “above knee amputation*” OR “below knee amputation*” OR “finger amputation*” OR “traumatic amputation*” OR “congenital amputation*” OR “foot amputation*”OR “hand amputation*” OR “knee amputation*” OR “thumb amputation*” OR arthrogryposis OR clubfoot OR “congenital hip dislocation*” OR poliomyelitis OR paralys* OR “hereditary motor sensory neuropathy” OR parapleg* OR quadripleg* OR tetrapleg* OR hemipleg* OR paralyz* OR “spinal cord injur*” OR “spina bifida” OR “muscular distroph*” OR polio OR “wheelchair user*” OR “wheel chair user*” | 139,727 |
| 6 | (Physical* N3 (impair* OR deficienc* OR disable* OR disabilit* OR handicap* OR incapacit* OR defect*)) | 13,021 |
|  | **Hearing disabilities** |  |
| 7 | deaf* OR “deaf-blind disorder*” OR ((hearing or acoustic) N3 (loss* OR impair* OR deficienc* OR disable* OR disabilit* OR handicap* OR defect*)) | 67,571 |
|  | **Visual disabilities** |  |
| 8 | Blind* OR ((visual* OR vision) N3 (loss* OR disabilit* OR disorder* OR impairment* OR disable* OR deficienc* OR handicap* OR deprivation* OR defect*)) | 174,369 |
|  | **Speech disabilities** |  |
| 9 | ((speech* OR communication* OR language) N3 (disabilit* OR disorder* OR impair* OR deficienc* OR disable* OR handicap*)) | 18,500 |
|  | **Intellectual disabilities** |  |
| 10 | ((intellectual OR cognition OR cognitive OR learning OR developmental) N3 (disabilit* OR disable* OR impair* OR disorder* OR incapacit* OR handicap* OR dysfunction* OR defect* OR problem* OR deficit* OR difficult*)) OR autism* OR ADHD OR “attention deficit hyperactivity disorder*” OR “attention-deficit hyperactivity disorder*” OR “down syndrome*” OR dementia OR Alzheimer | 274,831 |
|  | **Mental disabilities** |  |
| 11 | ((Mental* OR psychological* OR behavior*) N3 (disabilit* OR disable* OR impair* OR handicap* OR incapacit* OR disorder* OR illness* OR ill* OR dysfunction* OR retard* OR deficienc* OR disease* OR diagnos*)) OR psychosis OR psychoses OR schizoaffective OR schizophreniform OR schizophrenia OR bipolar OR “mental health condition*” | 207,817 |
|  | **Assistive devices/technology** |  |
| 12 | “Self-help device*” OR “self help device*” OR “sensory aid” OR “orthopedic equipment*” OR wheelchair* OR “wheel chair*” OR crutch* OR walker* OR orthotic* OR prosthetic* OR “walking aid” OR cane* OR “hearing aid” ((assistive OR mobilit*) N3 (device* OR technolog* OR product* OR equipment* OR tool*)) | 74,896 |
|  | **Rehabilitation** |  |
| 13 | Rehabilitation* OR ((physical* OR mental* OR cognitive* OR occupation* OR speech* OR voice* OR vocation*) N3 (rehabilitat* OR therap*)) | 349,179 |
|  | DISABILITIES |  |
| 14 | **#3 OR #4 OR #5 OR #6 OR #7 OR #8 OR #9 OR #10 OR #11 OR #12** | 1,152,249 |
|  | Low-income countries (LICs) & LMICs |  |
| 15 | “Global south” OR "lower- and middle-income countr*" OR “low- and middle income countr*” LMIC* OR "low-income countr*" OR "low income countr*" OR LIC OR LICs OR (developing OR “low income” OR underdeveloped OR poor OR “low resource*” OR “less developed” OR “under-developed”) N3 (countr* OR nation* OR world* OR setting* OR population*) | 51,718 |
|  | LICs |  |
| 16 | "Democratic People's Republic of Korea" OR Afghanistan OR Syria OR Yemen OR “Burkina Faso” OR Burundi OR “Central African Republic” OR Chad OR "Democratic Republic of The Congo" OR Congo OR Eritrea OR Ethiopia OR Gambia OR Guinea OR “Guinea-Bisau” OR Liberia OR Madagascar OR Malawi OR Mali OR Mozambique OR Niger OR Rwanda OR Sierra Leone OR Somalia OR “South Sudan” OR Sudan OR Togo OR Uganda | 50,759 |
|  | LMICs |  |
| 17 | “Central Africa” OR “Eastern Africa” OR “Southern Africa” OR “Western Africa” OR Cambodia OR Indonesia OR Kiribati OR Laos OR Lao OR Micronesia OR Mongolia OR Myanmar OR Burma OR “Papua New Guinea” OR Philippines OR Samoa OR “Solomon Islands” OR “Timor Leste” OR “Timor-Leste” OR Vanuatu OR Vietnam OR “Viet Nam” OR “American Samoa” OR China OR Fiji OR Malaysia OR “Marshall Islands” OR Thailand OR Tonga OR Tuvalu OR Kyrgyzstan OR Tajikistan OR Ukraine OR Uzbekistan OR Albania OR Armenia OR Azerbaijan OR Belarus OR “Bosnia-Herzegovina” OR Bulgaria OR Georgia OR Kazakhstan OR Kosovo OR Moldova OR Montenegro OR Macedonia OR Romania OR Russia OR Serbia OR Turkey OR Turkmenistan OR Belize OR Bolivia OR El Salvador OR Haiti OR Honduras Or Nicaragua OR Argentina OR Brazil OR Colombia OR Costa Rica OR Cuba OR Dominica OR Dominican Republic OR Ecuador OR Grenada OR Guatemala OR Guyana OR Jamaica OR Mexico OR Panama OR Paraguay OR Peru OR “Saint Lucia” OR “St Lucia” OR Suriname OR Venezuela OR Algeria OR Djibouti OR Egypt OR Iran OR Morocco OR Tunisia OR Palestine OR Gaza OR Iraq OR Jordan OR Lebanon OR Libya OR Afghanistan OR Bangladesh OR Bhutan OR India OR Nepal OR Pakistan OR “Sri Lanka” OR Maldives OR Angola OR Benin OR Cameroon OR Cape Verde OR Comoros OR Congo OR “Cote d'Ivoire” OR “Ivory Coast” OR Eswatini OR Ghana OR Kenya OR Lesotho OR Mauritania OR Nigeria OR Senegal OR Tanzania OR Zambia OR Zimbabwe OR Botswana OR Equatorial Guinea OR Gabon OR Mauritius OR Namibia OR “South Africa” OR Syria OR Yemen OR “Burkina Faso” OR Burundi OR “Central African Republic” OR Chad OR "Democratic Republic of the Congo" OR Eritrea OR Ethiopia OR Gambia OR Guinea OR “Guinea-Bisau” OR Liberia OR Madagascar OR Malawi OR Mali OR Mozambique OR Niger OR Rwanda OR Sierra Leone OR Somalia OR South Sudan OR Sudan OR Togo OR Uganda | 531,152 |
| 18 | **#17 OR #16 OR #15 (LICs OR LMICs)** | 559,650 |
| 19 | **#2 OR #1 (Health insurance)** | 83,975 |
| 18 | **#14 AND #18 AND #19** | 529 |
| 19 | **#14 AND #18 AND #19**  Filter   - timeframe 2000 – 2023 - Language: English | 504 |

1. **Web of Science**

Search date: January 24, 2023

| No | Search strategies |  |
| --- | --- | --- |
|  | Health Insurance |  |
| 1 | **((TS=( “Health insurance*” OR “disability insurance*” OR “medical aid” OR “medical insurance*” OR “universal health coverage” OR “health fund*” OR “health financ*” OR social health protection* OR “social health insurance*” OR community health fund* OR “community-based insurance*” OR “community based insurance” OR health financing OR (health NEAR/3 insurance) )) OR TI=(“Health insurance*” OR “disability insurance*” OR “medical aid” OR “medical insurance*” OR “universal health coverage” OR “health fund*” OR “health financ*” OR social health protection* OR “social health insurance*” OR community health fund* OR “community-based insurance*” OR “community based insurance” OR health financing OR (health NEAR/3 insurance))) OR AB=(“Health insurance*” OR “disability insurance*” OR “medical aid” OR “medical insurance*” OR “universal health coverage” OR “health fund*” OR “health financ*” OR social health protection* OR “social health insurance*” OR community health fund* OR “community-based insurance*” OR “community based insurance” OR health financing OR (health NEAR/3 insurance))** | [103,136](https://www.webofscience.com/wos/woscc/summary/f203cb40-641f-4828-8b78-23db0640a420-6c00545d/relevance/1) |
|  | People with disabilities |  |
| 2 | **((TS=( disabilit* OR disabled OR impairment* OR impair* OR handicap* )) OR TI=(disabilit* OR disabled OR impairment* OR impair* OR handicap*)) OR AB=(disabilit* OR disabled OR impairment* OR impair* OR handicap*)** | [1,287,020](https://www.webofscience.com/wos/woscc/summary/79b32072-5468-48e9-a0c6-bcda84e9899a-6c00615b/relevance/1) |
| 3 | **((TS=(( (person* OR people OR individ* OR patient* OR subject* OR adult* OR elderly OR child* OR boy* OR girl* OR kid* OR m?n OR wom?n OR teenager* OR juvenile OR adolescent* OR body OR bodies) NEAR/5 (disabilit* OR disable* OR disabl* OR handicap* OR “function* limitation*” OR “function* diversit*” OR dependen* OR “special need*” OR “rare disease*” OR incapacit* OR impairment*) ))) OR TI=(((person* OR people OR individ* OR patient* OR subject* OR adult* OR elderly OR child* OR boy* OR girl* OR kid* OR m?n OR wom?n OR teenager* OR juvenile OR adolescent* OR body OR bodies) NEAR/5 (disabilit* OR disable* OR disabl* OR handicap* OR “function* limitation*” OR “function* diversit*” OR dependen* OR “special need*” OR “rare disease*” OR incapacit* OR impairment*)))) OR AB=(((person* OR people OR individ* OR patient* OR subject* OR adult* OR elderly OR child* OR boy* OR girl* OR kid* OR m?n OR wom?n OR teenager* OR juvenile OR adolescent* OR body OR bodies) NEAR/5 (disabilit* OR disable* OR disabl* OR handicap* OR “function* limitation*” OR “function* diversit*” OR dependen* OR “special need*” OR “rare disease*” OR incapacit* OR impairment*)))** | [357,099](https://www.webofscience.com/wos/woscc/summary/f2c1e962-c742-40df-bd81-4d9b3cd9e535-6c00794d/relevance/1) |
|  | Physical disabilities |  |
| 4 | **((TS=( “physical disabilit*” OR “cerebral pals*” OR “spinal dysraphism” OR “muscular dystroph*” OR “rheumatoid arthritis” OR “osteogenesis imperfecta” OR “limb malformation*”OR amputation* OR amputee* OR “arm amputation*” OR “leg amputation*” OR “limb amputation*” OR “above knee amputation*” OR “below knee amputation*” OR “finger amputation*” OR “traumatic amputation*” OR “congenital amputation*” OR “foot amputation*”OR “hand amputation*” OR “knee amputation*” OR “thumb amputation*” OR arthrogryposis OR clubfoot OR poliomyelitis OR paralys* OR “hereditary motor sensory neuropathy” OR parapleg* OR quadripleg* OR tetrapleg* OR hemipleg* OR paralyz* OR “spinal cord injur*” OR “spina bifida” OR “muscular distroph*” OR polio OR “wheelchair user*” OR “wheel chair user*”)) OR TI=( “physical disabilit*” OR “cerebral pals*” OR “spinal dysraphism” OR “muscular dystroph*” OR “rheumatoid arthritis” OR “osteogenesis imperfecta” OR “limb malformation*”OR amputation* OR amputee* OR “arm amputation*” OR “leg amputation*” OR “limb amputation*” OR “above knee amputation*” OR “below knee amputation*” OR “finger amputation*” OR “traumatic amputation*” OR “congenital amputation*” OR “foot amputation*”OR “hand amputation*” OR “knee amputation*” OR “thumb amputation*” OR arthrogryposis OR clubfoot OR poliomyelitis OR paralys* OR “hereditary motor sensory neuropathy” OR parapleg* OR quadripleg* OR tetrapleg* OR hemipleg* OR paralyz* OR “spinal cord injur*” OR “spina bifida” OR “muscular distroph*” OR polio OR “wheelchair user*” OR “wheel chair user*”)) OR AB=( “physical disabilit*” OR “cerebral pals*” OR “spinal dysraphism” OR “muscular dystroph*” OR “rheumatoid arthritis” OR “osteogenesis imperfecta” OR “limb malformation*”OR amputation* OR amputee* OR “arm amputation*” OR “leg amputation*” OR “limb amputation*” OR “above knee amputation*” OR “below knee amputation*” OR “finger amputation*” OR “traumatic amputation*” OR “congenital amputation*” OR “foot amputation*”OR “hand amputation*” OR “knee amputation*” OR “thumb amputation*” OR arthrogryposis OR clubfoot OR poliomyelitis OR paralys* OR “hereditary motor sensory neuropathy” OR parapleg* OR quadripleg* OR tetrapleg* OR hemipleg* OR paralyz* OR “spinal cord injur*” OR “spina bifida” OR “muscular distroph*” OR polio OR “wheelchair user*” OR “wheel chair user*”)** | [475,161](https://www.webofscience.com/wos/woscc/summary/8e9d0364-7006-4761-b620-fc0f7e1e443d-6c01a84b/relevance/1) |
| 5 | **((TS=((Physical* NEAR/3 (impair* OR deficienc* OR disable* OR disabilit* OR handicap* OR incapacit* OR defect*)))) OR TI=((Physical* NEAR/3 (impair* OR deficienc* OR disable* OR disabilit* OR handicap* OR incapacit* OR defect*)))) OR AB=((Physical* NEAR/3 (impair* OR deficienc* OR disable* OR disabilit* OR handicap* OR incapacit* OR defect*)))** | [30,067](https://www.webofscience.com/wos/woscc/summary/1f7aee97-2e32-421e-b89d-0264c59ae49e-6c01abe9/relevance/1) |
|  | Hearing disabilities |  |
| 6 | **(TS=(deaf* OR “deaf-blind disorder*” OR ((hearing or acoustic) NEAR/3 (loss* OR impair* OR deficienc* OR disable* OR disabilit* OR handicap* OR defect*)))) NOT TS=((blinding OR “double blind*” OR “triple blind*”))** | [105,828](https://www.webofscience.com/wos/woscc/summary/b90a0378-0ea4-4722-b488-ec8947e4b405-6c011453/relevance/1) |
| 7 | **(TI=(deaf* OR “deaf-blind disorder*” OR ((hearing or acoustic) NEAR/3 (loss* OR impair* OR deficienc* OR disable* OR disabilit* OR handicap* OR defect*)))) NOT TI=((blinding OR “double blind*” OR “triple blind*”))** | [44,752](https://www.webofscience.com/wos/woscc/summary/901ca98b-61f3-4f3c-991b-bae504457beb-6c01238a/relevance/1) |
| 8 | **(AB=(deaf* OR “deaf-blind disorder*” OR ((hearing or acoustic) NEAR/3 (loss* OR impair* OR deficienc* OR disable* OR disabilit* OR handicap* OR defect*)))) NOT AB=((blinding OR “double blind*” OR “triple blind*”))** | [72,906](https://www.webofscience.com/wos/woscc/summary/1ae2e222-dc2d-4226-b161-463450308621-6c012d4a/relevance/1) |
|  | Visual disabilities |  |
| 9 | **(TS=(Blind* OR ((visual* OR vision) NEAR/3 (loss* OR disabilit* OR disorder* OR impairment* OR disable* OR deficienc* OR handicap* OR deprivation* OR defect*)))) NOT TS=((blinding OR “double blind*” OR “triple blind*”))** | [109,303](https://www.webofscience.com/wos/woscc/summary/04e047be-1351-459c-b1f5-0a2e364a0fc9-6c0149d1/relevance/1) |
| 10 | **(TI=(Blind* OR ((visual* OR vision) NEAR/3 (loss* OR disabilit* OR disorder* OR impairment* OR disable* OR deficienc* OR handicap* OR deprivation* OR defect*)))) NOT TI=((blinding OR “double blind*” OR “triple blind*”))** | [25,966](https://www.webofscience.com/wos/woscc/summary/44ce776f-ca95-4237-a8df-7faabfdf902a-6c015648/relevance/1) |
| 11 | **(AB=(Blind* OR ((visual* OR vision) NEAR/3 (loss* OR disabilit* OR disorder* OR impairment* OR disable* OR deficienc* OR handicap* OR deprivation* OR defect*)))) NOT AB=((blinding OR “double blind*” OR “triple blind*”))** | [85,985](https://www.webofscience.com/wos/woscc/summary/958e21a9-35a8-4045-8e25-9fe9d3da90df-6c015e6c/relevance/1) |
|  | Speech disabilities |  |
| 12 | **((TS=(((speech* OR communication* OR language) NEAR/3 (disabilit* OR disorder* OR impair* OR deficienc* OR disable* OR handicap*)))) OR TI=(((speech* OR communication* OR language) NEAR/5 (disabilit* OR disorder* OR impair* OR deficienc* OR disable* OR handicap*)))) OR AB=(((speech* OR communication* OR language) NEAR/3 (disabilit* OR disorder* OR impair* OR deficienc* OR disable* OR handicap*)))** | [35,021](https://www.webofscience.com/wos/woscc/summary/b05a3f86-db74-4539-9e68-f413ab1b1840-6c01698f/relevance/1) |
|  | Intellectual disabilities |  |
| 13 | **((TS=(((intellectual OR cognition OR cognitive OR learning OR developmental) NEAR/3 (disabilit* OR disable* OR impair* OR disorder* OR incapacit* OR handicap* OR dysfunction* OR defect* OR problem* OR deficit* OR difficult*)) OR autism* OR ADHD OR “attention deficit hyperactivity disorder*” OR “attention-deficit hyperactivity disorder*” OR “down syndrome*” OR dementia OR Alzheimer)) OR TI=(((intellectual OR cognition OR cognitive OR learning OR developmental) NEAR/3 (disabilit* OR disable* OR impair* OR disorder* OR incapacit* OR handicap* OR dysfunction* OR defect* OR problem* OR deficit* OR difficult*)) OR autism* OR ADHD OR “attention deficit hyperactivity disorder*” OR “attention-deficit hyperactivity disorder*” OR “down syndrome*” OR dementia OR Alzheimer)) OR AB=(((intellectual OR cognition OR cognitive OR learning OR developmental) NEAR/3 (disabilit* OR disable* OR impair* OR disorder* OR incapacit* OR handicap* OR dysfunction* OR defect* OR problem* OR deficit* OR difficult*)) OR autism* OR ADHD OR “attention deficit hyperactivity disorder*” OR “attention-deficit hyperactivity disorder*” OR “down syndrome*” OR dementia OR Alzheimer)** | [755,446](https://www.webofscience.com/wos/woscc/summary/7ee3eb08-71f7-4a04-821e-d0d2c0366038-6c016e5d/relevance/1) |
|  | Mental disabilities |  |
| 14 | **((TS=(((Mental* OR psychological* OR behavio$r*) NEAR/3 (disabilit* OR disable* OR impair* OR handicap* OR incapacit* OR disorder* OR illness* OR ill* OR dysfunction* OR retard* OR deficienc* OR disease* OR diagnos*)) OR psychosis OR psychoses OR schizoaffective OR schizophreniform OR schizophrenia OR bipolar OR “mental health condition*”)) OR TI=(((Mental* OR psychological* OR behavio$r*) NEAR/3 (disabilit* OR disable* OR impair* OR handicap* OR incapacit* OR disorder* OR illness* OR ill* OR dysfunction* OR retard* OR deficienc* OR disease* OR diagnos*)) OR psychosis OR psychoses OR schizoaffective OR schizophreniform OR schizophrenia OR bipolar OR “mental health condition*”)) OR AB=(((Mental* OR psychological* OR behavio$r*) NEAR/3 (disabilit* OR disable* OR impair* OR handicap* OR incapacit* OR disorder* OR illness* OR ill* OR dysfunction* OR retard* OR deficienc* OR disease* OR diagnos*)) OR psychosis OR psychoses OR schizoaffective OR schizophreniform OR schizophrenia OR bipolar OR “mental health condition*”)** | [[617,090](https://www.webofscience.com/wos/woscc/summary/59b01abe-f8e9-4548-b879-9e85ab44b3bd-6c0176c4/relevance/1)](https://www.webofscience.com/wos/woscc/summary/51a56905-2f24-42c9-808f-a28d68983976-639b5fdf/relevance/1) |
|  | Assistive devices/technology |  |
| 15 | **((TS=(“Self-help device*” OR “self help device*” OR “sensory aid” OR “orthopedic equipment*” OR wheelchair* OR “wheel chair*” OR crutch* OR walker* OR orthotic* OR prosthetic* OR “walking aid” OR cane* OR “hearing aid” ((assistive OR mobilit*) NEAR/3 (device* OR technolog* OR product* OR equipment* OR tool*)))) OR TI=(“Self-help device*” OR “self help device*” OR “sensory aid” OR “orthopedic equipment*” OR wheelchair* OR “wheel chair*” OR crutch* OR walker* OR orthotic* OR prosthetic* OR “walking aid” OR cane* OR “hearing aid” ((assistive OR mobilit*) NEAR/3 (device* OR technolog* OR product* OR equipment* OR tool*)))) OR AB=(“Self-help device*” OR “self help device*” OR “sensory aid” OR “orthopedic equipment*” OR wheelchair* OR “wheel chair*” OR crutch* OR walker* OR orthotic* OR prosthetic* OR “walking aid” OR cane* OR “hearing aid” ((assistive OR mobilit*) NEAR/3 (device* OR technolog* OR product* OR equipment* OR tool*)))** | [145,562](https://www.webofscience.com/wos/woscc/summary/88889ebe-e6b5-4ad0-b6c6-efb1add54589-6c017f08/relevance/1) |
|  | Rehabilitation |  |
| 16 | **((TS=(Rehabilitation* OR ((physical* OR mental* OR cognitive* OR occupation* OR speech* OR voice* OR vocation*) NEAR/3 (rehabilitat* OR therap*)))) OR TI=(Rehabilitation* OR ((physical* OR mental* OR cognitive* OR occupation* OR speech* OR voice* OR vocation*) NEAR/3 (rehabilitat* OR therap*)))) OR AB=(Rehabilitation* OR ((physical* OR mental* OR cognitive* OR occupation* OR speech* OR voice* OR vocation*) NEAR/3 (rehabilitat* OR therap*)))** | [355,507](https://www.webofscience.com/wos/woscc/summary/1cbd881f-2284-4990-be39-b3ab8d5fe5d6-6c01c036/relevance/1) |
|  | DISABILITIES |  |
| 17 | **#12 OR #11 OR #10 OR #9 OR #8 OR #7 OR #6 OR #5 OR #4 OR #3 OR #2** | [3,244,842](https://www.webofscience.com/wos/woscc/summary/918cb747-1267-49ea-84b0-f1560fb23a92-6c01cf12/relevance/1) |
|  | Low-income countries (LICs) & LMICs |  |
| 18 | **((TS=(“Global south” OR "lower- and middle-income countr*" OR “low- and middle income countr*” LMIC* OR "low-income countr*" OR "low income countr*" OR LIC OR LICs OR (developing OR “low income” OR underdeveloped OR poor OR “low resource*” OR “less developed” OR “under-developed”) NEAR/3 (countr* OR nation* OR world* OR setting* OR population*))) OR TI=(“Global south” OR "lower- and middle-income countr*" OR “low- and middle income countr*” LMIC* OR "low-income countr*" OR "low income countr*" OR LIC OR LICs OR (developing OR “low income” OR underdeveloped OR poor OR “low resource*” OR “less developed” OR “under-developed”) NEAR/3 (countr* OR nation* OR world* OR setting* OR population*))) OR AB=(“Global south” OR "lower- and middle-income countr*" OR “low- and middle income countr*” LMIC* OR "low-income countr*" OR "low income countr*" OR LIC OR LICs OR (developing OR “low income” OR underdeveloped OR poor OR “low resource*” OR “less developed” OR “under-developed”) NEAR/3 (countr* OR nation* OR world* OR setting* OR population*))** | [336,793](https://www.webofscience.com/wos/woscc/summary/2d23ff08-a2db-40f7-bf0b-6bbb3691988a-6c01dd9a/relevance/1) |
|  | LICs |  |
| 19 | **(TI=("Democratic People's Republic of Korea" OR Afghanistan OR Syria OR Yemen OR “Burkina Faso” OR Burundi OR “Central African Republic” OR Chad OR "Democratic Republic of The Congo" OR Congo OR Eritrea OR Ethiopia OR Gambia OR Guinea OR “Guinea-Bisau” OR Liberia OR Madagascar OR Malawi OR Mali OR Mozambique OR Niger OR Rwanda OR Sierra Leone OR Somalia OR “South Sudan” OR Sudan OR Togo OR Uganda)) OR AB=("Democratic People's Republic of Korea" OR Afghanistan OR Syria OR Yemen OR “Burkina Faso” OR Burundi OR “Central African Republic” OR Chad OR "Democratic Republic of The Congo" OR Congo OR Eritrea OR Ethiopia OR Gambia OR Guinea OR “Guinea-Bisau” OR Liberia OR Madagascar OR Malawi OR Mali OR Mozambique OR Niger OR Rwanda OR Sierra Leone OR Somalia OR “South Sudan” OR Sudan OR Togo OR Uganda)** | [335,374](https://www.webofscience.com/wos/woscc/summary/7071d7f1-eba0-4b95-8213-7b633037032a-6c01f86b/relevance/1) |
|  | LMICs |  |
| 20 | **(TI=(“Central Africa” OR “Eastern Africa” OR “Southern Africa” OR “Western Africa” OR Cambodia OR Indonesia OR Kiribati OR Laos OR Lao OR Micronesia OR Mongolia OR Myanmar OR Burma OR “Papua New Guinea” OR Philippines OR Samoa OR “Solomon Islands” OR “Timor Leste” OR “Timor-Leste” OR Vanuatu OR Vietnam OR “Viet Nam” OR “American Samoa” OR China OR Fiji OR Malaysia OR “Marshall Islands” OR Thailand OR Tonga OR Tuvalu OR Kyrgyzstan OR Tajikistan OR Ukraine OR Uzbekistan OR Albania OR Armenia OR Azerbaijan OR Belarus OR “Bosnia-Herzegovina” OR Bulgaria OR Georgia OR Kazakhstan OR Kosovo OR Moldova OR Montenegro OR Macedonia OR Romania OR Russia OR Serbia OR Turkey OR Turkmenistan OR Belize OR Bolivia OR El Salvador OR Haiti OR Honduras Or Nicaragua OR Argentina OR Brazil OR Colombia OR Costa Rica OR Cuba OR Dominica OR Dominican Republic OR Ecuador OR Grenada OR Guatemala OR Guyana OR Jamaica OR Mexico OR Panama OR Paraguay OR Peru OR “Saint Lucia” OR “St Lucia” OR Suriname OR Venezuela OR Algeria OR Djibouti OR Egypt OR Iran OR Morocco OR Tunisia OR Palestine OR Gaza OR Iraq OR Jordan OR Lebanon OR Libya OR Afghanistan OR Bangladesh OR Bhutan OR India OR Nepal OR Pakistan OR “Sri Lanka” OR Maldives OR Angola OR Benin OR Cameroon OR Cape Verde OR Comoros OR Congo OR “Cote d'Ivoire” OR “Ivory Coast” OR Eswatini OR Ghana OR Kenya OR Lesotho OR Mauritania OR Nigeria OR Senegal OR Tanzania OR Zambia OR Zimbabwe OR Botswana OR Equatorial Guinea OR Gabon OR Mauritius OR Namibia OR “South Africa” OR Syria OR Yemen OR “Burkina Faso” OR Burundi OR “Central African Republic” OR Chad OR "Democratic Republic of the Congo" OR Eritrea OR Ethiopia OR Gambia OR Guinea OR “Guinea-Bisau” OR Liberia OR Madagascar OR Malawi OR Mali OR Mozambique OR Niger OR Rwanda OR Sierra Leone OR Somalia OR South Sudan OR Sudan OR Togo OR Uganda)) OR AB=(“Central Africa” OR “Eastern Africa” OR “Southern Africa” OR “Western Africa” OR Cambodia OR Indonesia OR Kiribati OR Laos OR Lao OR Micronesia OR Mongolia OR Myanmar OR Burma OR “Papua New Guinea” OR Philippines OR Samoa OR “Solomon Islands” OR “Timor Leste” OR “Timor-Leste” OR Vanuatu OR Vietnam OR “Viet Nam” OR “American Samoa” OR China OR Fiji OR Malaysia OR “Marshall Islands” OR Thailand OR Tonga OR Tuvalu OR Kyrgyzstan OR Tajikistan OR Ukraine OR Uzbekistan OR Albania OR Armenia OR Azerbaijan OR Belarus OR “Bosnia-Herzegovina” OR Bulgaria OR Georgia OR Kazakhstan OR Kosovo OR Moldova OR Montenegro OR Macedonia OR Romania OR Russia OR Serbia OR Turkey OR Turkmenistan OR Belize OR Bolivia OR El Salvador OR Haiti OR Honduras Or Nicaragua OR Argentina OR Brazil OR Colombia OR Costa Rica OR Cuba OR Dominica OR Dominican Republic OR Ecuador OR Grenada OR Guatemala OR Guyana OR Jamaica OR Mexico OR Panama OR Paraguay OR Peru OR “Saint Lucia” OR “St Lucia” OR Suriname OR Venezuela OR Algeria OR Djibouti OR Egypt OR Iran OR Morocco OR Tunisia OR Palestine OR Gaza OR Iraq OR Jordan OR Lebanon OR Libya OR Afghanistan OR Bangladesh OR Bhutan OR India OR Nepal OR Pakistan OR “Sri Lanka” OR Maldives OR Angola OR Benin OR Cameroon OR Cape Verde OR Comoros OR Congo OR “Cote d'Ivoire” OR “Ivory Coast” OR Eswatini OR Ghana OR Kenya OR Lesotho OR Mauritania OR Nigeria OR Senegal OR Tanzania OR Zambia OR Zimbabwe OR Botswana OR Equatorial Guinea OR Gabon OR Mauritius OR Namibia OR “South Africa” OR Syria OR Yemen OR “Burkina Faso” OR Burundi OR “Central African Republic” OR Chad OR "Democratic Republic of the Congo" OR Eritrea OR Ethiopia OR Gambia OR Guinea OR “Guinea-Bisau” OR Liberia OR Madagascar OR Malawi OR Mali OR Mozambique OR Niger OR Rwanda OR Sierra Leone OR Somalia OR South Sudan OR Sudan OR Togo OR Uganda)** | [3,324,398](https://www.webofscience.com/wos/woscc/summary/db5d6a53-bb7e-42bb-a720-14369841f327-6c01ff62/relevance/1) |
| 21 | **#20 OR #19 OR #18 (LICs OR LMICs)** | [3,541,504](https://www.webofscience.com/wos/woscc/summary/f47b527b-a39d-4b5a-a8a9-2a7f17f294d1-6c020994/relevance/1) |
| 22 | **#1 AND #17 AND #21** | [1,923](https://www.webofscience.com/wos/woscc/summary/d6c4286c-365b-4174-879a-12523f8ff1ac-6c021331/relevance/1) |
| 23 | **#1 AND #17 AND #21** and **2000** or **2001** or **2002** or **2003** or **2023** or **2022** or **2021** or **2020** or **2019** or **2018** or **2017** or **2016** or **2015** or **2014** or **2013** or **2012** or **2011** or **2010** or **2009** or **2008** or **2007** or **2006** or **2005** or **2004** or **2003** or **2002** or **2001** or **2000** (Publication Years) | [1,886](https://www.webofscience.com/wos/woscc/summary/b87d9737-a9af-403f-a9ac-5221f5d9a6bb-6c0220ca/relevance/1) |
| 24 | **#1 AND #17 AND #21** and **2000** or **2001** or **2002** or **2003** or **2023** or **2022** or **2021** or **2020** or **2019** or **2018** or **2017** or **2016** or **2015** or **2014** or **2013** or **2012** or **2011** or **2010** or **2009** or **2008** or **2007** or **2006** or **2005** or **2004** or **2003** or **2002** or **2001** or **2000** (Publication Years) and **Article** (Document Types) | [1,618](https://www.webofscience.com/wos/woscc/summary/ad821ade-201a-4a41-b459-fb990b84aabd-6c02314b/relevance/1) |
| 25 | **#1 AND #17 AND #21** and **2000** or **2001** or **2002** or **2003** or **2023** or **2022** or **2021** or **2020** or **2019** or **2018** or **2017** or **2016** or **2015** or **2014** or **2013** or **2012** or **2011** or **2010** or **2009** or **2008** or **2007** or **2006** or **2005** or **2004** or **2003** or **2002** or **2001** or **2000** (Publication Years) and **Article** (Document Types) and **English** (Languages) | [1,540](https://www.webofscience.com/wos/woscc/summary/15e1d466-13db-482d-8f4b-af42b6983120-6c023332/relevance/1) |

1. **Scopus**

Search date: January 24, 2023

| No | Search terms (SEARCH IN TITLE, ABSTRACT, KEYWORDS) | Results |
| --- | --- | --- |
|  | **Health Insurance** |  |
| 1 | “Health insurance*” OR “disability insurance*” OR “medical aid” OR “medical insurance*” OR “health aid” OR “universal health coverage” OR “health fund*” OR “health financ*” OR insure* OR social health protection* OR “social health insurance*” OR community health fund* OR “community-based insurance*” OR “community based insurance” OR health financing OR (health W/3 insurance*) OR (disabilit* W/3 insurance*) | [24,529 results](https://www-scopus-com.ez.lshtm.ac.uk/search/history/results.uri?origin=searchhistory&shid=1) |
|  | **People with disabilities** |  |
| 2 | disabilit* OR disabled OR impairment* OR impair* OR handicap* | [1,665,133 results](https://www-scopus-com.ez.lshtm.ac.uk/search/history/results.uri?origin=searchhistory&shid=2) |
| 3 | ((person* OR people OR individ* OR patient* OR subject* OR adult* OR elderly OR child* OR boy* OR girl* OR kid* OR m?n OR wom?n OR teenager* OR juvenile OR adolescent* OR body OR bodies) W/5 (disabilit* OR disable* OR disabl* OR handicap* OR “function* limitation*” OR “function* diversit*” OR dependen* OR “special need*” OR “rare disease*” OR incapacit* OR impairment*)) | [514,981 results](https://www-scopus-com.ez.lshtm.ac.uk/search/history/results.uri?origin=searchhistory&shid=3) |
|  | **Physical disabilities** |  |
| 4 | “physical disabilit*” OR “cerebral pals*” OR “spinal dysraphism” OR “muscular dystroph*” OR “rheumatoid arthritis” OR “osteogenesis imperfecta” OR “limb malformation*”OR amputation* OR amputee* OR “arm amputation*” OR “leg amputation*” OR “limb amputation*” OR “above knee amputation*” OR “below knee amputation*” OR “finger amputation*” OR “traumatic amputation*” OR “congenital amputation*” OR “foot amputation*”OR “hand amputation*” OR “knee amputation*” OR “thumb amputation*” OR arthrogryposis OR clubfoot OR poliomyelitis OR paralys* OR paralyz* OR “hereditary motor sensory neuropathy” OR parapleg* OR quadripleg* OR tetrapleg* OR hemipleg* OR “spinal cord injur*” OR “spina bifida” OR “muscular distroph*” OR polio OR “wheelchair user*” OR “wheel chair user*” | [781,481 results](https://www-scopus-com.ez.lshtm.ac.uk/search/history/results.uri?origin=searchhistory&shid=4) |
| 5 | (Physical* W/3 (impair* OR deficienc* OR disable* OR disabilit* OR handicap* OR incapacit* OR defect*)) | [56,067 results](https://www-scopus-com.ez.lshtm.ac.uk/search/history/results.uri?origin=searchhistory&shid=5) |
|  | **Hearing disabilities** |  |
| 6 | (deaf* OR “deaf-blind disorder*” OR ((hearing or acoustic) W/3 (loss* OR impair* OR deficienc* OR disable* OR disabilit* OR handicap* OR defect*))) | [191,058 results](https://www-scopus-com.ez.lshtm.ac.uk/search/history/results.uri?origin=searchhistory&shid=6) |
|  | **Visual disabilities** |  |
| 7 | Blind* OR ((visual* OR vision) W/3 (loss* OR disabilit* OR disorder* OR impairment* OR disable* OR deficienc* OR handicap* OR deprivation* OR defect*)) | [746,600 results](https://www-scopus-com.ez.lshtm.ac.uk/search/history/results.uri?origin=searchhistory&shid=7) |
|  | **Speech disabilities** |  |
| 8 | ((speech* OR communication* OR language) W/3 (disabilit* OR disorder* OR impair* OR deficienc* OR disable* OR handicap*)) | [88,499 results](https://www-scopus-com.ez.lshtm.ac.uk/search/history/results.uri?origin=searchhistory&shid=8) |
|  | **Intellectual disabilities** |  |
| 9 | ((intellectual OR cognition OR cognitive OR learning OR developmental) W/3 (disabilit* OR disable* OR impair* OR disorder* OR incapacit* OR handicap* OR dysfunction* OR defect* OR problem* OR deficit* OR difficult*)) OR autism* OR ADHD OR “attention deficit hyperactivity disorder*” OR “attention-deficit hyperactivity disorder*” OR “down syndrome*” OR dementia OR Alzheimer | [998,872 results](https://www-scopus-com.ez.lshtm.ac.uk/search/history/results.uri?origin=searchhistory&shid=9) |
|  | **Mental disabilities** |  |
| 10 | ((Mental* OR psychological* OR behavior*) W/3 (disabilit* OR disable* OR impair* OR handicap* OR incapacit* OR disorder* OR illness* OR ill* OR dysfunction* OR retard* OR deficienc* OR disease* OR diagnos*)) OR psychosis OR psychoses OR schizoaffective OR schizophreniform OR anxiet* OR depression* OR schizophrenia OR bipolar OR “mental health condition*” | [1,975,406 results](https://www-scopus-com.ez.lshtm.ac.uk/search/history/results.uri?origin=searchhistory&shid=10) |
|  | **Assistive devices/technology** |  |
| 11 | “Self-help device*” OR “self help device*” OR “sensory aid” OR “orthopedic equipment*” OR wheelchair* OR “wheel chair*” OR crutch* OR walker* OR orthotic* OR prosthetic* OR “walking aid” OR cane* OR “hearing aid” ((assistive OR mobilit*) W/3 (device* OR technolog* OR product* OR equipment* OR tool*)) | [6,035 results](https://www-scopus-com.ez.lshtm.ac.uk/search/history/results.uri?origin=searchhistory&shid=11) |
|  | Rehabilitation |  |
| 12 | Rehabilitation* OR ((physical* OR mental* OR cognitive* OR occupation* OR speech* OR voice* OR vocation*) W/3 (rehabilitat* OR therap*)) | [607,820 results](https://www-scopus-com.ez.lshtm.ac.uk/search/history/results.uri?origin=searchhistory&shid=12) |
|  | DISABILITIES |  |
| 13 | **#2 OR #3 OR #4 OR #5 OR #6 OR #7 OR #8 OR #9 OR #10 OR #11 OR #12** | [5,706,079 results](https://www-scopus-com.ez.lshtm.ac.uk/search/history/results.uri?origin=searchhistory&shid=13) |
|  | Low-income countries (LICs) & LMICs |  |
| 14 | “Global south” OR "lower- and middle-income countr*" OR “low- and middle income countr*” LMIC* OR "low-income countr*" OR "low income countr*" OR LIC OR LICs OR (developing OR “low income” OR underdeveloped OR poor OR “low resource*” OR “less developed” OR “under-developed”) W/3 (countr* OR nation* OR world* OR setting* OR population*) | [15,675 results](https://www-scopus-com.ez.lshtm.ac.uk/search/history/results.uri?origin=searchhistory&shid=14) |
|  | LICs |  |
| 15 | "Democratic People's Republic of Korea" OR Afghanistan OR Syria OR Yemen OR “Burkina Faso” OR Burundi OR “Central African Republic” OR Chad OR "Democratic Republic of The Congo" OR Congo OR Eritrea OR Ethiopia OR Gambia OR Guinea OR “Guinea-Bisau” OR Liberia OR Madagascar OR Malawi OR Mali OR Mozambique OR Niger OR Rwanda OR Sierra Leone OR Somalia OR “South Sudan” OR Sudan OR Togo OR Uganda | [18,301 results](https://www-scopus-com.ez.lshtm.ac.uk/search/history/results.uri?origin=searchhistory&shid=15) |
|  | LMICs |  |
| 16 | “Central Africa” OR “Eastern Africa” OR “Southern Africa” OR “Western Africa” OR Cambodia OR Indonesia OR Kiribati OR Laos OR Lao OR Micronesia OR Mongolia OR Myanmar OR Burma OR “Papua New Guinea” OR Philippines OR Samoa OR “Solomon Islands” OR “Timor Leste” OR “Timor-Leste” OR Vanuatu OR Vietnam OR “Viet Nam” OR “American Samoa” OR China OR Fiji OR Malaysia OR “Marshall Islands” OR Thailand OR Tonga OR Tuvalu OR Kyrgyzstan OR Tajikistan OR Ukraine OR Uzbekistan OR Albania OR Armenia OR Azerbaijan OR Belarus OR “Bosnia-Herzegovina” OR Bulgaria OR Georgia OR Kazakhstan OR Kosovo OR Moldova OR Montenegro OR Macedonia OR Romania OR Russia OR Serbia OR Turkey OR Turkmenistan OR Belize OR Bolivia OR El Salvador OR Haiti OR Honduras Or Nicaragua OR Argentina OR Brazil OR Colombia OR Costa Rica OR Cuba OR Dominica OR Dominican Republic OR Ecuador OR Grenada OR Guatemala OR Guyana OR Jamaica OR Mexico OR Panama OR Paraguay OR Peru OR “Saint Lucia” OR “St Lucia” OR Suriname OR Venezuela OR Algeria OR Djibouti OR Egypt OR Iran OR Morocco OR Tunisia OR Palestine OR Gaza OR Iraq OR Jordan OR Lebanon OR Libya | [2,537 results](https://www-scopus-com.ez.lshtm.ac.uk/search/history/results.uri?origin=searchhistory&shid=16) |
| 17 | Afghanistan OR Bangladesh OR Bhutan OR India OR Nepal OR Pakistan OR “Sri Lanka” OR Maldives OR Angola OR Benin OR Cameroon OR Cape Verde OR Comoros OR Congo OR “Cote d'Ivoire” OR “Ivory Coast” OR Eswatini OR Ghana OR Kenya OR Lesotho OR Mauritania OR Nigeria OR Senegal OR Tanzania OR Zambia OR Zimbabwe OR Botswana OR Equatorial Guinea OR Gabon OR Mauritius OR Namibia OR “South Africa” OR Syria OR Yemen OR “Burkina Faso” OR Burundi OR “Central African Republic” OR Chad OR "Democratic Republic of the Congo" OR Eritrea OR Ethiopia OR Gambia OR Guinea OR “Guinea-Bisau” OR Liberia OR Madagascar OR Malawi OR Mali OR Mozambique OR Niger OR Rwanda OR Sierra Leone OR Somalia OR South Sudan OR Sudan OR Togo OR Uganda | [1,074 results](https://www-scopus-com.ez.lshtm.ac.uk/search/history/results.uri?origin=searchhistory&shid=17) |
| 18 | **#17 OR #16 OR #15 OR #14 (LICs OR LMICs)** | [36,284 results](https://www-scopus-com.ez.lshtm.ac.uk/search/history/results.uri?origin=searchhistory&shid=18) |
| 19 | **#1 AND #13 AND #18** | [28 results](https://www-scopus-com.ez.lshtm.ac.uk/search/history/results.uri?origin=searchhistory&shid=19) |
|  |  |  |

1. **Cochrane**

Search date: January 24, 2023

| ID | Search | Results |
| --- | --- | --- |
| #1 | MeSH descriptor: [Insurance, Health] explode all trees | 1263 |
| #2 | (health insurance):ti,ab,kw | 4382 |
| #3 | (medical aid):ti,ab,kw | 2133 |
| #4 | (health fund*):ti,ab,kw | 13323 |
| #5 | (health fund* OR health financ*):ti,ab,kw | 17495 |
| #6 | (insuranc*):ti,ab,kw | 5555 |
| #7 | (social health protection*):ti,ab,kw | 531 |
| #8 | (social health protection* OR social health insurance* OR community health fund* OR community health insurance *OR community based insurance*):ti,ab,kw | 3483 |
| #9 | (disability near/3 insuranc*):ti,ab,kw | 63 |
| #10 | (health near/3 insuranc*):ti,ab,kw | 3126 |
| #11 | #1 OR #2 OR #3 #4 OR #5 OR #6 OR #7 OR #8 OR #9 OR #10 | 23574 |
| #12 | MeSH descriptor: [Disabled Persons] explode all trees | 1298 |
| #13 | MeSH descriptor: [Deaf-Blind Disorders] explode all trees | 11 |
| #14 | MeSH descriptor: [Persons With Hearing Impairments] explode all trees | 55 |
| #15 | MeSH descriptor: [Intellectual Disability] explode all trees | 1601 |
| #16 | MeSH descriptor: [Mental Disorders] explode all trees | 83362 |
| #17 | MeSH descriptor: [Developmental Disabilities] explode all trees | 694 |
| #18 | MeSH descriptor: [Speech Disorders] explode all trees | 977 |
| #19 | MeSH descriptor: [Language Disorders] explode all trees | 1588 |
| #20 | MeSH descriptor: [Learning Disabilities] explode all trees | 624 |
| #21 | MeSH descriptor: [Disabled Children] explode all trees | 128 |
| #22 | MeSH descriptor: [Cerebral Palsy] explode all trees | 1730 |
| #23 | MeSH descriptor: [Amputation] explode all trees | 471 |
| #24 | MeSH descriptor: [Spinal Cord Injuries] explode all trees | 1972 |
| #25 | MeSH descriptor: [Poliomyelitis] explode all trees | 364 |
| #26 | MeSH descriptor: [Paralysis] explode all trees | 2338 |
| #27 | MeSH descriptor: [Osteogenesis Imperfecta] explode all trees | 64 |
| #28 | MeSH descriptor: [Limb Deformities, Congenital] explode all trees | 243 |
| #29 | MeSH descriptor: [Foot Deformities] explode all trees | 506 |
| #30 | MeSH descriptor: [Hearing Loss] explode all trees | 1374 |
| #31 | MeSH descriptor: [Vision Disorders] explode all trees | 1645 |
| #32 | MeSH descriptor: [Communication Disorders] explode all trees | 1983 |
| #33 | MeSH descriptor: [Cognition Disorders] explode all trees | 6209 |
| #34 | MeSH descriptor: [Dementia] explode all trees | 6885 |
| #35 | MeSH descriptor: [Alzheimer Disease] explode all trees | 3874 |
| #36 | MeSH descriptor: [Autism Spectrum Disorder] explode all trees | 1927 |
| #37 | MeSH descriptor: [Depression] explode all trees | 14391 |
| #38 | MeSH descriptor: [Self-Help Devices] explode all trees | 449 |
| #39 | MeSH descriptor: [Spinal Dysraphism] explode all trees | 80 |
| #40 | MeSH descriptor: [Sensory Aids] explode all trees | 500 |
| #41 | MeSH descriptor: [Orthopedic Equipment] explode all trees | 4937 |
| #42 | MeSH descriptor: [Rehabilitation] explode all trees | 41529 |
| #43 | ((person* OR people OR individ* OR patient* OR subject* OR adult* OR elderly OR child* OR boy* OR girl* OR kid* OR m?n OR wom?n OR teenager* OR juvenile OR adolescent* OR body OR bodies) NEAR/5 (disabilit* OR disable* OR disabl* OR handicap* OR “function* limitation*” OR “function* diversit*” OR dependen* OR “special need*” OR “rare disease*” OR incapacit* OR impairment*)):ti,ab,kw | 42634 |
| #44 | (“physical disabilit*” OR “cerebral pals*” OR “spinal dysraphism” OR “muscular dystroph*” OR “rheumatoid arthritis” OR “osteogenesis imperfecta” OR “limb malformation*”OR amputation* OR amputee* OR “arm amputation*” OR “leg amputation*” OR “limb amputation*” OR “above knee amputation*” OR “below knee amputation*” OR “finger amputation*” OR “traumatic amputation*” OR “congenital amputation*” OR “foot amputation*”OR “hand amputation*” OR “knee amputation*” OR “thumb amputation*” OR arthrogryposis OR clubfoot OR poliomyelitis OR paralys* OR “hereditary motor sensory neuropathy” OR parapleg* OR quadripleg* OR tetrapleg* OR hemipleg* OR paralyz* OR “spinal cord injur*” OR “spina bifida” OR “muscular distroph*” OR polio OR “wheelchair user*” OR “wheel chair user*”):ti,ab,kw | 0 |
| #45 | (Physical* NEAR/3 (impair* OR deficienc* OR disable* OR disabilit* OR handicap* OR incapacit* OR defect*)):ti,ab,kw | 4088 |
| #46 | (deaf* OR “deaf-blind disorder*” OR ((hearing or acoustic) NEAR/3 (loss* OR impair* OR deficienc* OR disable* OR disabilit* OR handicap* OR defect*)) NOT (blinding OR "double blind*" OR "triple blind*")):ti,ab,kw | 5224 |
| #47 | (Blind* OR ((visual* OR vision) NEAR/3 (loss* OR disabilit* OR disorder* OR impairment* OR disable* OR deficienc* OR handicap* OR deprivation* OR defect*)) NOT (blinding OR "double blind*" OR "triple blind*")):ti,ab,kw | 433514 |
| #48 | ((speech* OR communication* OR language) NEAR/3 (disabilit* OR disorder* OR impair* OR deficienc* OR disable* OR handicap*)):ti,ab,kw | 2597 |
| #49 | ((intellectual OR cognition OR cognitive OR learning OR developmental) NEAR/3 (disabilit* OR disable* OR impair* OR disorder* OR incapacit* OR handicap* OR dysfunction* OR defect* OR problem* OR deficit* OR difficult*)):ti,ab,kw | 30729 |
| #50 | ((Mental* OR psychological* OR behavio$r*) NEAR/3 (disabilit* OR disable* OR impair* OR handicap* OR incapacit* OR disorder* OR illness* OR ill* OR dysfunction* OR retard* OR deficienc* OR disease* OR diagnos*)):ti,ab,kw | 29341 |
| #51 | (autism* OR ADHD OR “attention deficit hyperactivity disorder*” OR “attention-deficit hyperactivity disorder*” OR “down syndrome*” OR dementia OR Alzheimer):ti,ab,kw | 33986 |
| #52 | ((assistive OR mobilit*) NEAR/3 (device* OR technolog* OR product* OR equipment* OR tool*)):ti,ab,kw | 1155 |
| #53 | ((physical* OR mental* OR cognitive* OR occupation* OR speech* OR voice* OR vocation*) NEAR/3 (rehabilitat* OR therap*)):ti,ab,kw | 52939 |
| #54 | #12 OR #13 OR #14 OR #15 OR #16 OR #16 OR #17 OR #18 OR #19 OR #20 OR #21 OR #22 OR #23 OR #24 OR #25 OR #26 OR #27 OR #28 OR #29 OR #30 OR #31 OR #32 OR #33 OR #34 OR #35 OR #36 OR #37 OR #38 OR #39 OR #40 OR #41 OR #42 OR #43 OR #44 OR #45 OR #46 OR #47 OR #48 OR #49 OR #50 OR #51 OR #52 OR #53 | 1979014 |
| #55 | ("Democratic People's Republic of Korea" OR Afghanistan OR Syria OR Yemen OR Burkina Faso OR Burundi OR Central African Republic OR Chad OR "Democratic Republic of The Congo" OR Congo OR Eritrea OR Ethiopia OR Gambia OR Guinea OR Guinea-Bisau OR Liberia OR Madagascar OR Malawi OR Mali OR Mozambique OR Niger OR Rwanda OR Sierra Leone OR Somalia OR South Sudan OR Sudan OR Togo OR Uganda):ti,ab,kw | 9905 |
| #56 | (Global south OR "lower- and middle-income countries" OR "lower- and middle-income country" OR LMIC* OR "low-income country" OR "low income countries" OR LIC OR LICs):ti,ab,kw | 2461 |
| #57 | (developing countr* OR low income countr* OR uderdeveloped countr* OR poor countr* OR less developed countr* OR underdeveloped nation* OR poor nation* OR underdeveloped world* OR low resource countr* OR low resource nation*):ti,ab,kw | 12637 |
| #58 | (Central Africa OR Eastern Africa OR Southern Africa OR Western Africa OR Cambodia OR Indonesia OR Kiribati OR Laos OR Lao OR Micronesia OR Mongolia OR Myanmar OR Burma OR Papua New Guinea OR Philippines OR Samoa OR Solomon Islands OR Timor Leste OR Timor-Leste OR Vanuatu OR Vietnam OR Viet Nam OR American Samoa OR China OR Fiji OR Malaysia OR Marshall Islands OR Thailand OR Tonga OR Tuvalu OR Kyrgyzstan OR Tajikistan OR Ukraine OR Uzbekistan OR Albania OR Armenia OR Azerbaijan OR Belarus OR Bosnia-Herzegovina OR Bulgaria OR Georgia OR Kazakhstan OR Kosovo OR Moldova OR Montenegro OR Macedonia OR Romania OR Russia OR Serbia OR Turkey OR Turkmenistan OR Belize OR Bolivia OR El Salvador OR Haiti OR Honduras Or Nicaragua OR Argentina OR Brazil OR Colombia OR Costa Rica OR Cuba OR Dominica OR Dominican Republic OR Ecuador OR Grenada OR Guatemala OR Guyana OR Jamaica OR Mexico OR Panama OR Paraguay OR Peru OR Saint Lucia OR St Lucia OR Suriname OR Venezuela OR Algeria OR Djibouti OR Egypt OR Iran OR Morocco OR Tunisia OR Palestine OR Gaza OR Iraq OR Jordan OR Lebanon OR Libya OR Afghanistan OR Bangladesh OR Bhutan OR India OR Nepal OR Pakistan OR Sri Lanka OR Maldives OR Angola OR Benin OR Cameroon OR Cape Verde OR Comoros OR Congo OR (Cote d'Ivoire) OR Ivory Coast OR Eswatini OR Ghana OR Kenya OR Lesotho OR Mauritania OR Nigeria OR Senegal OR Tanzania OR Zambia OR Zimbabwe OR Botswana OR Equatorial Guinea OR Gabon OR Mauritius OR Namibia OR South Africa OR Syria OR Yemen OR Burkina Faso OR Burundi OR Central African Republic OR Chad OR "Democratic Republic of the Congo" OR Eritrea OR Ethiopia OR Gambia OR Guinea OR Guinea-Bisau OR Liberia OR Madagascar OR Malawi OR Mali OR Mozambique OR Niger OR Rwanda OR Sierra Leone OR Somalia OR South Sudan OR Sudan OR Togo OR Uganda):ti,ab,kw | 94325 |
| #59 | #55 OR #56 OR #57 OR #58 | 101203 |
| #60 | #11 AND #54 AND #59 with Publication Year from 2000 to 2023, with Cochrane Library publication date Between Jan 2000 and Dec 2023, in Trials (Word variations have been searched) | 3651 |

1. **Ovid PsyInfo**

Search date: January 24, 2023

| No | Search strategies | Results |
| --- | --- | --- |
| 1 | exp Health Insurance/ | 12496 |
| 2 | (Health insurance* or medical aid or medical insurance* or universal health coverage or health fund* or health financ* or social health protection* or social health insurance* or community health fund* or community-based insurance* or health financing or (health adj5 insuranc*) or disability insuranc*).ti,ab. | 11541 |
| 3 | 1 or 2 | 20411 |
| 4 | exp disabilities/ or exp multiple disabilities/ | 57965 |
| 5 | ((disabilit* or disable* or handicap* or function* limitation* or function* diversit* or dependen* or special need* or rare disease* or incapacity* or impairment*) adj5 (person* or people or individ* or patient* or subject* or adult* or elderly or child* or boy* or girl* or kid* or m#n or wom#n or teenager* or juvenile or adolescent*)).ti,ab. | 148814 |
| 6 | exp physical disorders/ or exp cerebral palsy/ or exp spina bifida/ or exp nervous system disorders/ or exp spinal cord injuries/ or exp muscular dystrophy/ or exp arthritis/ or exp physical disfigurement/ or exp amputation/ or exp poliomyelitis/ or exp paralysis/ or exp paraplegia/ or exp quadriplegia/ | 662577 |
| 7 | (Physical* adj3 (impair* or deficienc* or disable* or disabilit* or handicap* or incapacit*)).ti,ab. | 12636 |
| 8 | (Cerebral pals* or spina bifida or muscular distroph* or arthriti* or osteogenesis imperfecta or polio or poliomyelitis or paralyz* or paralys* or tetraplegi* or quadriplegi* or paraplegi* or hemiplegi* or wheelchair user* or wheel chair user* or amput*).ti,ab. | 27684 |
| 9 | exp hearing disorders/ or exp deaf/ or exp deaf blind/ | 19315 |
| 10 | ((deaf* or deaf-blind disorder* or ((hearing or acoustic) adj3 (loss* or impair* or deficienc* or disable* or disabilit* or handicap*))) not (blinding or double blind* or triple blind*)).ti,ab. | 30238 |
| 11 | exp vision disorders/ or exp visual impairment/ or exp visually handicapped/ or exp blind/ | 18466 |
| 12 | ((Blind* or ((visual* or vision) adj3 (loss* disabilit* or disorder* or impairment* or disable* or deficienc* or handicap*))) not (blinding or double blind* or triple blind*)).ti,ab. | 38708 |
| 13 | exp speech disorders/ or exp communication disorders/ or language disorders/ | 61413 |
| 14 | ((speech* or communication* or language*) adj3 (disabilit* or disorder* or impair* or deficienc* or disable* or handicap*)).ti,ab. | 22228 |
| 15 | exp intellectual development disorder/ or exp developmental disabilities/ or exp learning disabilities/ or exp cognitive impairment/ or exp autism spectrum disorders/ | 176258 |
| 16 | (((intellectual or cognition or cognitive or learning or developmental) adj3 (disabilit* or disable* or impair* or disorder* or incapacit* or handicap*)) or autism* or ADHD or attention deficit hyperactivity disorder* or attention-deficit hyperactivity disorder* or down syndrome or dementia or Alzheimer).ti,ab. | 266558 |
| 17 | exp mental disorders/ or exp serious mental illness/ or exp psychiatric patients/ or exp chronic mental illness/ | 975264 |
| 18 | (((Mental* or psychological* or behavio?r*) adj3 (disabilit* or disable* or impair* or handicap* or incapacit* or disorder* or illness* or ill* or dysfunction* or retard* or deficienc* or disease* or diagnos*)) or psychosis or psychoses or schizoaffective or schizophreniform or schizophrenia or bipolar or mental health condition*).ti,ab. | 362996 |
| 19 | exp assistive technology/ or exp mobility aids/ or exp hearing aids/ or exp optical aids/ | 12106 |
| 20 | (Magnifier* or medical device* or ((assistive or mobilit*) adj3 (device* or technolog* or product* or equipment* or tool*)) or hearing aid* or wheelchair* or wheel chair* or orthotic* or prosthetic*).ti,ab. | 9946 |
| 21 | exp rehabilitation/ or exp physical therapy/ or exp speech therapy/ or exp occupational therapy/ or exp cognitive therapy/ | 72173 |
| 22 | ((physical* or mental* or cognitive* or occupation* or speech* or voice* or vocation*) adj3 (rehabilitat* or therap*)).ti,ab. | 67286 |
| 23 | 4 or 5 or 6 or 7 or 8 or 9 or 10 or 11 or 12 or 13 or 14 or 15 or 16 or 17 or 18 or 19 or 20 or 21 or 22 | 1702257 |
| 24 | developing country/ or low income country/ or middle income country/ | 0 |
| 25 | ((developing or less* developed or under developed or underdeveloped or middle income or low* income) adj (economy or economies)).ti,ab. | 463 |
| 26 | ((developing or less* developed or under developed or underdeveloped or middle income or low* income or underserved or under served or deprived or poor*) adj (countr* or nation? or population? or world)).ti,ab. | 21665 |
| 27 | (low* adj (gdp or gnp or gross domestic or gross national)).ti,ab. | 52 |
| 28 | (low adj3 middle adj3 countr*).ti,ab. | 4725 |
| 29 | (lmic or lmics or third world or lami countr*).ti,ab. | 2493 |
| 30 | transitional countr*.ti,ab. | 66 |
| 31 | global south.ti,ab. | 875 |
| 32 | "Africa south of the Sahara"/ | 0 |
| 33 | ("Africa South of the Sahara" or sub-Saharan Africa or subSaharan Africa).ti,ab. | 4090 |
| 34 | Central Africa.ti,ab. | 217 |
| 35 | Eastern Africa.ti,ab. | 114 |
| 36 | Southern Africa.ti,ab. | 679 |
| 37 | Western Africa.ti,ab. | 59 |
| 38 | North Korea/ | 0 |
| 39 | (North Korea or (Democratic People* Republic adj2 Korea)).ti,ab. | 176 |
| 40 | Haiti/ | 0 |
| 41 | (Haiti or Hayti).ti,ab. | 922 |
| 42 | Afghanistan/ | 0 |
| 43 | Afghanistan.ti,ab. | 3249 |
| 44 | Nepal/ | 0 |
| 45 | Nepal.ti,ab. | 1588 |
| 46 | Syrian Arab Republic/ | 0 |
| 47 | (Syria or Syrian Arab Republic).ti,ab. | 773 |
| 48 | Yemen/ | 0 |
| 49 | Yemen.ti,ab. | 225 |
| 50 | Tajikistan/ | 0 |
| 51 | Tajikistan.ti,ab. | 135 |
| 52 | Benin/ | 0 |
| 53 | (Benin or Dahomey).ti,ab. | 311 |
| 54 | Burkina Faso/ | 0 |
| 55 | (Burkina Faso or Burkina Fasso or Upper Volta).ti,ab. | 378 |
| 56 | Burundi/ | 0 |
| 57 | (Burundi or Ruanda-Urundi).ti,ab. | 171 |
| 58 | Central African Republic/ | 0 |
| 59 | (Central African Republic or Ubangi-Shari).ti,ab. | 85 |
| 60 | Chad/ | 0 |
| 61 | Chad.ti,ab. | 167 |
| 62 | Democratic Republic Congo/ | 0 |
| 63 | (((Democratic Republic or DR) adj2 Congo) or Congo-Kinshasa or Belgian Congo or Zaire or Congo Free State).ti,ab. | 627 |
| 64 | Eritrea/ | 0 |
| 65 | Eritrea.ti,ab. | 124 |
| 66 | Ethiopia/ | 0 |
| 67 | (Ethiopia or Abyssinia).ti,ab. | 2250 |
| 68 | Gambia/ | 0 |
| 69 | Gambia.ti,ab. | 176 |
| 70 | Guinea/ | 1966 |
| 71 | (Guinea not (New Guinea or Guinea Pig* or Guinea Fowl or Guinea-Bissau or Portuguese Guinea or Equatorial Guinea)).ti,ab. | 286 |
| 72 | Guinea-Bissau/ | 0 |
| 73 | (Guinea-Bissau or Portuguese Guinea).ti,ab. | 71 |
| 74 | Liberia/ | 0 |
| 75 | Liberia.ti,ab. | 424 |
| 76 | Madagascar/ | 0 |
| 77 | (Madagascar or Malagasy Republic).ti,ab. | 565 |
| 78 | Malawi/ | 0 |
| 79 | (Malawi or Nyasaland).ti,ab. | 1239 |
| 80 | Mali/ | 0 |
| 81 | Mali.ti,ab. | 318 |
| 82 | Mozambique/ | 0 |
| 83 | (Mozambique or Mocambique or Portuguese East Africa).ti,ab. | 564 |
| 84 | Niger/ | 0 |
| 85 | (Niger not (Aspergillus or Peptococcus or Schizothorax or Cruciferae or Gobius or Lasius or Agelastes or Melanosuchus or radish or Parastromateus or Orius or Apergillus or Parastromateus or Stomoxys)).ti,ab. | 370 |
| 86 | Rwanda/ | 0 |
| 87 | (Rwanda or Ruanda).ti,ab. | 1027 |
| 88 | Sierra Leone/ | 0 |
| 89 | (Sierra Leone or Salone).ti,ab. | 518 |
| 90 | Somalia/ | 0 |
| 91 | (Somalia or Somaliland).ti,ab. | 424 |
| 92 | south sudan/ | 0 |
| 93 | South Sudan.ti,ab. | 128 |
| 94 | Tanzania/ | 0 |
| 95 | (Tanzania or Tanganyika or Zanzibar).ti,ab. | 2392 |
| 96 | Togo/ | 0 |
| 97 | (Togo or Togolese Republic or Togoland).ti,ab. | 168 |
| 98 | Uganda/ | 0 |
| 99 | Uganda.ti,ab. | 3348 |
| 100 | Cambodia/ | 0 |
| 101 | Cambodia.ti,ab. | 832 |
| 102 | exp Indonesia/ | 0 |
| 103 | (Indonesia or Dutch East Indies).ti,ab. | 3056 |
| 104 | kiribati/ | 0 |
| 105 | (Kiribati or Gilbert Islands or Phoenix Islands or Line Islands).ti,ab. | 30 |
| 106 | Laos/ | 0 |
| 107 | (Laos or (Lao adj1 Democratic Republic)).ti,ab. | 314 |
| 108 | exp "Federated States of Micronesia"/ | 0 |
| 109 | Micronesia.ti,ab. | 150 |
| 110 | Mongolia/ | 0 |
| 111 | Mongolia.ti,ab. | 313 |
| 112 | Myanmar/ | 0 |
| 113 | (Myanmar or Burma).ti,ab. | 594 |
| 114 | Papua New Guinea/ | 0 |
| 115 | (Papua New Guinea or German New Guinea or British New Guinea or Territory of Papua).ti,ab. | 662 |
| 116 | Philippines/ | 0 |
| 117 | (Philippines or Philippine Islands).ti,ab. | 2340 |
| 118 | solomon islands/ | 0 |
| 119 | Solomon Islands.ti,ab. | 132 |
| 120 | Timor-Leste/ | 0 |
| 121 | (Timor-Leste or East Timor or Portuguese Timor).ti,ab. | 159 |
| 122 | Vanuatu/ | 0 |
| 123 | (Vanuatu or New Hebrides).ti,ab. | 139 |
| 124 | Viet Nam/ | 0 |
| 125 | (Viet Nam or Vietnam or French Indochina).ti,ab. | 6026 |
| 126 | Kyrgyzstan/ | 0 |
| 127 | (Kyrgyzstan or Kyrgyz Republic or Kirghizia or Kirghiz).ti,ab. | 234 |
| 128 | Moldova/ | 0 |
| 129 | Moldova.ti,ab. | 163 |
| 130 | exp Ukraine/ | 0 |
| 131 | Ukraine.ti,ab. | 1020 |
| 132 | exp Uzbekistan/ | 0 |
| 133 | Uzbekistan.ti,ab. | 124 |
| 134 | Bolivia/ | 0 |
| 135 | Bolivia.ti,ab. | 623 |
| 136 | El Salvador/ | 0 |
| 137 | El Salvador.ti,ab. | 569 |
| 138 | Honduras/ | 0 |
| 139 | Honduras.ti,ab. | 406 |
| 140 | Nicaragua/ | 0 |
| 141 | Nicaragua.ti,ab. | 528 |
| 142 | Djibouti/ | 0 |
| 143 | (Djibouti or French Somaliland).ti,ab. | 27 |
| 144 | Egypt/ | 0 |
| 145 | Egypt.ti,ab. | 2167 |
| 146 | Morocco/ | 0 |
| 147 | Morocco.ti,ab. | 793 |
| 148 | Tunisia/ | 0 |
| 149 | Tunisia.mp. | 705 |
| 150 | palestine/ | 0 |
| 151 | (Gaza or West Bank or Palestine).ti,ab. | 1656 |
| 152 | Bangladesh/ | 0 |
| 153 | Bangladesh.ti,ab. | 2284 |
| 154 | Bhutan/ | 0 |
| 155 | Bhutan.ti,ab. | 214 |
| 156 | exp India/ | 7307 |
| 157 | India.ti,ab. | 18743 |
| 158 | exp Pakistan/ | 0 |
| 159 | Pakistan.ti,ab. | 3418 |
| 160 | Angola/ | 0 |
| 161 | Angola.ti,ab. | 196 |
| 162 | Cameroon/ | 0 |
| 163 | (Cameroon or Kamerun or Cameroun).ti,ab. | 704 |
| 164 | Cape Verde/ | 0 |
| 165 | (Cape Verde or Cabo Verde).ti,ab. | 72 |
| 166 | Comoros/ | 0 |
| 167 | (Comoros or Glorioso Islands or Mayotte).ti,ab. | 33 |
| 168 | Congo/ | 0 |
| 169 | (Congo not ((Democratic Republic adj3 Congo) or congo red or crimean-congo)).ti,ab. | 350 |
| 170 | Cote d'Ivoire/ | 0 |
| 171 | (Cote d'Ivoire or Cote dIvoire or Ivory Coast).ti,ab. | 437 |
| 172 | eswatini/ | 0 |
| 173 | (eSwatini or Swaziland).ti,ab. | 294 |
| 174 | Ghana/ | 0 |
| 175 | (Ghana or Gold Coast).ti,ab. | 3253 |
| 176 | Kenya/ | 0 |
| 177 | (Kenya or East Africa Protectorate).ti,ab. | 3890 |
| 178 | Lesotho/ | 0 |
| 179 | (Lesotho or Basutoland).ti,ab. | 239 |
| 180 | Mauritania/ | 0 |
| 181 | Mauritania.ti,ab. | 56 |
| 182 | Nigeria/ | 0 |
| 183 | Nigeria.ti,ab. | 5590 |
| 184 | "sao tome and principe"/ | 0 |
| 185 | (Sao Tome adj2 Principe).ti,ab. | 10 |
| 186 | Senegal/ | 0 |
| 187 | Senegal.ti,ab. | 619 |
| 188 | Sudan/ | 0 |
| 189 | (Sudan not South Sudan).ti,ab. | 515 |
| 190 | Zambia/ | 0 |
| 191 | (Zambia or Northern Rhodesia).ti,ab. | 1044 |
| 192 | Zimbabwe/ | 0 |
| 193 | (Zimbabwe or Southern Rhodesia).ti,ab. | 1314 |
| 194 | American Samoa/ | 0 |
| 195 | American Samoa.ti,ab. | 92 |
| 196 | china/ or guangxi/ or inner mongolia/ or macao/ or ningxia/ or tibet/ or xinjiang/ | 0 |
| 197 | China.ti,ab. | 36698 |
| 198 | Fiji/ | 0 |
| 199 | Fiji.ti,ab. | 386 |
| 200 | exp Malaysia/ | 0 |
| 201 | (Malaysia or Malayan Union or Malaya).ti,ab. | 3815 |
| 202 | marshall islands/ | 0 |
| 203 | Marshall Islands.ti,ab. | 56 |
| 204 | nauru/ | 0 |
| 205 | Nauru.ti,ab. | 20 |
| 206 | Samoa/ | 0 |
| 207 | ((Samoa not American Samoa) or Western Samoa or Navigator Islands or Samoan Islands).ti,ab. | 257 |
| 208 | Thailand/ | 0 |
| 209 | (Thailand or Siam).ti,ab. | 4075 |
| 210 | Tonga/ | 0 |
| 211 | Tonga.ti,ab. | 141 |
| 212 | tuvalu/ | 0 |
| 213 | (Tuvalu or Ellice Islands).ti,ab. | 27 |
| 214 | Albania/ | 0 |
| 215 | Albania.ti,ab. | 274 |
| 216 | Armenia/ | 0 |
| 217 | Armenia.ti,ab. | 286 |
| 218 | exp Azerbaijan/ | 0 |
| 219 | Azerbaijan.ti,ab. | 186 |
| 220 | Belarus/ | 0 |
| 221 | (Belarus or Byelarus or Byelorussia or Belorussia).ti,ab. | 254 |
| 222 | exp "Bosnia and Herzegovina"/ | 0 |
| 223 | (Bosnia or Herzegovina).ti,ab. | 906 |
| 224 | Bulgaria/ | 0 |
| 225 | Bulgaria.ti,ab. | 767 |
| 226 | exp "Georgia (republic)"/ | 0 |
| 227 | Georgia.ti,ab. not "georgia (u.s.)"/ | 4397 |
| 228 | Kazakhstan/ | 0 |
| 229 | (Kazakhstan or Kazakh).ti,ab. | 419 |
| 230 | Kosovo/ | 0 |
| 231 | Kosovo.ti,ab. | 405 |
| 232 | "Montenegro (republic)"/ | 0 |
| 233 | Montenegro.ti,ab. | 104 |
| 234 | "republic of north macedonia"/ | 0 |
| 235 | North Macedonia.ti,ab. | 32 |
| 236 | Romania/ | 0 |
| 237 | Romania.ti,ab. | 1580 |
| 238 | exp Russian Federation/ | 0 |
| 239 | ussr/ | 0 |
| 240 | (Russia or Russian Federation or USSR or Union of Soviet Socialist Republics or Soviet Union).ti,ab. | 8262 |
| 241 | exp Serbia/ | 0 |
| 242 | Serbia.ti,ab. | 935 |
| 243 | "Turkey (republic)"/ | 0 |
| 244 | (Turkey.ti,ab. not "Turkey (bird)"/) or (Anatolia or Asia Minor).ti,ab. | 9062 |
| 245 | Turkmenistan/ | 0 |
| 246 | Turkmenistan.ti,ab. | 34 |
| 247 | Argentina/ | 0 |
| 248 | (Argentina or Argentine Republic).ti,ab. | 3059 |
| 249 | Belize/ | 0 |
| 250 | (Belize or British Honduras).ti,ab. | 281 |
| 251 | exp Brazil/ | 0 |
| 252 | Brazil.ti,ab. | 12516 |
| 253 | Colombia/ | 0 |
| 254 | Colombia.ti,ab. | 3288 |
| 255 | Costa Rica/ | 0 |
| 256 | Costa Rica.ti,ab. | 879 |
| 257 | Cuba/ | 27099 |
| 258 | Cuba.ti,ab. | 904 |
| 259 | Dominica/ | 0 |
| 260 | Dominica.ti,ab. | 67 |
| 261 | Dominican Republic/ | 0 |
| 262 | Dominican Republic.ti,ab. | 663 |
| 263 | Ecuador/ | 0 |
| 264 | Ecuador.ti,ab. | 965 |
| 265 | Grenada/ | 0 |
| 266 | Grenada.ti,ab. | 107 |
| 267 | Guatemala/ | 0 |
| 268 | Guatemala.ti,ab. | 1083 |
| 269 | Guyana/ | 0 |
| 270 | (Guyana or British Guiana).ti,ab. | 226 |
| 271 | Jamaica/ | 0 |
| 272 | Jamaica.ti,ab. | 1035 |
| 273 | exp Mexico/ | 0 |
| 274 | (Mexico or United Mexican States).ti,ab. | 11468 |
| 275 | Paraguay/ | 0 |
| 276 | Paraguay.mp. | 251 |
| 277 | Peru/ | 0 |
| 278 | Peru.ti,ab. | 2110 |
| 279 | Saint Lucia/ | 0 |
| 280 | (St Lucia or Saint Lucia or Iyonala or Hewanorra).ti,ab. | 85 |
| 281 | "Saint Vincent and the Grenadines"/ | 0 |
| 282 | (Saint Vincent or St Vincent or Grenadines).ti,ab. | 113 |
| 283 | Suriname/ | 0 |
| 284 | (Suriname or Dutch Guiana).ti,ab. | 106 |
| 285 | Venezuela/ | 0 |
| 286 | Venezuela.ti,ab. | 644 |
| 287 | Algeria/ | 0 |
| 288 | Algeria.ti,ab. | 369 |
| 289 | Iran/ | 0 |
| 290 | (Iran or Persia).ti,ab. | 5549 |
| 291 | exp Iraq/ | 0 |
| 292 | (Iraq or Mesopotamia).ti,ab. | 4370 |
| 293 | Jordan/ | 0 |
| 294 | Jordan.ti,ab. | 2690 |
| 295 | Lebanon/ | 0 |
| 296 | (Lebanon or Lebanese Republic).ti,ab. | 1748 |
| 297 | Libyan Arab Jamahiriya/ | 0 |
| 298 | libya.ti,ab. | 194 |
| 299 | maldives/ | 0 |
| 300 | Maldives.ti,ab. | 74 |
| 301 | Sri Lanka/ | 0 |
| 302 | (Sri Lanka or Ceylon).ti,ab. | 1474 |
| 303 | Botswana/ | 0 |
| 304 | (Botswana or Bechuanaland or Kalahari).ti,ab. | 957 |
| 305 | Equatorial Guinea/ | 0 |
| 306 | (Equatorial Guinea or Spanish Guinea).ti,ab. | 18 |
| 307 | Gabon/ | 0 |
| 308 | (Gabon or Gabonese Republic).ti,ab. | 120 |
| 309 | Mauritius/ | 0 |
| 310 | (Mauritius or Agalega Islands).ti,ab. | 229 |
| 311 | Namibia/ | 0 |
| 312 | (Namibia or German South West Africa).ti,ab. | 406 |
| 313 | South Africa/ | 0 |
| 314 | (South Africa or Cape Colony or British Bechuanaland or Boer Republics or Zululand or Transvaal or Natalia Republic or Orange Free State).ti,ab. | 11865 |
| 315 | or/24-314 [ALL LOW AND MIDDLE-INCOME COUNTRIES] | 250791 |
| 316 | developing country/ or low income country/ | 0 |
| 317 | ((developing or less* developed or under developed or underdeveloped or low* income) adj (economy or economies)).ti,ab. | 451 |
| 318 | ((developing or less* developed or under developed or underdeveloped or low* income or underserved or under served or deprived or poor*) adj (countr* or nation? or population? or world)).ti,ab. | 17036 |
| 319 | (low* adj (gdp or gnp or gross domestic or gross national)).ti,ab. | 52 |
| 320 | transitional countr*.ti,ab. | 66 |
| 321 | (third world or global south).ti,ab. | 1831 |
| 322 | North Korea/ | 0 |
| 323 | (North Korea or (Democratic People* Republic adj2 Korea)).ti,ab. | 176 |
| 324 | Haiti/ | 0 |
| 325 | (Haiti or Hayti).ti,ab. | 922 |
| 326 | Afghanistan/ | 0 |
| 327 | Afghanistan.ti,ab. | 3249 |
| 328 | Nepal/ | 0 |
| 329 | Nepal.ti,ab. | 1588 |
| 330 | Syrian Arab Republic/ | 0 |
| 331 | (Syria or Syrian Arab Republic).ti,ab. | 773 |
| 332 | Yemen/ | 0 |
| 333 | Yemen.ti,ab. | 225 |
| 334 | Tajikistan/ | 0 |
| 335 | Tajikistan.ti,ab. | 135 |
| 336 | Benin/ | 0 |
| 337 | (Benin or Dahomey).ti,ab. | 311 |
| 338 | Burkina Faso/ | 0 |
| 339 | (Burkina Faso or Burkina Fasso or Upper Volta).ti,ab. | 378 |
| 340 | Burundi/ | 0 |
| 341 | (Burundi or Ruanda-Urundi).ti,ab. | 171 |
| 342 | Central African Republic/ | 0 |
| 343 | (Central African Republic or Ubangi-Shari).ti,ab. | 85 |
| 344 | Chad/ | 0 |
| 345 | Chad.ti,ab. | 167 |
| 346 | Democratic Republic Congo/ | 0 |
| 347 | (((Democratic Republic or DR) adj2 Congo) or Congo-Kinshasa or Belgian Congo or Zaire or Congo Free State).ti,ab. | 627 |
| 348 | Eritrea/ | 0 |
| 349 | Eritrea.ti,ab. | 124 |
| 350 | Ethiopia/ | 0 |
| 351 | (Ethiopia or Abyssinia).ti,ab. | 2250 |
| 352 | Gambia/ | 0 |
| 353 | Gambia.ti,ab. | 176 |
| 354 | Guinea/ | 1966 |
| 355 | (Guinea not (New Guinea or Guinea Pig* or Guinea Fowl or Guinea-Bissau or Portuguese Guinea or Equatorial Guinea)).ti,ab. | 286 |
| 356 | Guinea-Bissau/ | 0 |
| 357 | (Guinea-Bissau or Portuguese Guinea).ti,ab. | 71 |
| 358 | Liberia/ | 0 |
| 359 | Liberia.ti,ab. | 424 |
| 360 | Madagascar/ | 0 |
| 361 | (Madagascar or Malagasy Republic).ti,ab. | 565 |
| 362 | Malawi/ | 0 |
| 363 | (Malawi or Nyasaland).ti,ab. | 1239 |
| 364 | Mali/ | 0 |
| 365 | Mali.ti,ab. | 318 |
| 366 | Mozambique/ | 0 |
| 367 | (Mozambique or Mocambique or Portuguese East Africa).ti,ab. | 564 |
| 368 | Niger/ | 0 |
| 369 | (Niger not (Aspergillus or Peptococcus or Schizothorax or Cruciferae or Gobius or Lasius or Agelastes or Melanosuchus or radish or Parastromateus or Orius or Apergillus or Parastromateus or Stomoxys)).ti,ab. | 370 |
| 370 | Rwanda/ | 0 |
| 371 | (Rwanda or Ruanda).ti,ab. | 1027 |
| 372 | Sierra Leone/ | 0 |
| 373 | (Sierra Leone or Salone).ti,ab. | 518 |
| 374 | Somalia/ | 0 |
| 375 | (Somalia or Somaliland).ti,ab. | 424 |
| 376 | south sudan/ | 0 |
| 377 | South Sudan.ti,ab. | 128 |
| 378 | Tanzania/ | 0 |
| 379 | (Tanzania or Tanganyika or Zanzibar).ti,ab. | 2392 |
| 380 | Togo/ | 0 |
| 381 | (Togo or Togolese Republic or Togoland).ti,ab. | 168 |
| 382 | Uganda/ | 0 |
| 383 | Uganda.ti,ab. | 3348 |
| 384 | or/316-383 [ALL LOW INCOME COUNTRIES] | 40902 |
| 385 | 315 or 384 | 250791 |
| 386 | 3 and 23 and 385 | 647 |
| 387 | limit 386 to (english language and yr="2000 - 2023") | 606 |

1. **Ovid Global Health**

Search date: January 24, 2023

| No | Search strategies | Results |
| --- | --- | --- |
| 1 | exp health insurance/ or exp medicare/ or exp social insurance/ or exp universal health coverage/ | 13301 |
| 2 | (Health insurance* or medical aid or medical insurance* or universal health coverage or health fund* or health financ* or social health protection* or social health insurance* or community health fund* or community-based insurance* or health financing or (health adj3 insuranc*) or disability insuranc*).ti,ab. | 18950 |
| 3 | 1 or 2 | 23191 |
| 4 | exp people with disabilities/ | 6875 |
| 5 | ((disabilit* or disable* or handicap* or function* limitation* or function* diversit* or dependen* or special need* or rare disease* or incapacity* or impairment*) adj5 (person* or people or individ* or patient* or subject* or adult* or elderly or child* or boy* or girl* or kid* or m#n or wom#n or teenager* or juvenile or adolescent*)).ti,ab. | 29446 |
| 6 | exp people with physical disabilities/ or exp cerebral palsy/ or exp spina bifida/ or exp spinal cord diseases/ or exp congenital abnormalities/ or exp muscular dystrophy/ or exp osteogenesis imperfecta/ or exp amputation/ or exp arthrogryposis/ or exp poliomyelitis/ or exp paralysis/ or exp paraplegia/ or exp spinal cord injuries/ | 46475 |
| 7 | (Physical* adj3 (impair* or deficienc* or disable* or disabilit* or handicap* or incapacit*)).ti,ab. | 3555 |
| 8 | (Cerebral pals* or spina bifida or muscular distroph* or osteogenesis imperfecta or polio or poliomyelitis or paralyz* or paralys* or tetraplegi* or quadriplegi* or paraplegi* or hemiplegi* or wheelchair user* or wheel chair user* or amput* or clubfoot or congenital hip dislocation or hereditary spastic paraplegia).ti,ab. | 29556 |
| 9 | exp hearing impairment/ or exp people with hearing impairment/ | 5923 |
| 10 | ((deaf* or deaf-blind disorder* or ((hearing or acoustic) adj3 (loss* or impair* or deficienc* or disable* or disabilit* or handicap*))) not (blinding or double blind* or triple blind*)).ti,ab. | 7527 |
| 11 | exp vision disorders/ or exp people with visual impairment/ or exp blindness/ | 7184 |
| 12 | ((Blind* or ((visual* or vision or eye or eyes) adj3 (loss* disabilit* or disorder* or impairment* or disable* or deficienc* or handicap*))) not (blinding or double blind* or triple blind*)).ti,ab. | 22340 |
| 13 | exp people with speech impairment/ | 83 |
| 14 | ((speech* or communication*) adj3 (disabilit* or disorder* or impair* or deficienc* or disable* or handicap*)).ti,ab. | 621 |
| 15 | exp intellectual disability/ or exp learning disabilities/ | 2402 |
| 16 | (((intellectual or cognition or cognitive or learning or developmental) adj3 (disabilit* or disable* or impair* or disorder* or incapacit* or handicap*)) or autism* or ADHD or attention deficit hyperactivity disorder* or attention-deficit hyperactivity disorder* or down syndrome or dementia or Alzheimer).ti,ab. | 31811 |
| 17 | exp mental disorders/ or exp people with mental disabilities/ | 100476 |
| 18 | (((Mental* or psychological* or behavior*) adj3 (disabilit* or disable* or impair* or handicap* or incapacit* or disorder* or illness* or ill* or dysfunction* or retard* or deficienc* or disease* or diagnos*)) or psychosis or psychoses or schizoaffective or schizophreniform or anxiet* or depression* or schizophrenia or bipolar or mental health condition*).ti,ab. | 90473 |
| 19 | (Magnifier* or medical device* or ((assistive or mobilit*) adj3 (device* or technolog* or product* or equipment* or tool*)) or hearing aid* or wheelchair* or wheel chair* or orthotic* or prosthetic* or crutch*).ti,ab. | 6724 |
| 20 | ((physical* or mental* or cognitive* or occupation* or speech* or voice* or vocation*) adj3 (rehabilitat* or therap*)).ti,ab. | 4876 |
| 21 | 4 or 5 or 6 or 7 or 8 or 9 or 10 or 11 or 12 or 13 or 14 or 15 or 16 or 17 or 18 or 19 or 20 | 257172 |
| 22 | developing country/ or low income country/ or middle income country/ | 0 |
| 23 | ((developing or less* developed or under developed or underdeveloped or middle income or low* income) adj (economy or economies)).ti,ab. | 451 |
| 24 | ((developing or less* developed or under developed or underdeveloped or middle income or low* income or underserved or under served or deprived or poor*) adj (countr* or nation? or population? or world)).ti,ab. | 72152 |
| 25 | (low* adj (gdp or gnp or gross domestic or gross national)).ti,ab. | 107 |
| 26 | (low adj3 middle adj3 countr*).ti,ab. | 14653 |
| 27 | (lmic or lmics or third world or lami countr*).ti,ab. | 5806 |
| 28 | transitional countr*.ti,ab. | 109 |
| 29 | global south.ti,ab. | 496 |
| 30 | "Africa south of the Sahara"/ | 254588 |
| 31 | ("Africa South of the Sahara" or sub-Saharan Africa or subSaharan Africa).ti,ab. | 19118 |
| 32 | Central Africa.ti,ab. | 2774 |
| 33 | Eastern Africa.ti,ab. | 715 |
| 34 | Southern Africa.ti,ab. | 3228 |
| 35 | Western Africa.ti,ab. | 563 |
| 36 | North Korea/ | 364 |
| 37 | (North Korea or (Democratic People* Republic adj2 Korea)).ti,ab. | 265 |
| 38 | Haiti/ | 2461 |
| 39 | (Haiti or Hayti).ti,ab. | 2443 |
| 40 | Afghanistan/ | 2531 |
| 41 | Afghanistan.ti,ab. | 2735 |
| 42 | Nepal/ | 9122 |
| 43 | Nepal.ti,ab. | 8154 |
| 44 | Syrian Arab Republic/ | 2077 |
| 45 | (Syria or Syrian Arab Republic).ti,ab. | 1644 |
| 46 | Yemen/ | 1993 |
| 47 | Yemen.ti,ab. | 1832 |
| 48 | Tajikistan/ | 870 |
| 49 | Tajikistan.ti,ab. | 495 |
| 50 | Benin/ | 3270 |
| 51 | (Benin or Dahomey).ti,ab. | 4151 |
| 52 | Burkina Faso/ | 5781 |
| 53 | (Burkina Faso or Burkina Fasso or Upper Volta).ti,ab. | 5705 |
| 54 | Burundi/ | 885 |
| 55 | (Burundi or Ruanda-Urundi).ti,ab. | 1144 |
| 56 | Central African Republic/ | 1210 |
| 57 | (Central African Republic or Ubangi-Shari).ti,ab. | 1225 |
| 58 | Chad/ | 1443 |
| 59 | Chad.ti,ab. | 1498 |
| 60 | Democratic Republic Congo/ | 0 |
| 61 | (((Democratic Republic or DR) adj2 Congo) or Congo-Kinshasa or Belgian Congo or Zaire or Congo Free State).ti,ab. | 6689 |
| 62 | Eritrea/ | 794 |
| 63 | Eritrea.ti,ab. | 749 |
| 64 | Ethiopia/ | 19646 |
| 65 | (Ethiopia or Abyssinia).ti,ab. | 18617 |
| 66 | Gambia/ | 3243 |
| 67 | Gambia.ti,ab. | 2824 |
| 68 | Guinea/ | 1499 |
| 69 | (Guinea not (New Guinea or Guinea Pig* or Guinea Fowl or Guinea-Bissau or Portuguese Guinea or Equatorial Guinea)).ti,ab. | 3292 |
| 70 | Guinea-Bissau/ | 1112 |
| 71 | (Guinea-Bissau or Portuguese Guinea).ti,ab. | 1098 |
| 72 | Liberia/ | 1894 |
| 73 | Liberia.ti,ab. | 1945 |
| 74 | Madagascar/ | 4293 |
| 75 | (Madagascar or Malagasy Republic).ti,ab. | 4281 |
| 76 | Malawi/ | 7869 |
| 77 | (Malawi or Nyasaland).ti,ab. | 7290 |
| 78 | Mali/ | 3686 |
| 79 | Mali.ti,ab. | 3592 |
| 80 | Mozambique/ | 3873 |
| 81 | (Mozambique or Mocambique or Portuguese East Africa).ti,ab. | 3820 |
| 82 | Niger/ | 1571 |
| 83 | (Niger not (Aspergillus or Peptococcus or Schizothorax or Cruciferae or Gobius or Lasius or Agelastes or Melanosuchus or radish or Parastromateus or Orius or Apergillus or Parastromateus or Stomoxys)).ti,ab. | 4040 |
| 84 | Rwanda/ | 3019 |
| 85 | (Rwanda or Ruanda).ti,ab. | 2963 |
| 86 | Sierra Leone/ | 2684 |
| 87 | (Sierra Leone or Salone).ti,ab. | 2685 |
| 88 | Somalia/ | 2065 |
| 89 | (Somalia or Somaliland).ti,ab. | 1782 |
| 90 | south sudan/ | 555 |
| 91 | South Sudan.ti,ab. | 664 |
| 92 | Tanzania/ | 15866 |
| 93 | (Tanzania or Tanganyika or Zanzibar).ti,ab. | 15090 |
| 94 | Togo/ | 1949 |
| 95 | (Togo or Togolese Republic or Togoland).ti,ab. | 1821 |
| 96 | Uganda/ | 16406 |
| 97 | Uganda.ti,ab. | 15491 |
| 98 | Cambodia/ | 3794 |
| 99 | Cambodia.ti,ab. | 3583 |
| 100 | exp Indonesia/ | 21536 |
| 101 | (Indonesia or Dutch East Indies).ti,ab. | 14219 |
| 102 | kiribati/ | 210 |
| 103 | (Kiribati or Gilbert Islands or Phoenix Islands or Line Islands).ti,ab. | 193 |
| 104 | Laos/ | 2223 |
| 105 | (Laos or (Lao adj1 Democratic Republic)).ti,ab. | 1661 |
| 106 | exp "Federated States of Micronesia"/ | 203 |
| 107 | Micronesia.ti,ab. | 379 |
| 108 | Mongolia/ | 1750 |
| 109 | Mongolia.ti,ab. | 2673 |
| 110 | Myanmar/ | 4862 |
| 111 | (Myanmar or Burma).ti,ab. | 4923 |
| 112 | Papua New Guinea/ | 3924 |
| 113 | (Papua New Guinea or German New Guinea or British New Guinea or Territory of Papua).ti,ab. | 3713 |
| 114 | Philippines/ | 9983 |
| 115 | (Philippines or Philippine Islands).ti,ab. | 8638 |
| 116 | solomon islands/ | 910 |
| 117 | Solomon Islands.ti,ab. | 877 |
| 118 | Timor-Leste/ | 365 |
| 119 | (Timor-Leste or East Timor or Portuguese Timor).ti,ab. | 464 |
| 120 | Vanuatu/ | 675 |
| 121 | (Vanuatu or New Hebrides).ti,ab. | 706 |
| 122 | Viet Nam/ | 11859 |
| 123 | (Viet Nam or Vietnam or French Indochina).ti,ab. | 10510 |
| 124 | Kyrgyzstan/ | 842 |
| 125 | (Kyrgyzstan or Kyrgyz Republic or Kirghizia or Kirghiz).ti,ab. | 599 |
| 126 | Moldova/ | 824 |
| 127 | Moldova.ti,ab. | 502 |
| 128 | exp Ukraine/ | 4351 |
| 129 | Ukraine.ti,ab. | 3001 |
| 130 | exp Uzbekistan/ | 2006 |
| 131 | Uzbekistan.ti,ab. | 1170 |
| 132 | Bolivia/ | 3040 |
| 133 | Bolivia.ti,ab. | 2817 |
| 134 | El Salvador/ | 1516 |
| 135 | El Salvador.ti,ab. | 1135 |
| 136 | Honduras/ | 1586 |
| 137 | Honduras.ti,ab. | 1623 |
| 138 | Nicaragua/ | 1592 |
| 139 | Nicaragua.ti,ab. | 1491 |
| 140 | Djibouti/ | 393 |
| 141 | (Djibouti or French Somaliland).ti,ab. | 401 |
| 142 | Egypt/ | 22100 |
| 143 | Egypt.ti,ab. | 14965 |
| 144 | Morocco/ | 8396 |
| 145 | Morocco.ti,ab. | 5885 |
| 146 | Tunisia/ | 7672 |
| 147 | Tunisia.mp. | 8006 |
| 148 | palestine/ | 2484 |
| 149 | (Gaza or West Bank or Palestine).ti,ab. | 2351 |
| 150 | Bangladesh/ | 15534 |
| 151 | Bangladesh.ti,ab. | 14430 |
| 152 | Bhutan/ | 713 |
| 153 | Bhutan.ti,ab. | 683 |
| 154 | exp India/ | 149312 |
| 155 | India.ti,ab. | 99496 |
| 156 | exp Pakistan/ | 22048 |
| 157 | Pakistan.ti,ab. | 17366 |
| 158 | Angola/ | 1686 |
| 159 | Angola.ti,ab. | 1732 |
| 160 | Cameroon/ | 8557 |
| 161 | (Cameroon or Kamerun or Cameroun).ti,ab. | 7654 |
| 162 | Cape Verde/ | 369 |
| 163 | (Cape Verde or Cabo Verde).ti,ab. | 406 |
| 164 | Comoros/ | 366 |
| 165 | (Comoros or Glorioso Islands or Mayotte).ti,ab. | 539 |
| 166 | Congo/ | 2394 |
| 167 | (Congo not ((Democratic Republic adj3 Congo) or congo red or crimean-congo)).ti,ab. | 5256 |
| 168 | Cote d'Ivoire/ | 5180 |
| 169 | (Cote d'Ivoire or Cote dIvoire or Ivory Coast).ti,ab. | 5170 |
| 170 | eswatini/ | 994 |
| 171 | (eSwatini or Swaziland).ti,ab. | 1042 |
| 172 | Ghana/ | 13136 |
| 173 | (Ghana or Gold Coast).ti,ab. | 12419 |
| 174 | Kenya/ | 21470 |
| 175 | (Kenya or East Africa Protectorate).ti,ab. | 19491 |
| 176 | Lesotho/ | 784 |
| 177 | (Lesotho or Basutoland).ti,ab. | 764 |
| 178 | Mauritania/ | 619 |
| 179 | Mauritania.ti,ab. | 675 |
| 180 | Nigeria/ | 41117 |
| 181 | Nigeria.ti,ab. | 35459 |
| 182 | "sao tome and principe"/ | 258 |
| 183 | (Sao Tome adj2 Principe).ti,ab. | 149 |
| 184 | Senegal/ | 7224 |
| 185 | Senegal.ti,ab. | 6237 |
| 186 | Sudan/ | 7486 |
| 187 | (Sudan not South Sudan).ti,ab. | 8090 |
| 188 | Zambia/ | 6352 |
| 189 | (Zambia or Northern Rhodesia).ti,ab. | 5689 |
| 190 | Zimbabwe/ | 7496 |
| 191 | (Zimbabwe or Southern Rhodesia).ti,ab. | 5588 |
| 192 | American Samoa/ | 280 |
| 193 | American Samoa.ti,ab. | 288 |
| 194 | china/ or guangxi/ or inner mongolia/ or macao/ or ningxia/ or tibet/ or xinjiang/ | 235664 |
| 195 | China.ti,ab. | 123346 |
| 196 | Fiji/ | 1436 |
| 197 | Fiji.ti,ab. | 1427 |
| 198 | exp Malaysia/ | 20186 |
| 199 | (Malaysia or Malayan Union or Malaya).ti,ab. | 15498 |
| 200 | marshall islands/ | 195 |
| 201 | Marshall Islands.ti,ab. | 179 |
| 202 | nauru/ | 149 |
| 203 | Nauru.ti,ab. | 152 |
| 204 | Samoa/ | 506 |
| 205 | ((Samoa not American Samoa) or Western Samoa or Navigator Islands or Samoan Islands).ti,ab. | 682 |
| 206 | Thailand/ | 28609 |
| 207 | (Thailand or Siam).ti,ab. | 22690 |
| 208 | Tonga/ | 350 |
| 209 | Tonga.ti,ab. | 373 |
| 210 | tuvalu/ | 113 |
| 211 | (Tuvalu or Ellice Islands).ti,ab. | 99 |
| 212 | Albania/ | 1304 |
| 213 | Albania.ti,ab. | 1103 |
| 214 | Armenia/ | 941 |
| 215 | Armenia.ti,ab. | 799 |
| 216 | exp Azerbaijan/ | 1429 |
| 217 | Azerbaijan.ti,ab. | 1277 |
| 218 | Belarus/ | 1201 |
| 219 | (Belarus or Byelarus or Byelorussia or Belorussia).ti,ab. | 1066 |
| 220 | exp "Bosnia and Herzegovina"/ | 1986 |
| 221 | (Bosnia or Herzegovina).ti,ab. | 1437 |
| 222 | Bulgaria/ | 6002 |
| 223 | Bulgaria.ti,ab. | 3950 |
| 224 | exp "Georgia (republic)"/ | 0 |
| 225 | Georgia.ti,ab. not "georgia (u.s.)"/ | 5646 |
| 226 | Kazakhstan/ | 3404 |
| 227 | (Kazakhstan or Kazakh).ti,ab. | 2909 |
| 228 | Kosovo/ | 495 |
| 229 | Kosovo.ti,ab. | 616 |
| 230 | "Montenegro (republic)"/ | 0 |
| 231 | Montenegro.ti,ab. | 846 |
| 232 | "republic of north macedonia"/ | 1571 |
| 233 | North Macedonia.ti,ab. | 76 |
| 234 | Romania/ | 10723 |
| 235 | Romania.ti,ab. | 5321 |
| 236 | exp Russian Federation/ | 20774 |
| 237 | ussr/ | 21096 |
| 238 | (Russia or Russian Federation or USSR or Union of Soviet Socialist Republics or Soviet Union).ti,ab. | 19641 |
| 239 | exp Serbia/ | 4731 |
| 240 | Serbia.ti,ab. | 3463 |
| 241 | "Turkey (republic)"/ | 0 |
| 242 | (Turkey.ti,ab. not "Turkey (bird)"/) or (Anatolia or Asia Minor).ti,ab. | 26713 |
| 243 | Turkmenistan/ | 676 |
| 244 | Turkmenistan.ti,ab. | 314 |
| 245 | Argentina/ | 15831 |
| 246 | (Argentina or Argentine Republic).ti,ab. | 11523 |
| 247 | Belize/ | 630 |
| 248 | (Belize or British Honduras).ti,ab. | 653 |
| 249 | exp Brazil/ | 112259 |
| 250 | Brazil.ti,ab. | 77667 |
| 251 | Colombia/ | 13007 |
| 252 | Colombia.ti,ab. | 11159 |
| 253 | Costa Rica/ | 3374 |
| 254 | Costa Rica.ti,ab. | 3217 |
| 255 | Cuba/ | 5891 |
| 256 | Cuba.ti,ab. | 4586 |
| 257 | Dominica/ | 196 |
| 258 | Dominica.ti,ab. | 353 |
| 259 | Dominican Republic/ | 1297 |
| 260 | Dominican Republic.ti,ab. | 1340 |
| 261 | Ecuador/ | 4253 |
| 262 | Ecuador.ti,ab. | 3893 |
| 263 | Grenada/ | 315 |
| 264 | Grenada.ti,ab. | 318 |
| 265 | Guatemala/ | 4272 |
| 266 | Guatemala.ti,ab. | 3812 |
| 267 | Guyana/ | 1216 |
| 268 | (Guyana or British Guiana).ti,ab. | 1299 |
| 269 | Jamaica/ | 2947 |
| 270 | Jamaica.ti,ab. | 2749 |
| 271 | exp Mexico/ | 28377 |
| 272 | (Mexico or United Mexican States).ti,ab. | 25849 |
| 273 | Paraguay/ | 1499 |
| 274 | Paraguay.mp. | 1755 |
| 275 | Peru/ | 10273 |
| 276 | Peru.ti,ab. | 8572 |
| 277 | Saint Lucia/ | 423 |
| 278 | (St Lucia or Saint Lucia or Iyonala or Hewanorra).ti,ab. | 498 |
| 279 | "Saint Vincent and the Grenadines"/ | 187 |
| 280 | (Saint Vincent or St Vincent or Grenadines).ti,ab. | 256 |
| 281 | Suriname/ | 1126 |
| 282 | (Suriname or Dutch Guiana).ti,ab. | 598 |
| 283 | Venezuela/ | 7350 |
| 284 | Venezuela.ti,ab. | 6727 |
| 285 | Algeria/ | 5954 |
| 286 | Algeria.ti,ab. | 4622 |
| 287 | Iran/ | 62752 |
| 288 | (Iran or Persia).ti,ab. | 38560 |
| 289 | exp Iraq/ | 6808 |
| 290 | (Iraq or Mesopotamia).ti,ab. | 5309 |
| 291 | Jordan/ | 4107 |
| 292 | Jordan.ti,ab. | 4274 |
| 293 | Lebanon/ | 3517 |
| 294 | (Lebanon or Lebanese Republic).ti,ab. | 2870 |
| 295 | Libyan Arab Jamahiriya/ | 1612 |
| 296 | libya.ti,ab. | 1121 |
| 297 | maldives/ | 294 |
| 298 | Maldives.ti,ab. | 262 |
| 299 | Sri Lanka/ | 7951 |
| 300 | (Sri Lanka or Ceylon).ti,ab. | 7562 |
| 301 | Botswana/ | 2351 |
| 302 | (Botswana or Bechuanaland or Kalahari).ti,ab. | 2339 |
| 303 | Equatorial Guinea/ | 484 |
| 304 | (Equatorial Guinea or Spanish Guinea).ti,ab. | 496 |
| 305 | Gabon/ | 1946 |
| 306 | (Gabon or Gabonese Republic).ti,ab. | 1836 |
| 307 | Mauritius/ | 1068 |
| 308 | (Mauritius or Agalega Islands).ti,ab. | 1044 |
| 309 | Namibia/ | 1370 |
| 310 | (Namibia or German South West Africa).ti,ab. | 1129 |
| 311 | South Africa/ | 39481 |
| 312 | (South Africa or Cape Colony or British Bechuanaland or Boer Republics or Zululand or Transvaal or Natalia Republic or Orange Free State).ti,ab. | 29194 |
| 313 | or/22-312 [ALL LOW AND MIDDLE-INCOME COUNTRIES] | 1256005 |
| 314 | developing country/ or low income country/ | 0 |
| 315 | ((developing or less* developed or under developed or underdeveloped or low* income) adj (economy or economies)).ti,ab. | 409 |
| 316 | ((developing or less* developed or under developed or underdeveloped or low* income or underserved or under served or deprived or poor*) adj (countr* or nation? or population? or world)).ti,ab. | 57850 |
| 317 | (low* adj (gdp or gnp or gross domestic or gross national)).ti,ab. | 107 |
| 318 | transitional countr*.ti,ab. | 109 |
| 319 | (third world or global south).ti,ab. | 1919 |
| 320 | North Korea/ | 364 |
| 321 | (North Korea or (Democratic People* Republic adj2 Korea)).ti,ab. | 265 |
| 322 | Haiti/ | 2461 |
| 323 | (Haiti or Hayti).ti,ab. | 2443 |
| 324 | Afghanistan/ | 2531 |
| 325 | Afghanistan.ti,ab. | 2735 |
| 326 | Nepal/ | 9122 |
| 327 | Nepal.ti,ab. | 8154 |
| 328 | Syrian Arab Republic/ | 2077 |
| 329 | (Syria or Syrian Arab Republic).ti,ab. | 1644 |
| 330 | Yemen/ | 1993 |
| 331 | Yemen.ti,ab. | 1832 |
| 332 | Tajikistan/ | 870 |
| 333 | Tajikistan.ti,ab. | 495 |
| 334 | Benin/ | 3270 |
| 335 | (Benin or Dahomey).ti,ab. | 4151 |
| 336 | Burkina Faso/ | 5781 |
| 337 | (Burkina Faso or Burkina Fasso or Upper Volta).ti,ab. | 5705 |
| 338 | Burundi/ | 885 |
| 339 | (Burundi or Ruanda-Urundi).ti,ab. | 1144 |
| 340 | Central African Republic/ | 1210 |
| 341 | (Central African Republic or Ubangi-Shari).ti,ab. | 1225 |
| 342 | Chad/ | 1443 |
| 343 | Chad.ti,ab. | 1498 |
| 344 | Democratic Republic Congo/ | 0 |
| 345 | (((Democratic Republic or DR) adj2 Congo) or Congo-Kinshasa or Belgian Congo or Zaire or Congo Free State).ti,ab. | 6689 |
| 346 | Eritrea/ | 794 |
| 347 | Eritrea.ti,ab. | 749 |
| 348 | Ethiopia/ | 19646 |
| 349 | (Ethiopia or Abyssinia).ti,ab. | 18617 |
| 350 | Gambia/ | 3243 |
| 351 | Gambia.ti,ab. | 2824 |
| 352 | Guinea/ | 1499 |
| 353 | (Guinea not (New Guinea or Guinea Pig* or Guinea Fowl or Guinea-Bissau or Portuguese Guinea or Equatorial Guinea)).ti,ab. | 3292 |
| 354 | Guinea-Bissau/ | 1112 |
| 355 | (Guinea-Bissau or Portuguese Guinea).ti,ab. | 1098 |
| 356 | Liberia/ | 1894 |
| 357 | Liberia.ti,ab. | 1945 |
| 358 | Madagascar/ | 4293 |
| 359 | (Madagascar or Malagasy Republic).ti,ab. | 4281 |
| 360 | Malawi/ | 7869 |
| 361 | (Malawi or Nyasaland).ti,ab. | 7290 |
| 362 | Mali/ | 3686 |
| 363 | Mali.ti,ab. | 3592 |
| 364 | Mozambique/ | 3873 |
| 365 | (Mozambique or Mocambique or Portuguese East Africa).ti,ab. | 3820 |
| 366 | Niger/ | 1571 |
| 367 | (Niger not (Aspergillus or Peptococcus or Schizothorax or Cruciferae or Gobius or Lasius or Agelastes or Melanosuchus or radish or Parastromateus or Orius or Apergillus or Parastromateus or Stomoxys)).ti,ab. | 4040 |
| 368 | Rwanda/ | 3019 |
| 369 | (Rwanda or Ruanda).ti,ab. | 2963 |
| 370 | Sierra Leone/ | 2684 |
| 371 | (Sierra Leone or Salone).ti,ab. | 2685 |
| 372 | Somalia/ | 2065 |
| 373 | (Somalia or Somaliland).ti,ab. | 1782 |
| 374 | south sudan/ | 555 |
| 375 | South Sudan.ti,ab. | 664 |
| 376 | Tanzania/ | 15866 |
| 377 | (Tanzania or Tanganyika or Zanzibar).ti,ab. | 15090 |
| 378 | Togo/ | 1949 |
| 379 | (Togo or Togolese Republic or Togoland).ti,ab. | 1821 |
| 380 | Uganda/ | 16406 |
| 381 | Uganda.ti,ab. | 15491 |
| 382 | or/314-381 [ALL LOW INCOME COUNTRIES] | 176938 |
| 383 | 313 or 382 | 1256005 |
| 384 | 3 and 21 and 383 | 518 |
| 385 | limit 384 to (english language and yr="2000 - 2023") | 432 |

1. **Ovid EconLit**

Search date: January 24, 2023

| No | Search strategies | Results |
| --- | --- | --- |
| 1 | (Health insurance or disability insurance).mp. [mp=heading words, abstract, title, country as subject] | 9459 |
| 2 | (Health insurance* or medical aid or medical insurance* or universal health coverage or health fund* or health financ* or social health protection* or social health insurance* or community health fund* or community-based insurance* or health financing or (health adj3 insuranc*) or disability insuranc*).ti,ab. | 7553 |
| 3 | 1 or 2 | 10116 |
| 4 | (Disability or disabled persons or people with disabilit*).mp. [mp=heading words, abstract, title, country as subject] | 3473 |
| 5 | ((disabilit* or disable* or handicap* or function* limitation* or function* deficienc* or dependen* or special need* or rare disease* or incapacity* or impairment*) adj5 (person* or people or individ* or patient* or subject* or adult* or elderly or child* or boy* or girl* or kid* or m#n or wom#n or teenager* or juvenile or adolescent*)).ti,ab. | 3627 |
| 6 | (cerebral palsy or spinal dysraphism or muscular dystrophies or osteogenesis imperfecta or limb deformities, congenital or amputation or amputation, traumatic or arthrogryposis or clubfoot or poliomyelitis or paralysis or spastic paraplegia, hereditary or paraplegia or quadriplegia or spinal cord injur*).mp. [mp=heading words, abstract, title, country as subject] | 168 |
| 7 | (Physical* adj3 (impair* or deficienc* or disable* or disabilit* or handicap* or incapacit*)).ti,ab. | 158 |
| 8 | (Cerebral pals* or spina bifida or muscular distroph* or osteogenesis imperfecta or polio or poliomyelitis or paralyz* or paralys* or tetraplegi* or quadriplegi* or paraplegi* or hemiplegi* or wheelchair user* or wheel chair user* or amput*).ti,ab. | 320 |
| 9 | (hearing loss or persons with hearing impairment*).mp. [mp=heading words, abstract, title, country as subject] | 20 |
| 10 | ((deaf* or deaf-blind disorder* or ((hearing or acoustic) adj3 (loss* or impair* or deficienc* or disable* or disabilit* or handicap*))) not (blinding or double blind* or triple blind*)).ti,ab. | 106 |
| 11 | (vision disorder* or visual impairment*).mp. [mp=heading words, abstract, title, country as subject] | 19 |
| 12 | ((Blind* or ((visual* or vision or eye or eyes) adj3 (loss* disabilit* or disorder* or impairment* or disable* or deficienc* or handicap*))) not (blinding or double blind* or triple blind*)).ti,ab. | 2088 |
| 13 | (speech disorder* or communication disorder*).mp. [mp=heading words, abstract, title, country as subject] | 5 |
| 14 | ((speech* or communication*) adj3 (disabilit* or disorder* or impair* or deficienc* or disable* or handicap*)).ti,ab. | 21 |
| 15 | (intellectual disabilit* or cognition disorder* or developmental disabilit* or learning disabilit*).mp. [mp=heading words, abstract, title, country as subject] | 105 |
| 16 | (((intellectual* or cognition or cognitive or learning or developmental) adj3 (disabilit* or disable* or impair* or disorder* or incapacit* or handicap*)) or autism* or ADHD or attention deficit hyperactivity disorder* or attention-deficit hyperactivity disorder* or down syndrome or dementia or Alzheimer).ti,ab. | 552 |
| 17 | (mental disorder* or mentally ill person*).mp. [mp=heading words, abstract, title, country as subject] | 195 |
| 18 | (((Mental* or psychological* or behavior*) adj3 (disabilit* or disable* or impair* or handicap* or incapacit* or disorder* or illness* or ill* or dysfunction* or retard* or deficienc* or disease* or diagnos*)) or psychosis or psychoses or schizoaffective or schizophreniform or anxiet* or depression* or schizophrenia or bipolar or mental health condition*).ti,ab. | 7752 |
| 19 | ((self-help device* or sensory aids or orthopedic equipment* or mobility aids or optical aids) not (HIV or HIV-AIDS or HIV AIDS)).mp. [mp=heading words, abstract, title, country as subject] | 1 |
| 20 | (Magnifier* or medical device* or ((assistive or mobilit*) adj3 (device* or technolog* or product* or equipment* or tool*)) or hearing aid* or wheelchair* or wheel chair* or orthotic* or prosthetic*).ti,ab. | 622 |
| 21 | (Rehabilitation* or physiotherap*).mp. [mp=heading words, abstract, title, country as subject] | 1404 |
| 22 | ((physical* or mental* or cognitive* or occupation* or speech* or voice* or vocation* or behavi?r) adj3 (rehabilitat* or therap*)).ti,ab. | 217 |
| 23 | 4 or 5 or 6 or 7 or 8 or 9 or 10 or 11 or 12 or 13 or 14 or 15 or 16 or 17 or 18 or 19 or 20 or 21 or 22 | 18066 |
| 24 | developing country/ or low income country/ or middle income country/ | 0 |
| 25 | ((developing or less* developed or under developed or underdeveloped or middle income or low* income) adj (economy or economies)).ti,ab. | 5499 |
| 26 | ((developing or less* developed or under developed or underdeveloped or middle income or low* income or underserved or under served or deprived or poor*) adj (countr* or nation? or population? or world)).ti,ab. | 47444 |
| 27 | (low* adj (gdp or gnp or gross domestic or gross national)).ti,ab. | 220 |
| 28 | (low adj3 middle adj3 countr*).ti,ab. | 1487 |
| 29 | (lmic or lmics or third world or lami countr*).ti,ab. | 1981 |
| 30 | transitional countr*.ti,ab. | 235 |
| 31 | global south.ti,ab. | 1001 |
| 32 | "Africa south of the Sahara"/ | 0 |
| 33 | ("Africa South of the Sahara" or sub-Saharan Africa or subSaharan Africa).ti,ab. | 5965 |
| 34 | Central Africa.ti,ab. | 233 |
| 35 | Eastern Africa.ti,ab. | 106 |
| 36 | Southern Africa.ti,ab. | 1029 |
| 37 | Western Africa.ti,ab. | 54 |
| 38 | North Korea/ | 0 |
| 39 | (North Korea or (Democratic People* Republic adj2 Korea)).ti,ab. | 380 |
| 40 | Haiti/ | 0 |
| 41 | (Haiti or Hayti).ti,ab. | 353 |
| 42 | Afghanistan/ | 0 |
| 43 | Afghanistan.ti,ab. | 690 |
| 44 | Nepal/ | 0 |
| 45 | Nepal.ti,ab. | 1447 |
| 46 | Syrian Arab Republic/ | 0 |
| 47 | (Syria or Syrian Arab Republic).ti,ab. | 440 |
| 48 | Yemen/ | 0 |
| 49 | Yemen.ti,ab. | 232 |
| 50 | Tajikistan/ | 0 |
| 51 | Tajikistan.ti,ab. | 288 |
| 52 | Benin/ | 0 |
| 53 | (Benin or Dahomey).ti,ab. | 425 |
| 54 | Burkina Faso/ | 0 |
| 55 | (Burkina Faso or Burkina Fasso or Upper Volta).ti,ab. | 778 |
| 56 | Burundi/ | 0 |
| 57 | (Burundi or Ruanda-Urundi).ti,ab. | 243 |
| 58 | Central African Republic/ | 0 |
| 59 | (Central African Republic or Ubangi-Shari).ti,ab. | 56 |
| 60 | Chad/ | 0 |
| 61 | Chad.ti,ab. | 172 |
| 62 | Democratic Republic Congo/ | 0 |
| 63 | (((Democratic Republic or DR) adj2 Congo) or Congo-Kinshasa or Belgian Congo or Zaire or Congo Free State).ti,ab. | 400 |
| 64 | Eritrea/ | 0 |
| 65 | Eritrea.ti,ab. | 114 |
| 66 | Ethiopia/ | 0 |
| 67 | (Ethiopia or Abyssinia).ti,ab. | 2608 |
| 68 | Gambia/ | 0 |
| 69 | Gambia.ti,ab. | 196 |
| 70 | Guinea/ | 0 |
| 71 | (Guinea not (New Guinea or Guinea Pig* or Guinea Fowl or Guinea-Bissau or Portuguese Guinea or Equatorial Guinea)).ti,ab. | 199 |
| 72 | Guinea-Bissau/ | 0 |
| 73 | (Guinea-Bissau or Portuguese Guinea).ti,ab. | 82 |
| 74 | Liberia/ | 0 |
| 75 | Liberia.ti,ab. | 290 |
| 76 | Madagascar/ | 0 |
| 77 | (Madagascar or Malagasy Republic).ti,ab. | 550 |
| 78 | Malawi/ | 0 |
| 79 | (Malawi or Nyasaland).ti,ab. | 1360 |
| 80 | Mali/ | 0 |
| 81 | Mali.ti,ab. | 691 |
| 82 | Mozambique/ | 0 |
| 83 | (Mozambique or Mocambique or Portuguese East Africa).ti,ab. | 1030 |
| 84 | Niger/ | 0 |
| 85 | (Niger not (Aspergillus or Peptococcus or Schizothorax or Cruciferae or Gobius or Lasius or Agelastes or Melanosuchus or radish or Parastromateus or Orius or Apergillus or Parastromateus or Stomoxys)).ti,ab. | 529 |
| 86 | Rwanda/ | 0 |
| 87 | (Rwanda or Ruanda).ti,ab. | 760 |
| 88 | Sierra Leone/ | 0 |
| 89 | (Sierra Leone or Salone).ti,ab. | 447 |
| 90 | Somalia/ | 0 |
| 91 | (Somalia or Somaliland).ti,ab. | 223 |
| 92 | south sudan/ | 0 |
| 93 | South Sudan.ti,ab. | 92 |
| 94 | Tanzania/ | 0 |
| 95 | (Tanzania or Tanganyika or Zanzibar).ti,ab. | 2579 |
| 96 | Togo/ | 0 |
| 97 | (Togo or Togolese Republic or Togoland).ti,ab. | 190 |
| 98 | Uganda/ | 0 |
| 99 | Uganda.ti,ab. | 2215 |
| 100 | Cambodia/ | 0 |
| 101 | Cambodia.ti,ab. | 785 |
| 102 | exp Indonesia/ | 0 |
| 103 | (Indonesia or Dutch East Indies).ti,ab. | 7487 |
| 104 | kiribati/ | 0 |
| 105 | (Kiribati or Gilbert Islands or Phoenix Islands or Line Islands).ti,ab. | 48 |
| 106 | Laos/ | 0 |
| 107 | (Laos or (Lao adj1 Democratic Republic)).ti,ab. | 331 |
| 108 | exp "Federated States of Micronesia"/ | 0 |
| 109 | Micronesia.ti,ab. | 33 |
| 110 | Mongolia/ | 0 |
| 111 | Mongolia.ti,ab. | 454 |
| 112 | Myanmar/ | 0 |
| 113 | (Myanmar or Burma).ti,ab. | 635 |
| 114 | Papua New Guinea/ | 0 |
| 115 | (Papua New Guinea or German New Guinea or British New Guinea or Territory of Papua).ti,ab. | 548 |
| 116 | Philippines/ | 0 |
| 117 | (Philippines or Philippine Islands).ti,ab. | 3864 |
| 118 | solomon islands/ | 0 |
| 119 | Solomon Islands.ti,ab. | 164 |
| 120 | Timor-Leste/ | 0 |
| 121 | (Timor-Leste or East Timor or Portuguese Timor).ti,ab. | 172 |
| 122 | Vanuatu/ | 0 |
| 123 | (Vanuatu or New Hebrides).ti,ab. | 130 |
| 124 | Viet Nam/ | 0 |
| 125 | (Viet Nam or Vietnam or French Indochina).ti,ab. | 4431 |
| 126 | Kyrgyzstan/ | 0 |
| 127 | (Kyrgyzstan or Kyrgyz Republic or Kirghizia or Kirghiz).ti,ab. | 512 |
| 128 | Moldova/ | 0 |
| 129 | Moldova.ti,ab. | 347 |
| 130 | exp Ukraine/ | 0 |
| 131 | Ukraine.ti,ab. | 3320 |
| 132 | exp Uzbekistan/ | 0 |
| 133 | Uzbekistan.ti,ab. | 406 |
| 134 | Bolivia/ | 0 |
| 135 | Bolivia.ti,ab. | 1214 |
| 136 | El Salvador/ | 0 |
| 137 | El Salvador.ti,ab. | 509 |
| 138 | Honduras/ | 0 |
| 139 | Honduras.ti,ab. | 477 |
| 140 | Nicaragua/ | 0 |
| 141 | Nicaragua.ti,ab. | 700 |
| 142 | Djibouti/ | 0 |
| 143 | (Djibouti or French Somaliland).ti,ab. | 53 |
| 144 | Egypt/ | 0 |
| 145 | Egypt.ti,ab. | 2421 |
| 146 | Morocco/ | 0 |
| 147 | Morocco.ti,ab. | 1292 |
| 148 | Tunisia/ | 0 |
| 149 | Tunisia.mp. | 2086 |
| 150 | palestine/ | 0 |
| 151 | (Gaza or West Bank or Palestine).ti,ab. | 535 |
| 152 | Bangladesh/ | 0 |
| 153 | Bangladesh.ti,ab. | 4643 |
| 154 | Bhutan/ | 0 |
| 155 | Bhutan.ti,ab. | 163 |
| 156 | exp India/ | 0 |
| 157 | India.ti,ab. | 27092 |
| 158 | exp Pakistan/ | 0 |
| 159 | Pakistan.ti,ab. | 7557 |
| 160 | Angola/ | 0 |
| 161 | Angola.ti,ab. | 313 |
| 162 | Cameroon/ | 0 |
| 163 | (Cameroon or Kamerun or Cameroun).ti,ab. | 935 |
| 164 | Cape Verde/ | 0 |
| 165 | (Cape Verde or Cabo Verde).ti,ab. | 97 |
| 166 | Comoros/ | 0 |
| 167 | (Comoros or Glorioso Islands or Mayotte).ti,ab. | 24 |
| 168 | Congo/ | 0 |
| 169 | (Congo not ((Democratic Republic adj3 Congo) or congo red or crimean-congo)).ti,ab. | 235 |
| 170 | Cote d'Ivoire/ | 0 |
| 171 | (Cote d'Ivoire or Cote dIvoire or Ivory Coast).ti,ab. | 850 |
| 172 | eswatini/ | 0 |
| 173 | (eSwatini or Swaziland).ti,ab. | 197 |
| 174 | Ghana/ | 0 |
| 175 | (Ghana or Gold Coast).ti,ab. | 3764 |
| 176 | Kenya/ | 0 |
| 177 | (Kenya or East Africa Protectorate).ti,ab. | 3435 |
| 178 | Lesotho/ | 0 |
| 179 | (Lesotho or Basutoland).ti,ab. | 289 |
| 180 | Mauritania/ | 0 |
| 181 | Mauritania.ti,ab. | 106 |
| 182 | Nigeria/ | 0 |
| 183 | Nigeria.ti,ab. | 4627 |
| 184 | "sao tome and principe"/ | 0 |
| 185 | (Sao Tome adj2 Principe).ti,ab. | 39 |
| 186 | Senegal/ | 0 |
| 187 | Senegal.ti,ab. | 938 |
| 188 | Sudan/ | 0 |
| 189 | (Sudan not South Sudan).ti,ab. | 615 |
| 190 | Zambia/ | 0 |
| 191 | (Zambia or Northern Rhodesia).ti,ab. | 1248 |
| 192 | Zimbabwe/ | 0 |
| 193 | (Zimbabwe or Southern Rhodesia).ti,ab. | 1378 |
| 194 | American Samoa/ | 0 |
| 195 | American Samoa.ti,ab. | 17 |
| 196 | china/ or guangxi/ or inner mongolia/ or macao/ or ningxia/ or tibet/ or xinjiang/ | 0 |
| 197 | China.ti,ab. | 47177 |
| 198 | Fiji/ | 0 |
| 199 | Fiji.ti,ab. | 519 |
| 200 | exp Malaysia/ | 0 |
| 201 | (Malaysia or Malayan Union or Malaya).ti,ab. | 5814 |
| 202 | marshall islands/ | 0 |
| 203 | Marshall Islands.ti,ab. | 20 |
| 204 | nauru/ | 0 |
| 205 | Nauru.ti,ab. | 21 |
| 206 | Samoa/ | 0 |
| 207 | ((Samoa not American Samoa) or Western Samoa or Navigator Islands or Samoan Islands).ti,ab. | 95 |
| 208 | Thailand/ | 0 |
| 209 | (Thailand or Siam).ti,ab. | 4951 |
| 210 | Tonga/ | 0 |
| 211 | Tonga.ti,ab. | 108 |
| 212 | tuvalu/ | 0 |
| 213 | (Tuvalu or Ellice Islands).ti,ab. | 32 |
| 214 | Albania/ | 0 |
| 215 | Albania.ti,ab. | 745 |
| 216 | Armenia/ | 0 |
| 217 | Armenia.ti,ab. | 382 |
| 218 | exp Azerbaijan/ | 0 |
| 219 | Azerbaijan.ti,ab. | 427 |
| 220 | Belarus/ | 0 |
| 221 | (Belarus or Byelarus or Byelorussia or Belorussia).ti,ab. | 463 |
| 222 | exp "Bosnia and Herzegovina"/ | 0 |
| 223 | (Bosnia or Herzegovina).ti,ab. | 943 |
| 224 | Bulgaria/ | 0 |
| 225 | Bulgaria.ti,ab. | 2573 |
| 226 | exp "Georgia (republic)"/ | 0 |
| 227 | Georgia.ti,ab. not "georgia (u.s.)"/ | 0 |
| 228 | Kazakhstan/ | 0 |
| 229 | (Kazakhstan or Kazakh).ti,ab. | 1088 |
| 230 | Kosovo/ | 0 |
| 231 | Kosovo.ti,ab. | 370 |
| 232 | "Montenegro (republic)"/ | 0 |
| 233 | Montenegro.ti,ab. | 361 |
| 234 | "republic of north macedonia"/ | 0 |
| 235 | North Macedonia.ti,ab. | 65 |
| 236 | Romania/ | 0 |
| 237 | Romania.ti,ab. | 3695 |
| 238 | exp Russian Federation/ | 0 |
| 239 | ussr/ | 0 |
| 240 | (Russia or Russian Federation or USSR or Union of Soviet Socialist Republics or Soviet Union).ti,ab. | 13125 |
| 241 | exp Serbia/ | 0 |
| 242 | Serbia.ti,ab. | 1472 |
| 243 | "Turkey (republic)"/ | 0 |
| 244 | (Turkey.ti,ab. not "Turkey (bird)"/) or (Anatolia or Asia Minor).ti,ab. | 0 |
| 245 | Turkmenistan/ | 0 |
| 246 | Turkmenistan.ti,ab. | 147 |
| 247 | Argentina/ | 0 |
| 248 | (Argentina or Argentine Republic).ti,ab. | 5379 |
| 249 | Belize/ | 0 |
| 250 | (Belize or British Honduras).ti,ab. | 85 |
| 251 | exp Brazil/ | 0 |
| 252 | Brazil.ti,ab. | 12326 |
| 253 | Colombia/ | 0 |
| 254 | Colombia.ti,ab. | 5167 |
| 255 | Costa Rica/ | 0 |
| 256 | Costa Rica.ti,ab. | 1082 |
| 257 | Cuba/ | 0 |
| 258 | Cuba.ti,ab. | 726 |
| 259 | Dominica/ | 0 |
| 260 | Dominica.ti,ab. | 38 |
| 261 | Dominican Republic/ | 0 |
| 262 | Dominican Republic.ti,ab. | 527 |
| 263 | Ecuador/ | 0 |
| 264 | Ecuador.ti,ab. | 1283 |
| 265 | Grenada/ | 0 |
| 266 | Grenada.ti,ab. | 73 |
| 267 | Guatemala/ | 0 |
| 268 | Guatemala.ti,ab. | 673 |
| 269 | Guyana/ | 0 |
| 270 | (Guyana or British Guiana).ti,ab. | 283 |
| 271 | Jamaica/ | 0 |
| 272 | Jamaica.ti,ab. | 945 |
| 273 | exp Mexico/ | 0 |
| 274 | (Mexico or United Mexican States).ti,ab. | 12758 |
| 275 | Paraguay/ | 0 |
| 276 | Paraguay.mp. | 471 |
| 277 | Peru/ | 0 |
| 278 | Peru.ti,ab. | 2552 |
| 279 | Saint Lucia/ | 0 |
| 280 | (St Lucia or Saint Lucia or Iyonala or Hewanorra).ti,ab. | 54 |
| 281 | "Saint Vincent and the Grenadines"/ | 0 |
| 282 | (Saint Vincent or St Vincent or Grenadines).ti,ab. | 43 |
| 283 | Suriname/ | 0 |
| 284 | (Suriname or Dutch Guiana).ti,ab. | 61 |
| 285 | Venezuela/ | 0 |
| 286 | Venezuela.ti,ab. | 1116 |
| 287 | Algeria/ | 0 |
| 288 | Algeria.ti,ab. | 695 |
| 289 | Iran/ | 0 |
| 290 | (Iran or Persia).ti,ab. | 3376 |
| 291 | exp Iraq/ | 0 |
| 292 | (Iraq or Mesopotamia).ti,ab. | 1060 |
| 293 | Jordan/ | 0 |
| 294 | Jordan.ti,ab. | 1227 |
| 295 | Lebanon/ | 0 |
| 296 | (Lebanon or Lebanese Republic).ti,ab. | 601 |
| 297 | Libyan Arab Jamahiriya/ | 0 |
| 298 | libya.ti,ab. | 273 |
| 299 | maldives/ | 0 |
| 300 | Maldives.ti,ab. | 102 |
| 301 | Sri Lanka/ | 0 |
| 302 | (Sri Lanka or Ceylon).ti,ab. | 1870 |
| 303 | Botswana/ | 0 |
| 304 | (Botswana or Bechuanaland or Kalahari).ti,ab. | 718 |
| 305 | Equatorial Guinea/ | 0 |
| 306 | (Equatorial Guinea or Spanish Guinea).ti,ab. | 35 |
| 307 | Gabon/ | 0 |
| 308 | (Gabon or Gabonese Republic).ti,ab. | 112 |
| 309 | Mauritius/ | 0 |
| 310 | (Mauritius or Agalega Islands).ti,ab. | 521 |
| 311 | Namibia/ | 0 |
| 312 | (Namibia or German South West Africa).ti,ab. | 372 |
| 313 | South Africa/ | 0 |
| 314 | (South Africa or Cape Colony or British Bechuanaland or Boer Republics or Zululand or Transvaal or Natalia Republic or Orange Free State).ti,ab. | 8477 |
| 315 | or/24-314 [ALL LOW AND MIDDLE-INCOME COUNTRIES] | 250517 |
| 316 | developing country/ or low income country/ | 0 |
| 317 | ((developing or less* developed or under developed or underdeveloped or low* income) adj (economy or economies)).ti,ab. | 5345 |
| 318 | ((developing or less* developed or under developed or underdeveloped or low* income or underserved or under served or deprived or poor*) adj (countr* or nation? or population? or world)).ti,ab. | 45617 |
| 319 | (low* adj (gdp or gnp or gross domestic or gross national)).ti,ab. | 220 |
| 320 | transitional countr*.ti,ab. | 235 |
| 321 | (third world or global south).ti,ab. | 2776 |
| 322 | North Korea/ | 0 |
| 323 | (North Korea or (Democratic People* Republic adj2 Korea)).ti,ab. | 380 |
| 324 | Haiti/ | 0 |
| 325 | (Haiti or Hayti).ti,ab. | 353 |
| 326 | Afghanistan/ | 0 |
| 327 | Afghanistan.ti,ab. | 690 |
| 328 | Nepal/ | 0 |
| 329 | Nepal.ti,ab. | 1447 |
| 330 | Syrian Arab Republic/ | 0 |
| 331 | (Syria or Syrian Arab Republic).ti,ab. | 440 |
| 332 | Yemen/ | 0 |
| 333 | Yemen.ti,ab. | 232 |
| 334 | Tajikistan/ | 0 |
| 335 | Tajikistan.ti,ab. | 288 |
| 336 | Benin/ | 0 |
| 337 | (Benin or Dahomey).ti,ab. | 425 |
| 338 | Burkina Faso/ | 0 |
| 339 | (Burkina Faso or Burkina Fasso or Upper Volta).ti,ab. | 778 |
| 340 | Burundi/ | 0 |
| 341 | (Burundi or Ruanda-Urundi).ti,ab. | 243 |
| 342 | Central African Republic/ | 0 |
| 343 | (Central African Republic or Ubangi-Shari).ti,ab. | 56 |
| 344 | Chad/ | 0 |
| 345 | Chad.ti,ab. | 172 |
| 346 | Democratic Republic Congo/ | 0 |
| 347 | (((Democratic Republic or DR) adj2 Congo) or Congo-Kinshasa or Belgian Congo or Zaire or Congo Free State).ti,ab. | 400 |
| 348 | Eritrea/ | 0 |
| 349 | Eritrea.ti,ab. | 114 |
| 350 | Ethiopia/ | 0 |
| 351 | (Ethiopia or Abyssinia).ti,ab. | 2608 |
| 352 | Gambia/ | 0 |
| 353 | Gambia.ti,ab. | 196 |
| 354 | Guinea/ | 0 |
| 355 | (Guinea not (New Guinea or Guinea Pig* or Guinea Fowl or Guinea-Bissau or Portuguese Guinea or Equatorial Guinea)).ti,ab. | 199 |
| 356 | Guinea-Bissau/ | 0 |
| 357 | (Guinea-Bissau or Portuguese Guinea).ti,ab. | 82 |
| 358 | Liberia/ | 0 |
| 359 | Liberia.ti,ab. | 290 |
| 360 | Madagascar/ | 0 |
| 361 | (Madagascar or Malagasy Republic).ti,ab. | 550 |
| 362 | Malawi/ | 0 |
| 363 | (Malawi or Nyasaland).ti,ab. | 1360 |
| 364 | Mali/ | 0 |
| 365 | Mali.ti,ab. | 691 |
| 366 | Mozambique/ | 0 |
| 367 | (Mozambique or Mocambique or Portuguese East Africa).ti,ab. | 1030 |
| 368 | Niger/ | 0 |
| 369 | (Niger not (Aspergillus or Peptococcus or Schizothorax or Cruciferae or Gobius or Lasius or Agelastes or Melanosuchus or radish or Parastromateus or Orius or Apergillus or Parastromateus or Stomoxys)).ti,ab. | 529 |
| 370 | Rwanda/ | 0 |
| 371 | (Rwanda or Ruanda).ti,ab. | 760 |
| 372 | Sierra Leone/ | 0 |
| 373 | (Sierra Leone or Salone).ti,ab. | 447 |
| 374 | Somalia/ | 0 |
| 375 | (Somalia or Somaliland).ti,ab. | 223 |
| 376 | south sudan/ | 0 |
| 377 | South Sudan.ti,ab. | 92 |
| 378 | Tanzania/ | 0 |
| 379 | (Tanzania or Tanganyika or Zanzibar).ti,ab. | 2579 |
| 380 | Togo/ | 0 |
| 381 | (Togo or Togolese Republic or Togoland).ti,ab. | 190 |
| 382 | Uganda/ | 0 |
| 383 | Uganda.ti,ab. | 2215 |
| 384 | or/316-383 [ALL LOW INCOME COUNTRIES] | 67489 |
| 385 | 315 or 384 | 250517 |
| 386 | 3 and 23 and 385 | 82 |
| 387 | limit 386 to yr="2000 - 2023" | 78 |
