# Supplementary material for: Access to health insurance amongst people with disabilities and its association with healthcare use, health status and financial protection in low- and middle-income countries: a systematic review
Source: Int J Equity Health. 2024 Dec 18;23:264. doi: 10.1186/s12939-024-02339-5 (PMC11658242; doi:10.1186/s12939-024-02339-5)
Supplement: Supplementary file 1 — Additional file 1. Search strategies. [file 12939_2024_2339_MOESM1_ESM.zip › Additional file 1. Search Strategy in Medline.docx]

Additional file 1. Search Strategy in Medline Ovid

| **No** | **Search Terms** | **Result** |
| --- | --- | --- |
| 1 | exp Insurance, health/ or exp Insurance, disability/ | 166596 |
| 2 | (Health insurance* or medical aid or medical insurance* or universal health coverage or health fund* or health financ* or social health protection* or social health insurance* or community health fund* or community-based insurance* or health financing or (health adj3 insuranc*) or disability insuranc*).ti,ab. | 64168 |
| 3 | 1 or 2 | 212720 |
| 4 | exp disabled persons/ or exp disabled children/ | 72575 |
| 5 | ((disabilit* or disable* or handicap* or function* limitation* or function* diversit* or dependen* or special need* or rare disease* or incapacity* or impairment*) adj5 (person* or people or individ* or patient* or subject* or adult* or elderly or child* or boy* or girl* or kid* or m?n or wom?n or teenager* or juvenile or adolescent*)).ti,ab. | 263467 |
| 6 | exp cerebral palsy/ or exp spinal dysraphism/ or exp osteogenesis imperfecta/ or exp limb deformities, congenital/ or exp amputation/ or exp amputation, traumatic/ or exp arthrogryposis/ or exp clubfoot/ or exp poliomyelitis/ or exp paralysis/ or exp spastic paraplegia, hereditary/ or exp paraplegia/ or exp quadriplegia/ or exp spinal cord injuries/ | 245515 |
| 7 | (Physical* adj3 (impair* or deficienc* or disable* or disabilit* or handicap* or incapacity*)).ti,ab. | 20922 |
| 8 | (Cerebral pals* or spina bifida or muscular distroph* or osteogenesis imperfecta or polio or poliomyelitis or paralyz* or paralys* or tetraplegi* or quadriplegi* or paraplegi* or hemiplegi* or wheelchair user* or wheel chair user* or amput*).ti,ab. | 201505 |
| 9 | exp hearing loss/ or exp persons with hearing impairments/ | 78014 |
| 10 | ((deaf* or deaf-blind disorder* or ((hearing or acoustic) adj3 (loss* or impair* or deficienc* or disable* or disabilit* or handicap*))) not (blinding or double blind or triple blind)).ti,ab. | 96589 |
| 11 | exp vision disorders/ | 77959 |
| 12 | ((Blind* or ((visual* or vision) adj3 (loss* disabilit* or disorder* or impairment* or disable* or deficienc* or handicap*))) not (blinding or double blind or triple blind)).ti,ab. | 195297 |
| 13 | exp speech disorders/ or exp communication disorders/ | 68334 |
| 14 | ((speech* or communication*) adj3 (disabilit* or disorder* or impair* or deficienc* or disable* or handicap*)).ti,ab. | 13370 |
| 15 | exp intellectual disability/ or exp cognition disorders/ or exp developmental disabilities/ or exp learning disabilities/ | 253718 |
| 16 | (((intellectual or cognition or cognitive or learning or developmental) adj3 (disabilit* or disable* or impair* or disorder* or incapacit* or handicap*)) or autism* or ADHD or attention deficit hyperactivity disorder* or attention-deficit hyperactivity disorder* or down syndrome or dementia or Alzheimer).ti,ab. | 384694 |
| 17 | exp mental disorders/ or exp mentally ill persons/ | 1412269 |
| 18 | (((Mental* or psychological* or behavior*) adj3 (disabilit* or disable* or impair* or handicap* or incapacit* or disorder* or illness* or ill* or dysfunction* or retard* or deficienc* or disease* or diagnos*)) or psychosis or psychoses or schizoaffective or schizophreniform or schizophrenia or bipolar or mental health condition*).ti,ab. | 401242 |
| 19 | exp self-help devices/ or exp sensory aids/ or exp orthopedic equipment/ | 147099 |
| 20 | (Magnifier* or medical device* or ((assistive or mobilit*) adj3 (device* or technolog* or product* or equipment* or tool*)) or hearing aid* or wheelchair* or wheel chair* or orthotic* or prosthetic*).ti,ab. | 109707 |
| 21 | exp rehabilitation/ | 347475 |
| 22 | ((physical* or mental* or cognitive* or occupation* or speech* or voice* or vocation*) adj3 (rehabilitat* or therap*)).ti,ab. | 95119 |
| 23 | 4 or 5 or 6 or 7 or 8 or 9 or 10 or 11 or 12 or 13 or 14 or 15 or 16 or 17 or 18 or 19 or 20 or 21 or 22 | 3031919 |
| 24 | developing country/ or low income country/ or middle income country/ | 80307 |
| 25 | ((developing or less* developed or under developed or underdeveloped or middle income or low* income) adj (economy or economies)).ti,ab. | 906 |
| 26 | ((developing or less* developed or under developed or underdeveloped or middle income or low* income or underserved or under served or deprived or poor*) adj (countr* or nation? or population? or world)).ti,ab. | 129229 |
| 27 | (low* adj (gdp or gnp or gross domestic or gross national)).ti,ab. | 338 |
| 28 | (low adj3 middle adj3 countr*).ti,ab. | 28037 |
| 29 | (lmic or lmics or third world or lami countr*).ti,ab. | 12006 |
| 30 | transitional countr*.ti,ab. | 176 |
| 31 | global south.ti,ab. | 920 |
| 32 | "Africa south of the Sahara"/ | 13050 |
| 33 | ("Africa South of the Sahara" or sub-Saharan Africa or subSaharan Africa).ti,ab. | 27675 |
| 34 | Central Africa.ti,ab. | 3672 |
| 35 | Eastern Africa.ti,ab. | 1229 |
| 36 | Southern Africa.ti,ab. | 5061 |
| 37 | Western Africa.ti,ab. | 1014 |
| 38 | North Korea/ | 289 |
| 39 | (North Korea or (Democratic People* Republic adj2 Korea)).ti,ab. | 507 |
| 40 | Haiti/ | 3544 |
| 41 | (Haiti or Hayti).ti,ab. | 3441 |
| 42 | Afghanistan/ | 3733 |
| 43 | Afghanistan.ti,ab. | 6876 |
| 44 | Nepal/ | 10445 |
| 45 | Nepal.ti,ab. | 12744 |
| 46 | Syrian Arab Republic/ | 0 |
| 47 | (Syria or Syrian Arab Republic).ti,ab. | 2618 |
| 48 | Yemen/ | 1555 |
| 49 | Yemen.ti,ab. | 2168 |
| 50 | Tajikistan/ | 802 |
| 51 | Tajikistan.ti,ab. | 738 |
| 52 | Benin/ | 1881 |
| 53 | (Benin or Dahomey).ti,ab. | 4045 |
| 54 | Burkina Faso/ | 3872 |
| 55 | (Burkina Faso or Burkina Fasso or Upper Volta).ti,ab. | 5105 |
| 56 | Burundi/ | 722 |
| 57 | (Burundi or Ruanda-Urundi).ti,ab. | 1092 |
| 58 | Central African Republic/ | 836 |
| 59 | (Central African Republic or Ubangi-Shari).ti,ab. | 1136 |
| 60 | Chad/ | 812 |
| 61 | Chad.ti,ab. | 1445 |
| 62 | Democratic Republic Congo/ | 0 |
| 63 | (((Democratic Republic or DR) adj2 Congo) or Congo-Kinshasa or Belgian Congo or Zaire or Congo Free State).ti,ab. | 5291 |
| 64 | Eritrea/ | 408 |
| 65 | Eritrea.ti,ab. | 667 |
| 66 | Ethiopia/ | 18406 |
| 67 | (Ethiopia or Abyssinia).ti,ab. | 25168 |
| 68 | Gambia/ | 2650 |
| 69 | Gambia.ti,ab. | 2628 |
| 70 | Guinea/ | 1281 |
| 71 | (Guinea not (New Guinea or Guinea Pig* or Guinea Fowl or Guinea-Bissau or Portuguese Guinea or Equatorial Guinea)).ti,ab. | 3170 |
| 72 | Guinea-Bissau/ | 1020 |
| 73 | (Guinea-Bissau or Portuguese Guinea).ti,ab. | 1154 |
| 74 | Liberia/ | 1365 |
| 75 | Liberia.ti,ab. | 1838 |
| 76 | Madagascar/ | 3872 |
| 77 | (Madagascar or Malagasy Republic).ti,ab. | 5524 |
| 78 | Malawi/ | 6571 |
| 79 | (Malawi or Nyasaland).ti,ab. | 8581 |
| 80 | Mali/ | 2658 |
| 81 | Mali.ti,ab. | 4155 |
| 82 | Mozambique/ | 2965 |
| 83 | (Mozambique or Mocambique or Portuguese East Africa).ti,ab. | 4432 |
| 84 | Niger/ | 1384 |
| 85 | (Niger not (Aspergillus or Peptococcus or Schizothorax or Cruciferae or Gobius or Lasius or Agelastes or Melanosuchus or radish or Parastromateus or Orius or Apergillus or Parastromateus or Stomoxys)).ti,ab. | 3991 |
| 86 | Rwanda/ | 3066 |
| 87 | (Rwanda or Ruanda).ti,ab. | 3943 |
| 88 | Sierra Leone/ | 1890 |
| 89 | (Sierra Leone or Salone).ti,ab. | 2765 |
| 90 | Somalia/ | 1842 |
| 91 | (Somalia or Somaliland).ti,ab. | 1879 |
| 92 | south sudan/ | 264 |
| 93 | South Sudan.ti,ab. | 746 |
| 94 | Tanzania/ | 13526 |
| 95 | (Tanzania or Tanganyika or Zanzibar).ti,ab. | 16462 |
| 96 | Togo/ | 1267 |
| 97 | (Togo or Togolese Republic or Togoland).ti,ab. | 1751 |
| 98 | Uganda/ | 14896 |
| 99 | Uganda.ti,ab. | 17908 |
| 100 | Cambodia/ | 3796 |
| 101 | Cambodia.ti,ab. | 4650 |
| 102 | exp Indonesia/ | 13237 |
| 103 | (Indonesia or Dutch East Indies).ti,ab. | 17222 |
| 104 | kiribati/ | 1263 |
| 105 | (Kiribati or Gilbert Islands or Phoenix Islands or Line Islands).ti,ab. | 289 |
| 106 | Laos/ | 2229 |
| 107 | (Laos or (Lao adj1 Democratic Republic)).ti,ab. | 2432 |
| 108 | exp "Federated States of Micronesia"/ | 0 |
| 109 | Micronesia.ti,ab. | 736 |
| 110 | Mongolia/ | 2068 |
| 111 | Mongolia.ti,ab. | 5343 |
| 112 | Myanmar/ | 3129 |
| 113 | (Myanmar or Burma).ti,ab. | 5349 |
| 114 | Papua New Guinea/ | 3714 |
| 115 | (Papua New Guinea or German New Guinea or British New Guinea or Territory of Papua).ti,ab. | 4906 |
| 116 | Philippines/ | 9446 |
| 117 | (Philippines or Philippine Islands).ti,ab. | 10300 |
| 118 | solomon islands/ | 1147 |
| 119 | Solomon Islands.ti,ab. | 931 |
| 120 | Timor-Leste/ | 263 |
| 121 | (Timor-Leste or East Timor or Portuguese Timor).ti,ab. | 632 |
| 122 | Vanuatu/ | 418 |
| 123 | (Vanuatu or New Hebrides).ti,ab. | 812 |
| 124 | Viet Nam/ | 14474 |
| 125 | (Viet Nam or Vietnam or French Indochina).ti,ab. | 19017 |
| 126 | Kyrgyzstan/ | 1388 |
| 127 | (Kyrgyzstan or Kyrgyz Republic or Kirghizia or Kirghiz).ti,ab. | 1197 |
| 128 | Moldova/ | 743 |
| 129 | Moldova.ti,ab. | 653 |
| 130 | exp Ukraine/ | 16966 |
| 131 | Ukraine.ti,ab. | 6274 |
| 132 | exp Uzbekistan/ | 1987 |
| 133 | Uzbekistan.ti,ab. | 1320 |
| 134 | Bolivia/ | 2821 |
| 135 | Bolivia.ti,ab. | 3723 |
| 136 | El Salvador/ | 947 |
| 137 | El Salvador.ti,ab. | 1462 |
| 138 | Honduras/ | 1244 |
| 139 | Honduras.ti,ab. | 2036 |
| 140 | Nicaragua/ | 1611 |
| 141 | Nicaragua.ti,ab. | 2109 |
| 142 | Djibouti/ | 250 |
| 143 | (Djibouti or French Somaliland).ti,ab. | 437 |
| 144 | Egypt/ | 17218 |
| 145 | Egypt.ti,ab. | 17278 |
| 146 | Morocco/ | 6471 |
| 147 | Morocco.ti,ab. | 6923 |
| 148 | Tunisia/ | 9181 |
| 149 | Tunisia.mp. | 11848 |
| 150 | palestine/ | 0 |
| 151 | (Gaza or West Bank or Palestine).ti,ab. | 3133 |
| 152 | Bangladesh/ | 14023 |
| 153 | Bangladesh.ti,ab. | 18207 |
| 154 | Bhutan/ | 663 |
| 155 | Bhutan.ti,ab. | 1047 |
| 156 | exp India/ | 117327 |
| 157 | India.ti,ab. | 124618 |
| 158 | exp Pakistan/ | 22042 |
| 159 | Pakistan.ti,ab. | 25331 |
| 160 | Angola/ | 1126 |
| 161 | Angola.ti,ab. | 1672 |
| 162 | Cameroon/ | 6412 |
| 163 | (Cameroon or Kamerun or Cameroun).ti,ab. | 8310 |
| 164 | Cape Verde/ | 248 |
| 165 | (Cape Verde or Cabo Verde).ti,ab. | 741 |
| 166 | Comoros/ | 373 |
| 167 | (Comoros or Glorioso Islands or Mayotte).ti,ab. | 703 |
| 168 | Congo/ | 2001 |
| 169 | (Congo not ((Democratic Republic adj3 Congo) or congo red or crimean-congo)).ti,ab. | 2918 |
| 170 | Cote d'Ivoire/ | 3505 |
| 171 | (Cote d'Ivoire or Cote dIvoire or Ivory Coast).ti,ab. | 4372 |
| 172 | eswatini/ | 745 |
| 173 | (eSwatini or Swaziland).ti,ab. | 1137 |
| 174 | Ghana/ | 10818 |
| 175 | (Ghana or Gold Coast).ti,ab. | 14309 |
| 176 | Kenya/ | 19049 |
| 177 | (Kenya or East Africa Protectorate).ti,ab. | 21771 |
| 178 | Lesotho/ | 509 |
| 179 | (Lesotho or Basutoland).ti,ab. | 890 |
| 180 | Mauritania/ | 495 |
| 181 | Mauritania.ti,ab. | 726 |
| 182 | Nigeria/ | 33358 |
| 183 | Nigeria.ti,ab. | 34789 |
| 184 | "sao tome and principe"/ | 31 |
| 185 | (Sao Tome adj2 Principe).ti,ab. | 182 |
| 186 | Senegal/ | 6176 |
| 187 | Senegal.ti,ab. | 6413 |
| 188 | Sudan/ | 5112 |
| 189 | (Sudan not South Sudan).ti,ab. | 8433 |
| 190 | Zambia/ | 5385 |
| 191 | (Zambia or Northern Rhodesia).ti,ab. | 6433 |
| 192 | Zimbabwe/ | 6496 |
| 193 | (Zimbabwe or Southern Rhodesia).ti,ab. | 6683 |
| 194 | American Samoa/ | 208 |
| 195 | American Samoa.ti,ab. | 405 |
| 196 | china/ or guangxi/ or inner mongolia/ or macao/ or ningxia/ or tibet/ or xinjiang/ | 245400 |
| 197 | China.ti,ab. | 272247 |
| 198 | Fiji/ | 1114 |
| 199 | Fiji.ti,ab. | 2219 |
| 200 | exp Malaysia/ | 17790 |
| 201 | (Malaysia or Malayan Union or Malaya).ti,ab. | 20933 |
| 202 | marshall islands/ | 1263 |
| 203 | Marshall Islands.ti,ab. | 341 |
| 204 | nauru/ | 1263 |
| 205 | Nauru.ti,ab. | 169 |
| 206 | Samoa/ | 392 |
| 207 | ((Samoa not American Samoa) or Western Samoa or Navigator Islands or Samoan Islands).ti,ab. | 667 |
| 208 | Thailand/ | 30308 |
| 209 | (Thailand or Siam).ti,ab. | 32314 |
| 210 | Tonga/ | 292 |
| 211 | Tonga.ti,ab. | 527 |
| 212 | tuvalu/ | 1263 |
| 213 | (Tuvalu or Ellice Islands).ti,ab. | 86 |
| 214 | Albania/ | 955 |
| 215 | Albania.ti,ab. | 1258 |
| 216 | Armenia/ | 1557 |
| 217 | Armenia.ti,ab. | 1339 |
| 218 | exp Azerbaijan/ | 1288 |
| 219 | Azerbaijan.ti,ab. | 1643 |
| 220 | Belarus/ | 2151 |
| 221 | (Belarus or Byelarus or Byelorussia or Belorussia).ti,ab. | 1756 |
| 222 | exp "Bosnia and Herzegovina"/ | 2336 |
| 223 | (Bosnia or Herzegovina).ti,ab. | 2710 |
| 224 | Bulgaria/ | 6638 |
| 225 | Bulgaria.ti,ab. | 4775 |
| 226 | exp "Georgia (republic)"/ | 1968 |
| 227 | Georgia.ti,ab. not "georgia (u.s.)"/ | 11659 |
| 228 | Kazakhstan/ | 3030 |
| 229 | (Kazakhstan or Kazakh).ti,ab. | 3615 |
| 230 | Kosovo/ | 298 |
| 231 | Kosovo.ti,ab. | 1091 |
| 232 | "Montenegro (republic)"/ | 0 |
| 233 | Montenegro.ti,ab. | 1007 |
| 234 | "republic of north macedonia"/ | 630 |
| 235 | North Macedonia.ti,ab. | 199 |
| 236 | Romania/ | 10885 |
| 237 | Romania.ti,ab. | 7307 |
| 238 | exp Russian Federation/ | 0 |
| 239 | ussr/ | 42848 |
| 240 | (Russia or Russian Federation or USSR or Union of Soviet Socialist Republics or Soviet Union).ti,ab. | 32524 |
| 241 | exp Serbia/ | 3694 |
| 242 | Serbia.ti,ab. | 5381 |
| 243 | "Turkey (republic)"/ | 0 |
| 244 | (Turkey.ti,ab. not "Turkey (bird)"/) or (Anatolia or Asia Minor).ti,ab. | 43319 |
| 245 | Turkmenistan/ | 590 |
| 246 | Turkmenistan.ti,ab. | 414 |
| 247 | Argentina/ | 17714 |
| 248 | (Argentina or Argentine Republic).ti,ab. | 19763 |
| 249 | Belize/ | 630 |
| 250 | (Belize or British Honduras).ti,ab. | 972 |
| 251 | exp Brazil/ | 114015 |
| 252 | Brazil.ti,ab. | 102294 |
| 253 | Colombia/ | 12873 |
| 254 | Colombia.ti,ab. | 15734 |
| 255 | Costa Rica/ | 3987 |
| 256 | Costa Rica.ti,ab. | 5566 |
| 257 | Cuba/ | 5342 |
| 258 | Cuba.ti,ab. | 5006 |
| 259 | Dominica/ | 114 |
| 260 | Dominica.ti,ab. | 556 |
| 261 | Dominican Republic/ | 1737 |
| 262 | Dominican Republic.ti,ab. | 2243 |
| 263 | Ecuador/ | 4550 |
| 264 | Ecuador.ti,ab. | 5837 |
| 265 | Grenada/ | 166 |
| 266 | Grenada.ti,ab. | 363 |
| 267 | Guatemala/ | 3300 |
| 268 | Guatemala.ti,ab. | 4091 |
| 269 | Guyana/ | 745 |
| 270 | (Guyana or British Guiana).ti,ab. | 1221 |
| 271 | Jamaica/ | 3592 |
| 272 | Jamaica.ti,ab. | 3490 |
| 273 | exp Mexico/ | 43481 |
| 274 | (Mexico or United Mexican States).ti,ab. | 49768 |
| 275 | Paraguay/ | 901 |
| 276 | Paraguay.mp. | 2048 |
| 277 | Peru/ | 10564 |
| 278 | Peru.ti,ab. | 12963 |
| 279 | Saint Lucia/ | 75 |
| 280 | (St Lucia or Saint Lucia or Iyonala or Hewanorra).ti,ab. | 372 |
| 281 | "Saint Vincent and the Grenadines"/ | 58 |
| 282 | (Saint Vincent or St Vincent or Grenadines).ti,ab. | 657 |
| 283 | Suriname/ | 1003 |
| 284 | (Suriname or Dutch Guiana).ti,ab. | 705 |
| 285 | Venezuela/ | 5122 |
| 286 | Venezuela.ti,ab. | 5735 |
| 287 | Algeria/ | 3506 |
| 288 | Algeria.ti,ab. | 3995 |
| 289 | Iran/ | 36100 |
| 290 | (Iran or Persia).ti,ab. | 49396 |
| 291 | exp Iraq/ | 5368 |
| 292 | (Iraq or Mesopotamia).ti,ab. | 8418 |
| 293 | Jordan/ | 5263 |
| 294 | Jordan.ti,ab. | 8058 |
| 295 | Lebanon/ | 5131 |
| 296 | (Lebanon or Lebanese Republic).ti,ab. | 5791 |
| 297 | Libyan Arab Jamahiriya/ | 0 |
| 298 | libya.ti,ab. | 1462 |
| 299 | maldives/ | 839 |
| 300 | Maldives.ti,ab. | 462 |
| 301 | Sri Lanka/ | 6986 |
| 302 | (Sri Lanka or Ceylon).ti,ab. | 8413 |
| 303 | Botswana/ | 2151 |
| 304 | (Botswana or Bechuanaland or Kalahari).ti,ab. | 3106 |
| 305 | Equatorial Guinea/ | 309 |
| 306 | (Equatorial Guinea or Spanish Guinea).ti,ab. | 485 |
| 307 | Gabon/ | 1618 |
| 308 | (Gabon or Gabonese Republic).ti,ab. | 1981 |
| 309 | Mauritius/ | 621 |
| 310 | (Mauritius or Agalega Islands).ti,ab. | 1148 |
| 311 | Namibia/ | 1288 |
| 312 | (Namibia or German South West Africa).ti,ab. | 1932 |
| 313 | South Africa/ | 48581 |
| 314 | (South Africa or Cape Colony or British Bechuanaland or Boer Republics or Zululand or Transvaal or Natalia Republic or Orange Free State).ti,ab. | 42394 |
| 315 | or/24-314 [ALL LOW AND MIDDLE-INCOME COUNTRIES] | 1757962 |
| 316 | developing country/ or low income country/ | 80307 |
| 317 | ((developing or less* developed or under developed or underdeveloped or low* income) adj (economy or economies)).ti,ab. | 806 |
| 318 | ((developing or less* developed or under developed or underdeveloped or low* income or underserved or under served or deprived or poor*) adj (countr* or nation? or population? or world)).ti,ab. | 101624 |
| 319 | (low* adj (gdp or gnp or gross domestic or gross national)).ti,ab. | 338 |
| 320 | transitional countr*.ti,ab. | 176 |
| 321 | (third world or global south).ti,ab. | 4063 |
| 322 | North Korea/ | 289 |
| 323 | (North Korea or (Democratic People* Republic adj2 Korea)).ti,ab. | 507 |
| 324 | Haiti/ | 3544 |
| 325 | (Haiti or Hayti).ti,ab. | 3441 |
| 326 | Afghanistan/ | 3733 |
| 327 | Afghanistan.ti,ab. | 6876 |
| 328 | Nepal/ | 10445 |
| 329 | Nepal.ti,ab. | 12744 |
| 330 | Syrian Arab Republic/ | 0 |
| 331 | (Syria or Syrian Arab Republic).ti,ab. | 2618 |
| 332 | Yemen/ | 1555 |
| 333 | Yemen.ti,ab. | 2168 |
| 334 | Tajikistan/ | 802 |
| 335 | Tajikistan.ti,ab. | 738 |
| 336 | Benin/ | 1881 |
| 337 | (Benin or Dahomey).ti,ab. | 4045 |
| 338 | Burkina Faso/ | 3872 |
| 339 | (Burkina Faso or Burkina Fasso or Upper Volta).ti,ab. | 5105 |
| 340 | Burundi/ | 722 |
| 341 | (Burundi or Ruanda-Urundi).ti,ab. | 1092 |
| 342 | Central African Republic/ | 836 |
| 343 | (Central African Republic or Ubangi-Shari).ti,ab. | 1136 |
| 344 | Chad/ | 812 |
| 345 | Chad.ti,ab. | 1445 |
| 346 | Democratic Republic Congo/ | 0 |
| 347 | (((Democratic Republic or DR) adj2 Congo) or Congo-Kinshasa or Belgian Congo or Zaire or Congo Free State).ti,ab. | 5291 |
| 348 | Eritrea/ | 408 |
| 349 | Eritrea.ti,ab. | 667 |
| 350 | Ethiopia/ | 18406 |
| 351 | (Ethiopia or Abyssinia).ti,ab. | 25168 |
| 352 | Gambia/ | 2650 |
| 353 | Gambia.ti,ab. | 2628 |
| 354 | Guinea/ | 1281 |
| 355 | (Guinea not (New Guinea or Guinea Pig* or Guinea Fowl or Guinea-Bissau or Portuguese Guinea or Equatorial Guinea)).ti,ab. | 3170 |
| 356 | Guinea-Bissau/ | 1020 |
| 357 | (Guinea-Bissau or Portuguese Guinea).ti,ab. | 1154 |
| 358 | Liberia/ | 1365 |
| 359 | Liberia.ti,ab. | 1838 |
| 360 | Madagascar/ | 3872 |
| 361 | (Madagascar or Malagasy Republic).ti,ab. | 5524 |
| 362 | Malawi/ | 6571 |
| 363 | (Malawi or Nyasaland).ti,ab. | 8581 |
| 364 | Mali/ | 2658 |
| 365 | Mali.ti,ab. | 4155 |
| 366 | Mozambique/ | 2965 |
| 367 | (Mozambique or Mocambique or Portuguese East Africa).ti,ab. | 4432 |
| 368 | Niger/ | 1384 |
| 369 | (Niger not (Aspergillus or Peptococcus or Schizothorax or Cruciferae or Gobius or Lasius or Agelastes or Melanosuchus or radish or Parastromateus or Orius or Apergillus or Parastromateus or Stomoxys)).ti,ab. | 3991 |
| 370 | Rwanda/ | 3066 |
| 371 | (Rwanda or Ruanda).ti,ab. | 3943 |
| 372 | Sierra Leone/ | 1890 |
| 373 | (Sierra Leone or Salone).ti,ab. | 2765 |
| 374 | Somalia/ | 1842 |
| 375 | (Somalia or Somaliland).ti,ab. | 1879 |
| 376 | south sudan/ | 264 |
| 377 | South Sudan.ti,ab. | 746 |
| 378 | Tanzania/ | 13526 |
| 379 | (Tanzania or Tanganyika or Zanzibar).ti,ab. | 16462 |
| 380 | Togo/ | 1267 |
| 381 | (Togo or Togolese Republic or Togoland).ti,ab. | 1751 |
| 382 | Uganda/ | 14896 |
| 383 | Uganda.ti,ab. | 17908 |
| 384 | or/316-383 [ALL LOW INCOME COUNTRIES] | 301938 |
| 385 | 315 or 384 | 1757962 |
| 386 | 3 and 23 and 385 | 972 |
| 387 | limit 386 to (yr="2000 - 2023" and english) | 840 |
