# Supplementary material for: Access to health insurance amongst people with disabilities and its association with healthcare use, health status and financial protection in low- and middle-income countries: a systematic review
Source: Int J Equity Health. 2024 Dec 18;23:264. doi: 10.1186/s12939-024-02339-5 (PMC11658242; doi:10.1186/s12939-024-02339-5)
Supplement: Supplementary file 2 — Additional file 2. Health insurance coverage/access among people with disabilities in LMICs. [file 12939_2024_2339_MOESM2_ESM.zip › Additional file 2. Health Insurance Coverage-Access V4.docx]

Additional file 2. Health Insurance Coverage/access amongst People with Disabilities in LMICs

| **Studies** | **Setting/ country** | **Study design** | **Source of data (representativeness)**  **Recruitment** | **Health Insurance,**  **Public/private** | **Sample (age in year)** | **Type of disability (measurement)** | **Comparator** | **Coverage (%)**  **OR (95% CI)** | **Association (disability vs non-disability)** |
| --- | --- | --- | --- | --- | --- | --- | --- | --- | --- |
| Agbadi (2021) | Ghana | Cross-sectional | 2017/18 Ghana Multiple Indicator Cluster Survey (G-MICS) (National)  Population-based | National Health Insurance Scheme,  Public | 30,750 children with disabilities (<18) | All types (Washington Group UNICEF Child Function Module) | Children without functional disability | Probability for **not** having health insurance: 1.19 (1.09 – 1.3) | Negative |
| Atchessi (2014) | Ougraye, Burkina Faso | Cross-sectional | Survey (district)  Registry-based | Community-based health insurance, Private | 1687 indigents – unclear definition (≥18) | 1. Vision (World Health Survey Questionnaire) 2. Physical (limitation in arm and finger movement, difficulty in walking 400 m) | 1. People without vision impairment (VI) 2. People without mobility impairment | 1. VI vs non-VI: 1.45 (1.14 – 1.84) 2. Poor mobility vs good mobility & strength: 1.09 (0.80 – 1.50) 3. Good mobility & poor strength vs good mobility & good strength: 1.73 (1.28 – 2.33) | 1. Positive 2. Null 3. Positive |
| Banks (2019) | Cam Le District, Vietnam | Case-control | Survey (district)  Population-based | Any health insurance | 222 adults and children with disabilities (all ages) | All types (Washington Group Short Set) | 222 people without disability | 1. Any health insurance: 96% vs 88.3%, AOR 2.9 (1.1-7.2) P≤0.05 2. State-subsidized health insurance: 72.7% vs 27%, AOR 7.7 (4.7-12.5) P≤0.05 | 1. Positive 2. Positive |
| Barreto (2022) | Brazil | Cross-sectional | 2013 National Health Survey (national)  Population-based | Private | 55,369 adults and older people (mean age disability: 54.4, no disability: 41.57) | Acquired physical impairment (self-reported yes/no) | People without acquired physical impairment | Coverage: 26.21% vs 30.60%  AOR for **not** having insurance: 1.06 (1.06 – 1.06) | 1. Negative |
| Bernabe-Ortiz (2016) | Morropon, Piura, Northern Peru, Peru | Case-control | Nested from a population-based survey (City)  Population-based | Comprehensive Health Insurance, Public | 141 adults (≥18) and 20 children (<18) with disabilities | All types (Washington Group Short Set) | 141 adults without disability  20 children without disability | 1. Adults: 83% vs 80%; AOR uninsured vs insured: 0.85 (0.45-1.62) 2. Children: 95% vs 100%; AOR insured vs uninsured: - | 1. Null 2. NA |
| Contentti (2019) | 12 Latin America countries | Cross-sectional | Survey in September and November of 2018 (regional)  Population-based | Any health insurance | 1469 people with multiple sclerosis (all ages) | Physical (clinical diagnosis for MS) | NA | Insured: 156 (10.6%) | NA |
| Doubova (2015) | Mexico | Cross-sectional | Mexican Survey of Health and Nutrition (ENSANUT) 2012 (national)  Population-based | 1. Social Security Health Insurance (SSHI) targeting workers, Public 2. Seguro Popular Health Insurance (SPHI) targeting rural and poor population, Public | 18,847 Older adults (≥60) | Physical and/or mental limitation for sight, hearing, walking, dressing, speaking, paying attention, understanding (self-reported) | Total population | 1. 36% (34.9-38.6) vs 51.3% (49.6-53) 2. 39.6% (37.6-41.7) vs 30.9% (29.5-32.3) | 1. Negative 2. Positive |
| Flores-flores (2018) | Peru | Cross-sectional | Peru's 2012 survey Health and Wellbeing in Older Adults (ESBAM) (national)  Population-based | 1. SIS insurance targeting people living in poverty, Public 2. EsSalud, Armed Forces (FFAA), National Police (PNP), Public | 3,869 older adults (65-80) | All types (Modified Katz Index for ADL) | Older adults without disability | 1. SIS Insurance: 64.2% vs 60.4%. APR: 1.03 (0.97 – 1.09) 2. Other insurance: 0.9% vs 2.9%. APR: 0.37 (0.16 – 0.87) | 1. Null 2. Negative |
| Gomez (2021) | Colombia | Cross-sectional | SABE (Health, well-being, and aging) Colombia Project 2014-2015 (national)  Population-based | Public mandatory health insurance, Public | 23,694 older adults (≥60) | 1. Physical (NAGI mobility) 2. All type (ADL Barthel Index Scale- ≥1) 3. All types (IADL - ≥1) | Older adults without disability | 1. Mobility disability (AOR 1.15, 95% CI 1.15-1.15) 2. ADL disability (AOR 1.10, 95% CI 1.10-1.11) 3. IADL disability (AOR 1.25, 95% CI 1,25-1.26) | 1. Positive 2. Positive 3. Positive |
| Guo (2015) | China | Cross-sectional | The Second China National Sample Survey on Disability (CNSSD) 2006 (national)  Population-based | Any health insurance | 354,859 Older adults (≥60) | All types (physician diagnosis using ICD-10, ICF, WHO-DAS) | NA | Insured: 5.1% | NA |
| Guo (2017) | China | Cross-sectional | The Second China National Sample Survey on Disability (CNSSD) 2006 (national)  Population-based | Any health insurance | 3,848 Older adults with mental disabilities (≥60) | Mental (diagnosis by psychiatrist based on ICF, and ICD-10) | NA | **Sample**  Having insurance: 1328 (34.51%)  **Weighted**  Having Insurance: 682,000 (33.94%) | NA |
| Hao (2021) | Chonqing, China | Cross-sectional | Survey (facility)  Facility-based | Private | 75 people with mental disorder (≥18) | Mental – depression and anxiety disorder (psychiatrist diagnosis based on ICD-10) | 134 people without history of mental disorder | Private health insurance coverage: 36.7% vs 28.4% (P: 0.21) | Null |
| Lund (2019) | Ethiopia, India, Nepal, Nigeria, South Africa, Uganda | Cross-sectional | Survey (district in each country)  Facility-based | Any health insurance | 2339 people with mental neurological and substance use disorders (all ages) | Mental – depression, epilepsy, psychosis, alcohol use disorder (WHO mhGAP Intervention Guide, Composite International Diagnosis Interview [CIDI], AUDIT, PHQ-9) | 1982 individuals without MNS disorders | Depression: 3.5% (P<0.01)  Psychosis: 1.7% (P<0.01)  Alcohol-used disorder: 9.1% (P<0.05)  Epilepsy: 1.05% (P<0.01)  All MNS: 3.93% (P<0.05)  Control: 5.3% | Negative |
| Mai (2022) | China | Cross-sectional | Chinese Longitudinal Health Longevity Survey (CLHLS) 2018 (national)  Population-based | Any health insurance | 3980 older adults with limited ADL (≥65) | All type (ADL - bathing, dressing, toileting, indoor transferring, continence, and feeding) | NA | Having one insurance: 1,955 (49.12%)  Two or more insurance: 1,651 (41.48%) | NA |
| Medeiros (2021) | Brazil | Cross-sectional | National Health Survey 2013 (national)  Population-based | Any health insurance | 13,659 people with disabilities (all ages) | All types (Self-reported: do you have intellectual disability or physical disability or hearing impairment or visual impairment?) | NA | Insured: 26.3% (24.5 - 28.2) | NA |
| Moradi (2021) | Iran | Cross-sectional | Survey (national)  Population-based | Any health insurance | 2,006 children with disability (0 – 8) | Physical, mental (Registry of the Rehabilitation Department of the Welfare Organization of the selected provinces) | Children with disabilities without supplementary or other health insurance | Iranian health insurance: 32.8%  Armed force health insurance: 3.9%  Social security insurance: 54.1%  Other: 9.2%  Supplementary insurance: 14.7% | NA |
| Nartey (2018) | Sunyani Municipal District, Sunyani West District, Ghana | Cross-sectional | Survey (district)  Facility-based | National Health Insurance Scheme, Public | 542 people with mental illness (all ages) | Mental (clinical diagnosis) | People with mental illness without health insurance | NHIS: 4.77% | NA |
| Palmer (2011) | Vietnam | Cross-sectional | Vietnam National Health Survey 2001-2002 (national)  Population-based | Any health insurance | 4,905 individuals with disabilities (≥5) | All type (self-reported yes/no) | People without disability | 1. Public inpatient services insurance coverage: 23% vs 24% 2. Public inpatient fee waiver: 19% vs 9% 3. Outpatient services insurance coverage: 28% vs 15% 4. Outpatient services fee waiver: 40% vs 39% | 1. Null 2. Positive 3. Positive 4. Null |
| Palmer (2012) | Vietnam | Cross-sectional | Vietnam National Health Survey 2001-2002 (national)  Population-based | Compulsory Health Insurance, Public | 4,905 individuals with disabilities (≥5) | All type (self-reported yes/no) | People without disability | 1. Insurance card holder: 19.4% vs 18.8% 2. Compulsory health insurance: 11.5%* vs 8.9% 3. Social beneficiary: 6.4%* vs 1.1% 4. Employees: 5.2%* vs 7.8% 5. Pupil/student: 2.1%* vs 8.2%* 6. Health Insurance for the poor: 4.2%* vs 1.3%   *P<0.05 | 1. Null 2. Positive 3. Positive 4. Negative 5. Negative 6. Positive |
| Pengpid (2021) | India | Cross-sectional | Longitudinal Aging Study in India (LASI) wave 1 2017-2019 (national)  Population-based | Any health insurance | 6,573 older adults (≥60) | All types (ADL and IADL; disability defined as ≥1 ADL limitation) | Older adults without disability | Coverage among disability ≥1 limitation ADL: 19.5%  ≥1 IADL: 43.4%  AOR ≥1 ADL: 0.75 (0.65-0.96) P<0.001  AOR ≥1 IADL: 0.83 (0.74-0.94) P<0.01 | Negative |
| Shi (2019) | Shanghai, China | Cross-sectional | Electronic health record of mental hospitalization in public institution from 2013 – 2016 (district)  Facility-based | Urban social insurance for workers, Urban social insurance for citizens, New rural cooperative medical insurance, Public | 7,910 mental illness hospitalizations (all ages) | Mental (clinical diagnosis based on ICD-10, codes F00 to F99.999) | NA | Urban social insurance for workers: 50.59%  Urban social insurance for citizens: 13.43%  New rural cooperative medical insurance: 4.03%  Other insurance: 1.93% | NA |
| Van der Wielen (2018) | Ghana | Cross-sectional | The 2007-2008 Study on Global Aging and Adult Health (SAGE) and the 2012-2013 Ghanaian Living Standards Survey (GLSS) round 6 (national)  Population-based | National Health Insurance, Public | SAGE: 5,110  GLSS: 16,772  Adults (≥18) | All types (self-reported yes/no) | Adults without disability | 1. Age 18 – 49   Probability of never enrolled in NHIS: 0.83 (0.62 – 1.09)   1. Age ≥50   Probability of never enrolled in NHIS: 0.89 (0.66 – 1.18) | 1. Null 2. Null |
| Wiredu (2021) | Asokawa, Ghana | Cross-sectional | Survey (district)  Registry-based | National Health Insurance Scheme (NHIS), Public | 180 people with disabilities (all ages) | Vision and physical (registry of the Department of Social Welfare and Community Development) | NA | Enrolled in NHIS: 80% | NA |
| Yu (2018) | Urban China | Cross-sectional | The China National Health and Wellness Survey (NHWS) 2012-2013  National  Population-based | Any health insurance | 36,806 adults (≥18) | Mental – Generalized Anxiety Disorder (GAD) (self-reported diagnosis, GAD-7 screening) | Adults without GAD | Diagnosed: 94.9%  Screened positive: 91.5%  No GAD: 93.7% (P:0.001)  Probability of GAD (insured vs uninsured) AOR: 0.71 (0.59-0.86) | Positive |

Positive: People with disabilities have **higher** health insurance coverage than those without disability, OR people with disabilities were **more likely** to have health insurance than people without disability. Mixed results of positive and null are categorized as positive.

Negative: People with disabilities have **lower** health insurance coverage than those without disability, OR people with disabilities were **less likely** to have health insurance than people without disability. Mixed results of negative and null are categorized as negative.

Null: there is **no difference** in health insurance coverage between people with and without disability OR **no difference** in the probability of having health insurance between people with and without disability

Mixed: there is more than one measure showing **positive and negative** associations

Abbreviation: ADL: Activities of Daily Living; AOR: Adjusted Odds Ratio; APR: Adjusted Prevalence Ratio; IADL: Instrumental Activities of Daily Living
