# Supplementary material for: Access to health insurance amongst people with disabilities and its association with healthcare use, health status and financial protection in low- and middle-income countries: a systematic review
Source: Int J Equity Health. 2024 Dec 18;23:264. doi: 10.1186/s12939-024-02339-5 (PMC11658242; doi:10.1186/s12939-024-02339-5)
Supplement: Supplementary file 3 — Additional file 3. Association between health insurance and healthcare utilization amongst people with disabilities in LMICs. [file 12939_2024_2339_MOESM3_ESM.docx]

Additional file 3. Association between Health Insurance and Healthcare Utilization amongst People with Disabilities in LMICs

| **Citation** | **Setting** | **Study design** | **Source of data (representativeness)**  **Recruitment** | **Sample (age)** | **Health insurance,**  **Public/private**  **(targeting people with disabilities)** | **Type of disability (measurement)** | **Comparator** | **Outcome measure (recall period)** | **Results (95% CI)** | **Association**  **(Insured vs uninsured)** | **Association** | **Risk of bias** |
| --- | --- | --- | --- | --- | --- | --- | --- | --- | --- | --- | --- | --- |
| **General** |  |  |  |  |  |  |  |  |  |  |  |  |
| Chen & Ning (2022) | China | Quasi experimental | China Health and Retirement Longitudinal Study (CHARLS) 2011, 2013, 2015, 2018 (national)  Population-based | 67,640 observations of older adults (>45) | Long-term care insurance  Public (yes) | All types (Barthel Index – ADL function) | Older adults who were not receiving LTCI | 1. Outpatient visit (last month) 2. Number of hospitalization (last year) 3. Inpatient length of stay (last year) | 1. Reduced by 0.322 times (P<0.01) 2. Reduced by 0.158 times (P<0.01) 3. Reduced by 1.441 days (P<0.01) | 1. Negative 2. Negative 3. Negative | Negative | Low |
| Mai (2022) | China | Cross-sectional | Chinese Longitudinal Health Longevity Survey (CLHLS) 2018 (national)  Population-based | 3980 older adults with limited ADL (≥65) | No information  No information (no) | All types (ADL) | Older adults with limited ADL without health insurance | Access to healthcare services (unclear) | OR 1.2 (0.73-1.97) | Null | Null | Low |
| Palmer (2012) | Vietnam | Cross-sectional | Vietnam National Health Survey 2001-2022 (national)  Population-based | 4,905 people with disabilities (≥5) | Compulsory health insurance  Public (yes) | All types (self-reported yes/no: mobility, hearing, speaking, learning, mental, vision – only severe included) | People with disabilities without health insurance | 1. Public inpatient services (12 months) 2. Public outpatient services (1 month) | Regression coefficient   1. -0.002 SE 0.011 P>0.1 2. 0.045 SE 0.014 P<0.01 | 1. Null 2. Positive | Positive | Medium |
| Palmer (2014) | Vietnam | Cross-sectional | Vietnam Household Living Standards Survey (VHLSS) 2006 (national)  Population-based | 1,265 people with disabilities (all ages) | Social health insurance  Public (no) | All types (Washington Group Short Set) | People with disabilities without insurance | 1. Inpatient last 12 months 2. Outpatient last 1 month 3. Self-treatment last 1 month | Results from PSM   1. 0.093 (P<0.01) 2. 0.034 (P>0.1) 3. 0.033 (P>0.1)   Covariate matching   1. 0.111 (P<0.01) 2. 0.107 (P<0.01) 3. -0.042 (P<0.01) | Results from PSM   1. Positive 2. Null 3. Null   Covariate matching   1. Positive 2. Positive 3. Negative | Mixed | Low |
| Shiwakoti et al (2021) | Ilam District, Nepal | Cross-sectional | Survey,  (District)  Registry-based | 384 women with disabilities (15-49 years) | No information | All types (Washington Group Short Set) | Women with disabilities without health insurance | Any sexual and reproductive health services (unclear) | Crude OR: 1.2 (0.60 – 2.31) | Null | Null | High |
| **Disability-related** |  |  |  |  |  |  |  |  |  |  |  |  |
| Contentti (2019) | 12 Latin America countries | Cross-sectional | Survey in September and November of 2018 (regional)  Population-based | 1469 people with multiple sclerosis (all ages) | No information  No information (no) | Physical (clinical diagnosis for MS) | People with MS without health insurance | 1. Disease Modifying Therapy (DMT) past 12 months 2. MRI for diagnosis (lifetime) 3. Evoked Potential (lifetime) 4. Lumbar puncture (lifetime) 5. Received rehabilitation (unclear) | Probability uninsured vs insured   1. 142 (90.9%) vs 1111 (85.3%), P: 0.90 2. 154 (98.7%) vs 1297 (98.7), P:1 3. 115 (73.7%) vs 1006 (76.6%), P: 0.42 4. 106 (67.9) vs 898 (68.3), P: 0.92 5. 35 (22.4%) vs 239 (18.2%), P: 0.19 | 1. Null 2. Null 3. Null 4. Null 5. Null | Null | Low |
| El Sayed (2015) | 48 LMICs | Cross-sectional | The World Health Surveys (WHS) 2002-2004 (global)  Population-based | 197,914 households (≥18) | No information  No information (no) | Mental (self-reported diagnosis) | Adults with schizophrenia who are uninsured | Treatment uptake of persons with schizophrenia (self-reported - Lifetime) | Probability ratio (APR) uninsured vs insured   1. Male: 0.75 (0.51-1.11) 2. Male poorest: 0.96 (0.86-1.07) 3. Female: 0.57 (0.47-0.69) 4. Female poorest: 0.62 (0.56-0.69) | 1. Null 2. Null 3. Positive 4. Positive | Positive | Low |
| El Sayed (2015) | 48 LMICs | Cross-sectional | The World Health Surveys (WHS) 2002-2004 (global)  Population-based | 197,914 households (≥18) | No information  No information (no) | Mental (self-reported diagnosis) | Adults with depression who are uninsured | Treatment uptake of persons with depression (self-reported - Lifetime) | Probability ratio (APR) uninsured vs insured   1. Male: 0.59 (0.37-0.92) 2. Male poorest: 0.43 (0.22-0.85) 3. Female: 0.93 (0.80-1.08) 4. Female poorest: 0.71 (0.44-1.16) | 1. Positive 2. Positive 3. Null 4. Null | Positive |  |
| Fan (2023) | China | Quasi-experimental | Chinese Longitudinal Healthy Longevity Survey (CLHLS) 2011, 2014, 2018 (national)  Population-based | 2,544 older adults (≥65) | Long term care insurance  Public (yes) | All types (ADL, disability defined >1 limitation) | Older adults with disabilities without long-term care insurance | Unmet LTCI needs (unclear) | PSM-DiD coefficient:  -0.107, SE: 0.05, P<0.05 | Positive | Positive | Low |
| Guo (2015) | China | Cross-sectional | The Second China National Sample Survey on Disability (CNSSD) 2006 (national)  Population-based | 354,859 Older adults (≥60) | No information  No information (no) | All types (physician diagnosis using ICD-10, ICF, WHO-DAS) | Older adults with disabilities without health insurance | Healthcare utilization: curative care including surgeries and pharmaceutical treatments, auxiliary aids including assistive devices and services, and rehabilitation (unclear) | **Uninsured vs insured**  OR 0.80 (0.74-0.87) | Positive | Positive | Medium |
| Guo (2017) | China | Cross-sectional | The Second China National Sample Survey on Disability (CNSSD) 2006 (national)  Population-based | 3,848 Older adults with mental disabilities (≥60) | Medical insurance  No information (no) | Mental (diagnosis by psychiatrist based on ICF, and ICD-10) | Older adults with mental disability without medical insurance | Mental health service utilization (lifetime) | Insured vs insured  AOR: 1.45 (1.21-1.72), P<0.001 | Positive | Positive | Medium |
| Li (2013) | China | Cross-sectional | Population-based survey in 2006 (national)  Population-based | 2,526,145 adults (≥18) | Medical insurance  No information (no) | Mental (Screening and examination by psychiatrists using ICD-10 for diagnosis and WHO-DAS 2 to assess severity) | Adults with mental disability without health insurance | 1. Any mental health services (unclear) 2. Medical-pharmaceutical (unclear) 3. Rehabilitation (unclear) 4. Medical and rehabilitation (unclear) | Insured vs uninsured AOR   1. 1.39 (1.24-1.55) 2. 1.39 (1.24-1.56) 3. 1.10 (0.67-1.81) 4. 1.38 (1.12-1.70) | 1. Positive 2. Positive 3. Null 4. Positive | Positive | Medium |
| Machnicki (2011) | Zubizarreta Hospital, Buenos Aires, Argentina | Cross-sectional | Medical record (facility)  Facility-based | 92 patients with depression (55-80) | Private health insurance  Private (no) | Mental – depression (clinical diagnosis based on DSM IV and ICD-10) | Uninsured depression patients without insurance | Not receiving depression treatment – antidepressant (unclear) | Uninsured vs insured  AOR: 7.12 (1.88 – 26.86) | Positive | Positive | High |
| Medeiros (2021) | Brazil | Cross-sectional | National Health Survey 2013 (national)  Population-based | 13,659 people with disabilities (all ages) | No information  No information (no) | All types (Self-reported: do you have intellectual disability or physical disability or hearing impairment or visual impairment?) | People with disability without health insurance | Experience of attending at least one rehabilitation service (**lifetime)** | APR: 1.31 (1.15-1.49) P<0.001 | Positive | Positive | Medium |
| Nartey (2018) | Sunyani Municipal District, Sunyani West District, Ghana | Cross-sectional | Survey (district)  Facility-based | 542 people with mental illness (all ages) | Social Health Insurance  Public (no) | Mental (clinical diagnosis) | People with mental illness without health insurance | The use of mental health services as initial point of care – biomedical vs faith-based (unclear) | Probability of using biomedical vs faith-based between NHIS insured vs uninsured  AOR 2.47 (0.60 – 10.11) P: 0.20 | Null | Null | High |
| Nattaj (2017) | Sari Zare Hospital, Iran | Cross-sectional | Medical record from April 2007 to March 2012 (facility)  Facility-based | 2,397 patients at a psychiatric hospital (all ages) | No information  Public (no) | Mental (clinical diagnosis) | Patients with mental illness without health insurance | Length of hospitalization – days staying at hospital (lifetime) | RR insured vs uninsured: 0.71 (0.59 – 0.84) P<0.001 | Negative | Negative | High |
| Shi (2019) | Shanghai, China | Cross-sectional | Electronic health record of mental hospitalization in public institution from 2013 – 2016 (district)  Facility-based | 7,910 mental illness hospitalizations (all ages) | Urban social insurance for workers  Public (no) | Mental (clinical diagnosis based on ICD-10, codes F00 to F99.999) | People with mental illness without insurance | Mental health service utilization (2013-2016)   1. Community health centre vs **specialty health centre** 2. Secondary vs **specialty hospital** 3. Tertiary vs **specialty hospital** | Self-pay (uninsured) vs urban social insurance for workers AOR (95% CI)   1. 29.49 (16.16 – 53.81) P<0.0001 2. 3.49 (2.46 – 4.95) P<0.0001 3. 9.82 (7.04 – 13.71) P<0.0001 | 1. Positive 2. Positive 3. Positive | Positive | Medium |
|  |  |  |  |  |  |  | Urban social insurance for workers  Public – more generous | Mental health service utilization (2013-2016)   1. Community health centre vs **specialty health centre** 2. Secondary vs **specialty hospital** 3. Tertiary vs **specialty hospital** | Urban social insurance for citizens (inferior) vs urban social insurance for workers   1. 4.01 (1.99 – 8.07) P<0.0001 2. 0.99 (0.59 – 1.68) P: 0.992 3. 3.14 (1.83 – 5.37) P<0.0001 | 1. Positive 2. Null 3. Positive | Positive | Medium |
| Zhang (2018) | Guangzhou City, China | Retrospective cohort | Urban health insurance claims database 2010-2014 (facility)  Claim-based | 2,871 adults with schizophrenia (≥18) | Urban Employee Basic Medical Insurance (UEBMI)  Public (no) | Mental – schizophrenia (clinical diagnosis based on ICD-10, F20) | Urban Resident Basic Medical Insurance (URBMI) – less generous than UEBMI | 1. Total cost of service use (baseline) 2. Inpatient (baseline) 3. Outpatient (baseline) 4. Total cost of service use (first year) 5. Inpatient (first year) 6. Outpatient (first year) 7. Total cost of service use (second year) 8. Inpatient (second year) 9. Outpatient (second year) 10. Total cost of service use (third year) 11. Inpatient (third year) 12. Outpatient (third year) | UEBMI vs URBMI:   1. 42,543.1 vs 41,143.0 P: 0.021 2. 42,375.1 vs 40,917.3 P: 0.018 3. 168.0 vs 225.7 P: 0.031 4. 57,369.7 vs 54,058.6 P<0.001 5. 57,332.8 vs 53,998.5 P<0.001 6. 36.9 vs 60.1 P: 0.676 7. 57,326.6 vs 53,746.0 P<0.001 8. 57,287.6 vs 53,690.7 P<0.001 9. 39.0 vs 55.3 P: 0.356 10. 60,163.7 vs 51,875.6 P<0.001 11. 60,145.2 vs 51,804.5 P<0.001 12. 18.4 vs 71.1 P: 0.069 | 1. Positive 2. Positive 3. Positive 4. Positive 5. Positive 6. Null 7. Positive 8. Positive 9. Null 10. Positive 11. Positive 12. Null   Null on outpatient in the follow up years | Positive | Low |

Positive: Among people with disabilities, the insured have **higher** healthcare utilization than those uninsured. Mixed results of positive and null are categorized as positive.

Negative: Among people with disabilities, the insured have **lower** healthcare utilization than those uninsured. Mixed results of negative and null are categorized as negative.

Null: Among people with disabilities, there is **no difference** in healthcare utilization between the insured and uninsured people with disabilities

Mixed: There is more than one measure showing positive **and** negative association

Abbreviation: AOR: Adjusted Odds Ratio; APR: Adjusted Prevalence Ratio; DiD: Difference-in-Difference; PSM: Propensity Score Matching; RR: Risk Ratio
