# Supplementary material for: Access to health insurance amongst people with disabilities and its association with healthcare use, health status and financial protection in low- and middle-income countries: a systematic review
Source: Int J Equity Health. 2024 Dec 18;23:264. doi: 10.1186/s12939-024-02339-5 (PMC11658242; doi:10.1186/s12939-024-02339-5)
Supplement: Supplementary file 4 — Additional file 4. Association between health insurance and Out-of-pocket Payment (OOP), Catastrophic Health Expenditure (CHE) amongst people with disabilities in LMICs. [file 12939_2024_2339_MOESM4_ESM.docx]

Additional file 4. Association between Health Insurance and Out-of-pocket Payment (OOP) and Catastrophic Health Expenditure (CHE) amongst People with Disabilities in LMICs

| **Citation** | **Setting/ country** | **Study**  **design** | **Source of data (representativeness)**  **Recruitment** | **Health insurance** | **Samples (age)** | **Type of disability (measurement)** | **Comparator** | **Outcome measure** | **Results** | **Direction of Association**  **(insured vs uninsured)** | **Association**  **(insured vs uninsured)** | **Risk of bias** |
| --- | --- | --- | --- | --- | --- | --- | --- | --- | --- | --- | --- | --- |
| Chen & Ning (2022) | China | Quasi experimental | China Health and Retirement Longitudinal Study (CHARLS) 2011, 2013, 2015, 2018 (national)  Population-based | Long-term care insurance,  Public | 67,640 observations of older adults (>45) | All types (Barthel Index – ADL function) | Older adults with disabilities who were not receiving LTCI | 1. OOP on outpatient visit 2. OOP on hospitalization 3. Total of OOP on outpatient visits and hospitalization | Coefficient (DiD & PSM)   1. 49.589 (P>0.1) 2. -533.465 (P<0.05) 3. -512.562 (P<0.05) | 1. Null 2. Negative 3. Negative | Negative | Low |
| Guan (2019) | Beijing, China | Cross-sectional | Survey in 2016 (City)  Facility-based | Any insurance, public | 298 patients (all ages) | Vision impairment (clinical diagnosis, moderate VI or worse in both eyes; VA<6/18) | People with VI without URBMI/UEBMI (basic insurance) | Catastrophic health expenditure percentage among (30% threshold) | No insurance: 50%  New Cooperative Medical Scheme: 47.9%  Urban Resident Basic Medical Insurance (URBMI): 25%  Urban Employee Basic Medical Insurance (UEBMI): 30.9%  Government Medical Insurance: 16.7%  Commercial Medical Insurance: 21.4%  P: 0.008 | Positive | Positive | High |
| Moradi (2021) | Iran | Cross-sectional | Survey (national)  Population-based | Any health insurance | 2,006 children with disability (0 – 8) | Physical, mental (Registry of the Rehabilitation Department of the Welfare Organization of the selected provinces) | Children with disabilities without supplementary or other health insurance | Catastrophic health expenditure (40% threshold) | Uninsured vs insured  AOR 6.51 (95% CI: 3.69 – 8.24) | For the uninsured: Positive  Insured: Negative | Negative | Low |
| Palmer (2012) | Vietnam | Cross-sectional | Vietnam National Health Survey 2001-2022 (national)  Population-based | Compulsory Health Insurance, Public | 4,905 people with disabilities (≥5) | All types (self-reported yes/no: mobility, hearing, speaking, learning, mental, vision – only severe included) | Insured people without disability | 1. Public inpatient expenditure (12 months) 2. Public outpatient expenditure (1 month) | 1. 1297.919 vs 783.881 (P<0.05) 2. 23.725 vs 17.230 (P>0.05) | 1. Positive 2. Null | Positive | Medium |
|  |  |  |  |  |  |  | Uninsured people with disabilities | 1. Public inpatient expenditure (12 months) 2. Public outpatient expenditure (1 month) | 1. Coefficient -0.067 P:0.1 2. Coefficient 0.013 P>0.1 | 1. Null 2. Null | Null |  |
| Palmer (2014) | Vietnam | Cross-sectional | Vietnam Household Living Standards Survey (VHLSS) 2006 (national)  Population-based | Social health insurance,  Public | 1,265 people with disabilities (all ages) | All types (Washington Group Short Set) | People with disabilities without insurance | 1. Inpatient expenditure per visit 2. Outpatient expenditure per visit 3. Self-treatment per visit 4. CHE 10% 5. CHE 20% 6. CHE 40% 7. Poverty 8. Poverty net of health payment 9. Poverty differential | PSM   1. 103.062 (P>0.1) 2. -20.351 (P>0.1) 3. 2.088 (P>0.1) 4. -0.052 (P>0.1) 5. -0.090 (P<0.1) 6. -0.017 (P>0.1) 7. 0.009 (P>0.1) 8. 0.021 (P>0.1) 9. 0.012 (P>0.1)   Covariate matching   1. 213.435 (P<0.05) 2. 3.904 (P>0.1) 3. 1.111 (P>0.1) 4. -0.073 (P<0.01) 5. -0.066 (P<0.01) 6. 0.015 (P>0.1) 7. 0.042 (P<0.05) 8. 0.081 (P<0.05) 9. 0.039 (P<0.01) | PSM   1. Null 2. Null 3. Null 4. Null 5. Null 6. Null 7. Null 8. Null 9. Null   Covariate matching   1. Positive 2. Null 3. Null 4. Negative 5. Negative 6. Null 7. Positive 8. Positive 9. Positive | OOP (1-3): positive  CHE 10%: negative  CHE 20%: negative  CHE 40%: null  Poverty: Positive | Low |
| Zhang (2018) | Guangzhou City, China | Retrospective cohort | Urban health insurance claims database 2010-2014 (facility)  Claim-based | Urban Employee Basic Medical Insurance (UEBMI),  Public | 2,871 adults with schizophrenia (≥18) | Mental – schizophrenia (clinical diagnosis based on ICD-10, F20) | People with mental disabilities with Urban Resident Basic Medical Insurance (URBMI) – less generous | 1. OOP at baseline 2. OOP at 1 year follow up 3. OOP at 2 years follow up 4. OOP at 3 years follow up | UEBMI vs URBMI   1. 12.7% vs 13.5% P: 0.021 2. 10.7% vs 14.5% P<0.001 3. 10.7% vs 5.9% P<0.001 4. 9.5% vs 6.1% P<0.001 | 1. Negative 2. Negative 3. Positive 4. Positive | Mixed | Low |

Positive: Among people with disabilities, the insured have **higher** OOP/CHE than those uninsured. Or OOP/CHE is higher in insured people with disabilities than insured people without disability. Mixed results of positive and null are categorized as positive.

Negative: Among people with disabilities, the insured have **lower** OOP/CHE than those uninsured. Or OOP/CHE is lower in insured people with disabilities than insured people without disability. Mixed results of negative and null are categorized as negative.

Null: There is **no difference** in OOP/CHE between the insured and uninsured people with disabilities

Mixed: There is more than one measure showing positive **and** negative associations.

Abbreviation: AOR: Adjusted Odds Ratio; CHE: Catastrophic Health Expenditure; DiD: Difference-in-Difference; OOP: Out-of-pocket Payment; PSM: Propensity Score Matching; RR: Risk Ratio
